# Cycloaddition of 4-Acyl-1*H*-pyrrole-2,3-diones Fused at [*e*]-Side and Cyanamides: Divergent Approach to 4*H*-1,3-Oxazines

**DOI:** 10.3390/molecules27165257

**Published:** 2022-08-17

**Authors:** Ekaterina E. Khramtsova, Aleksandr D. Krainov, Maksim V. Dmitriev, Andrey N. Maslivets

**Affiliations:** Department of Chemistry, Perm State University, ul. Bukireva, 15, 614990 Perm, Russia

**Keywords:** acyl(quinoxalin-2-yl)ketene, cycloaddition, cyanamide, heterocumulene, hetero-Diels–Alder reaction, 4*H*-1,3-oxazine, thermolysis

## Abstract

4-Acyl-1*H*-pyrrole-2,3-diones fused at [*e*]-side with a heterocyclic moiety are suitable platforms for the development of a hetero-Diels–Alder-reaction-based, diversity-oriented approaches to series of skeletally diverse heterocycles. These platforms are known to react as oxa-dienes with dienophiles to form angular 6/6/5/6-tetracyclic alkaloid-like heterocycles and are also prone to decarbonylation at high temperatures resulting in generation of acyl(imidoyl)ketenes, bidentate aza- and oxa-dienes, which can react with dienophiles to form skeletally diverse products (angular tricyclic products or heterocyclic ensembles). Based on these features, we have developed an approach to two series of skeletally diverse 4*H*-1,3-oxazines (tetracyclic alkaloid-like 4*H*-1,3-oxazines and 5-heteryl-4*H*-1,3-oxazines) via a hetero-Diels–Alder reaction of 4-acyl-1*H*-pyrrole-2,3-diones fused at [*e*]-side with cyanamides. The products of these transformations are of interest for drug discovery, since compounds bearing 4*H*-1,3-oxazine moiety are extensively studied for inhibitory activities against anticancer targets.

## 1. Introduction

Diversity-oriented synthesis (DOS) is a strategy to access structurally diverse libraries of small molecules from a single set of reagents [1,2]. This approach allows efficient exploration of the chemical space for the development of new drugs [3,4].

**Figure 1:**
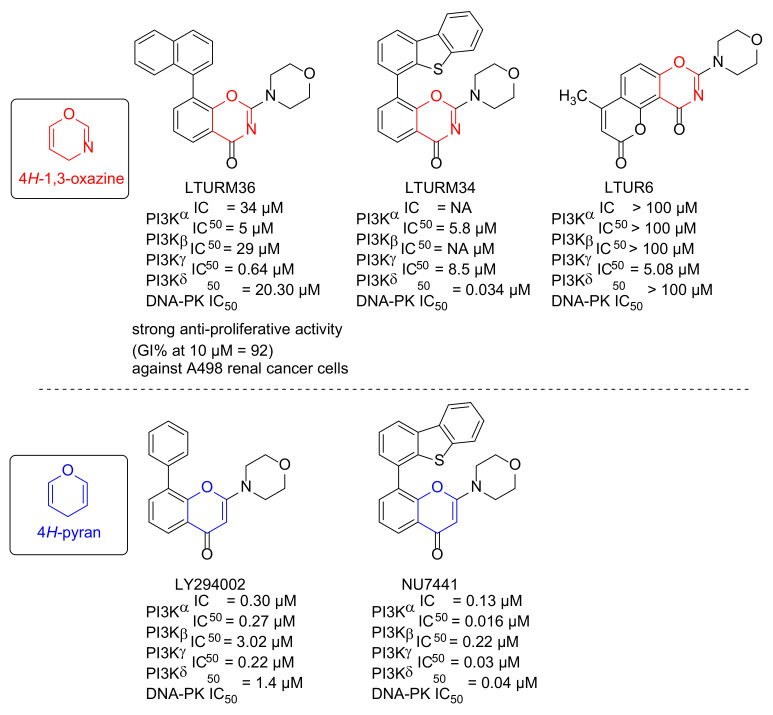
4*H*-1,3-Oxazine based inhibitors and their 4*H*-pyran analogs.

4*H*-1,3-Oxazine moiety is a valuable pharmacophore. Compounds bearing this moiety were extensively studied for inhibitory activities against various targets important for the anticancer therapy (Figure 1) [5,6,7,8,9,10]. By varying the substituents around the 4*H*-1,3-oxazine core, it was possible to tune the selectivity of inhibition (Figure 1) [5,6,7,8]. It should be mentioned that 4*H*-1,3-oxazine based inhibitors (LTURM34, LTUR6) were found to be more selective than their 4*H*-pyran analogs (NU7441, LY294002) (Figure 1) [6,9,11,12], which is preferable for the development of new drugs and inhibitors for biological assays.

The hetero-Diels–Alder reaction (HDA) is an atom and step economic synthetic strategy for assembling six-membered heterocycles [13]. HDA of oxa-dienes and nitriles affords 4*H*-1,3-oxazines [14,15,16,17,18,19].

**Scheme 1:**
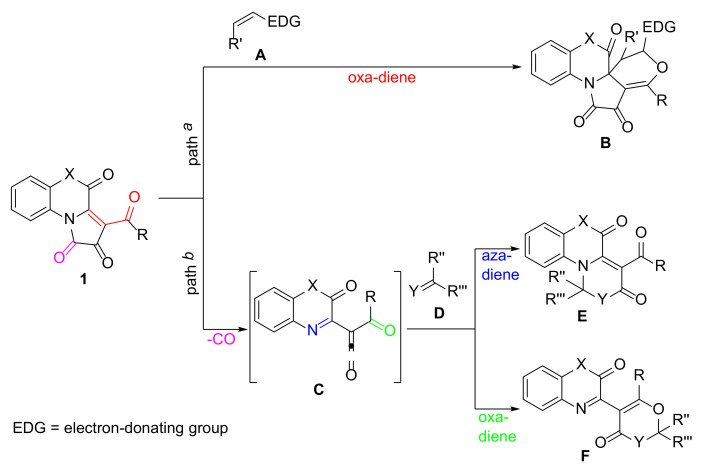
Modes of participation of FPDs **1** as heterodienes in HDA.

4-Acyl-1*H*-pyrrole-2,3-diones fused at [*e*]-side with a heterocyclic moiety (FPDs) **1** are well known to react as oxa-dienes with various electron-rich C=C dienophiles **A** to form angular 6/6/5/6-tetracyclic alkaloid-like pyrano[4,3-*b*]pyrroles **B** (Scheme 1, path *a*) [20,21,22,23]. At the same time, FPDs **1** are also known to readily undergo decarbonylation at temperatures above ~140 °C resulting in generation of highly reactive acyl(imidoyl)ketenes **C** (Scheme 1, path *b*) [24]. In turn, acyl(imidoyl)ketenes **C** are bidentate heterodienes prone to participate in HDA with heterodienophiles **D** (aldehydes, ketones, Schiff bases, carbodiimides, and etc.) both as aza- and oxa-dienes with the formation of corresponding angular heterocycles **E** or heterocyclic ensembles **F** [24,25]. Thus, FPDs **1** are suitable platforms for the development of an HDA-based DOS approaches to series of skeletally diverse heterocycles.

To the best of our knowledge, for today, there are no reported examples of a DOS, in which FPDs **1** react with a single dienophile (Scheme 1, **A** = **D**) both as oxa-dienes **1** (Scheme 1, path *a*) and heterodienes **C** (Scheme 1, path *b*). Herein, we present such an approach to two series of skeletally diverse 4*H*-1,3-oxazines via an HDA of FPDs **1** with cyanamides **2**.

## 2. Results and Discussion

Since acyl(imidoyl)ketenes **C** were reported not to react with common nitriles (benzonitrile, acetonitrile) [19,24,25], we decided to develop our DOS approach to 4*H*-1,3-oxazines utilizing so-called push–pull nitrile system, viz. cyanamides (aminonitriles) **2**, which are known to have higher reactivity in [4 + 2] cycloaddition reactions [26,27,28,29,30,31].

Initially, we tested the reaction of FPD **1a** with cyanamide **2a** in acetonitrile at room temperature (Table 1). According to UPLC-UV-MS data of the reaction mixture, the reaction proceeded very slowly. In a week, several unidentified side products were observed along with unreacted starting materials (conversion degree of FPD **1a** of ~20%). The UPLC-UV-MS yield of the desired product **3a** was ~10%. However, at elevating the reaction temperature up to 95 °C, the test reaction of FPD **1a** with cyanamide **2a** in acetonitrile proceeded smoothly and afforded the desired tetracyclic alkaloid-like 4*H*-1,3-oxazine **3a** in an isolated yield of 85% (Table 1, Entry **3a**). The reaction progress was monitored visually by the change of colour of the reaction mixture (FPD **1a** has a deep violet colour, and product **3a** is yellow). According to UPLC-UV-MS data of the reaction mixture, compound **3a** was formed as a single product, and no side products were observed. Product **3a** was isolated by a simple filtration directly from the reaction mixture. Since test results were satisfactory, we examined the substrate scope of this reaction by involving FPDs **1a–i**, bearing various acyl substituents R^1^ and heteroatoms X and cyanamides **2a–f**, bearing various substituents at amino nitrogen atom (Table 1).

Quinoxaline derivatives **3a–k** were prepared using acetonitrile as the reaction solvent and isolated by a simple filtration directly from the reaction mixture. For the synthesis of 1,4-benzoxazine derivatives (X = O), toluene was used as a reaction solvent since compounds **3l,m** were readily soluble in acetonitrile, and no precipitate was formed. In toluene compounds **3l,m** formed precipitates after cooling of the reaction mixtures to room temperature, which eased their isolation.

It was found that the studied reaction proceeded well both with 5-oxa (X = O) and 5-aza (X = NH, NPh, NMe) FPDs **1**. The reaction also worked well with various aryls and *tert*-butyl at acyl substituent R^1^ of FPDs **1**. Expectedly, the reaction of methoxy bearing FPD **1h** did not result in cycloadduct **3k**, since the methoxycarbonyl group COOMe is not electrophilic enough to participate in cycloaddition as a C=O part of the heterodiene system. The examined substituents in *N*,*N*-dialkylcyanamides **2a–d** did not affect the reaction noticeably. However, our attempts to involve *N*-arylcyanamides **2e,f** in HDA with FPDs **1a,l** were not successful. In this case, the reaction proceeded with formation of insoluble hard-to-purify compounds, whose structure we did not succeed to identify. We assume that in this case other reaction course could occur instead of the formation of the desired compounds **3o–q**, since *N*-arylcyanamides **2e,f** has lower nucleophilicity at C≡N nitrogen than *N*,*N*-dialkylcyanamides **2a–d**.

It is worthy of note that some of products **3** had a very low solubility in organic solvents all available to us. There were problems with acquisition on NMR spectra of such products, that’s why in some cases, we had to record solid-state NMR (ssNMR) spectra.

It should be mentioned that in case of the reaction of 4-nitrophenyl substituted FPD **1d** with 4-morpholinecarbonitrile **2b**, the desired product **3n** was observed only in trace amounts by UPLC-UV-MS of the reaction mixture. Prolongation of the reaction time (up to 14 days) and increasing the temperature (up to 120 °C) did not yield any positive results. We suppose that this phenomenon was caused by very low solubility of product **3n**, which, possibly, under the examined conditions (FPDs **1** were used as suspensions in acetonitrile), formed a protective insoluble layer on the surfaces of solid particles of FPD **1d** and, thus, prevented the reaction. It also should be mentioned that our attempts to perform the reaction of 4-nitrophenyl substituted FPD **1d** with carbonitrile **2b** in DMSO were also unsuccessful. This experiment was complicated by the fact that DMSO is a highly hygroscopic solvent and facilitated the hydrolysis reactions of the starting FPDs **1** and the products **3** (for hydrolysis studies of analogs of products **3,** see [22]). In the case of compound **1d**, NO_2_ substituent makes FPD **1d** very electrophilic and very reactive towards water.

Moreover, in the case of 1,4-benzoxazine products **3l,m** (X = O), there were problems with monitoring them with UPLC-UV-MS and HPLC-UV (acetonitrile–water as eluents). Chromatograms of the reaction mixtures and individual compounds **3l,m** (pure according to the NMR spectra) contained a lot of overlapped broad peaks, and the mass detector data showed signals of the desired products **3l,m** only in trace amounts. Furthermore, such problems were never observed with quinoxaline products **3a–j** (X = NH, NMe, NPh). We think that these could be explained by the occurrence of hydrolysis of compounds **3l,m** on the LC column due to the presence of an ester moiety in their structures, which is a common feature of such compounds [22].

**Scheme 2:**
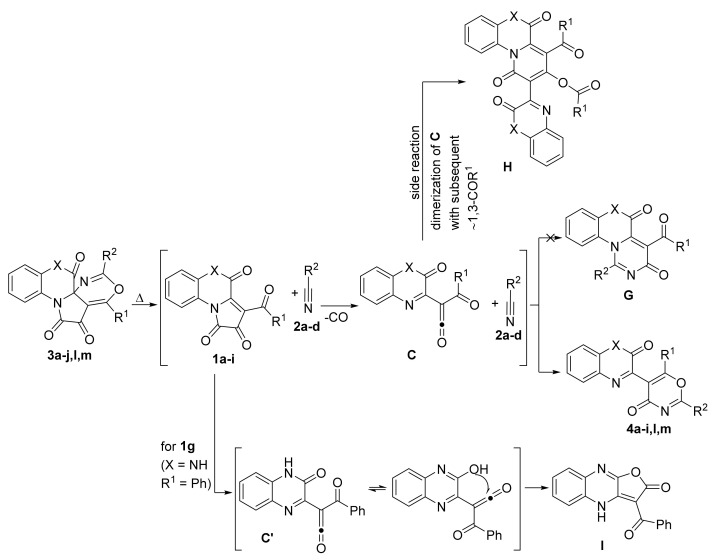
Plausible pathway of formation of compounds **4** and **G**.

The study of melting in a capillary of compounds **3a–i,l,m** revealed that under such conditions 5-heteryl-4*H*-1,3-oxazines **4a–i,l,m** (Table 2) were formed as sole products, and no regioisomeric pyrimidines **G** (Scheme 2) were observed (monitoring by UPLC-UV-MS). This transformation was then easily scaled up to 0.4 mmol (~200 mg) under solvent-free conditions. When scaling up, we found that an addition of small amounts (of about 0.1 equiv.) of the corresponding cyanamides **2a–d** was required to increase the isolated yields of compounds **4a–i,l,m** by reducing the side reactions leading to compounds **H** (monitoring by UPLC-UV-MS) (Scheme 2) characteristic of transformations involving in situ generation of acyl(imidoyl)ketenes **C** [24,32]. Compounds **4a–i,l,m** were readily isolated by simple recrystallization of the crude reaction mixtures. No effect of the examined substituents on the formation of compounds **4a–i,l,m** was observed. In the case of compound **3j** (X = NH), no compound **4j** was formed—instead of this compound, furoqinoxaline **I** was detected (monitoring by UPLC-UV-MS) (Scheme 2) [24,33].

We assume that the formation of compounds **4a–i,l,m** proceeded through three stages (Scheme 2). First, compounds **3a–i,l,m** underwent thermally initiated retro-HDA that afforded FPDs **1a–i** and cyanamides **2a–d**. Second, formed FPDs **1a–i** decarbonylated (the evolution of carbon monoxide was indicated by a gas detector tube) to generate acyl(imidoyl)ketenes **C**. And finally, acyl(imidoyl)ketenes **C** reacted as oxa-dienes with cyanamides **2a–d** to produce the desired 4*H*-1,3-oxazines **4a–i,l,m**. We suppose that ketenes **C** reacted with cyanamides **2a–d** exclusively as oxa-dienes, since this cycloaddition reaction proceeded via a charge-controlled polar transition state, as it was observed earlier in the reaction of ketenes **C** with carbodiimides [25].

To validate the proposed pathway of formation of compounds **4** (Scheme 2), we tested the one-pot solvent-free reaction of FPD **1a** with cyanamide **2b**. At heating of compound **1a** with cyanamide **2b** (reaction scale of 0.4 mmol, **1a**:**2b** reagents ratio of 1:1.1) at 235–240 °C, we found that compound **4b** was formed only in a yield of ~45% (monitoring by UPLC-UV-MS), which was much lower than in the case of decomposition of compound **3b**. We think that it was because of violation of heat and mass transfer processes during the solvent-free reaction of compounds **1a** and **2b**. These violations promoted the thermolytical side reactions leading to compounds **H** [24,32] (monitored by UPLC-UV-MS) and decreased the yield of compound **4b**. Thus, the development of a procedure to compounds **4** from the direct reaction of compounds **1** and **2** without isolation of compounds **3** is rather possible, but it requires additional optimization.

Then, to further validate the proposed pathway of formation of compounds **4** (Scheme 2), we performed the decomposition of compound **3b** in the presence of FPD **1b** at 240 °C and decomposition of compound **3a** in the presence of cyanamide **2b** at 240 °C and studied the obtained reaction mixtures by HPLC-UV. As a result, the decomposition of compound **3b** (R^1^ = Ph, R^2^ = morpholino) in the presence of FPD **1b** (R^1^ = 4-ClC_6_H_4_) at 240 °C afforded a mixture of compounds **4b** (R^1^ = Ph, R^2^ = morpholino) and **4e** (R^1^ = 4-ClC_6_H_4_, R^2^ = morpholino) along with a mixture of corresponding side products **H**. The decomposition of compound **3a** (R^1^ = Ph, R^2^ = NEt_2_) in the presence of cyanamide **2b** (R^2^ = morpholino) at 240 °C afforded a mixture of compounds **4a** (R^1^ = Ph, R^2^ = NEt_2_) and **4b** (R^1^ = Ph, R^2^ = morpholino) along with the corresponding side product **H**. These crossover experiments indirectly confirm that the proposed pathway of formation of compounds **4** (Scheme 2) includes retro-HDA stage and formation of acyl(imidoyl)ketenes **C**.

The structures of compounds **3a**, **3i**, **4b**, **4f**, **4g**, and **4i** were proved by single crystal X-ray analyses (CCDC 2192396 (**3a**), 2192397 (**3i**), 2192400 (**4b**), 2192399 (**4f**), 2192398 (**4g**), and 2196232 (**4i**)).

## 3. Materials and Methods

### 3.1. General Information

^1^H and ^13^C NMR spectra (Supplementary Materials) were acquired on a Bruker Avance III 400 HD spectrometer (Switzerland) (at 400 and 100 MHz, respectively) at 313 K in CDCl_3_ (stab. with Ag) or DMSO-*d*_6_ using the TMS or HMDS signal (in ^1^H NMR) or solvent residual signals (in ^13^C NMR, 77.00 for CDCl_3_, 39.51 for DMSO-*d*_6_; in ^1^H NMR, 7.26 for CDCl_3_, 2.50 for DMSO-*d*_6_) as internal standards. ^13^C ssNMR spectra were acquired on a Bruker Avance III 400 WB NMR spectrometer (Switzerland) (at 100 MHz). Melting points were measured on a Mettler Toledo MP70 apparatus (Switzerland). Elemental analyses were carried out on a Vario MICRO Cube analyzer (Germany). The reaction conditions were optimized using UPLC-UV-MS (Waters ACQUITY UPLC I-Class system (USA); Acquity UPLC BEH C18 column, grain size of 1.7 μm; acetonitrile–water (water containing 0.1% formic acid) as eluents; flow rate of 0.6 mL/min; ACQUITY UPLC PDA eλ Detector (wavelength range of 230–780 nm); Xevo TQD mass detector; electrospray ionization (ESI); positive and negative ion detection; ion source temperature of 150 °C; capillary voltage of 3500–4000 V; cone voltage of 20–70 V; vaporizer temperature of 200 °C) and HPLC-UV (Hitachi Chromaster Japan); NUCLEODUR C18 Gravity column (particle size 3 μm; eluent acetonitrile–water, flow rate 1.5 mL/min); Hitachi Chromaster 5430 diode array detector (λ 210–750 nm)). CO was indicated by gas detector tubes Gazoopredelitel GH-4 (USSR) (specifications 12.43.20-76). The single crystal X-ray analyses of compounds **3a**, **3i**, **4b**, **4f**, **4g**, and **4i** were performed on an Xcalibur Ruby diffractometer (Agilent Technologies, UK). The empirical absorption correction was introduced by multi-scan method using SCALE3 ABSPACK algorithm [34]. Using OLEX2 [35], the structures were solved with the SHELXS [36] program and refined by the full-matrix least-squares minimization in the anisotropic approximation for all non-hydrogen atoms with the SHELXL [37] program. Hydrogen atoms were positioned geometrically and refined using a riding model. Thin-layer chromatography (TLC) was performed on Merck silica gel 60 F_254_ plates using EtOAc/toluene, 1:5 *v/v*, toluene, EtOAc as eluents. Starting compounds **1a–j** were obtained according to reported procedures [25,33,38,39]. Toluene for procedures involving compounds **1** was dried over Na before the use. Acetonitrile for procedures involving compounds **1** was dried over molecular sieves 4Å before the use. All other solvents and reagents were purchased from commercial vendors and used as received. Procedures involving compounds **1, 3** were carried out in oven-dried glassware.

### 3.2. Synthetic Methods and Analytic Data of Compounds

#### 3.2.1. General Procedure to Compounds **3a–j,l,m**

A suspension of the corresponding FPD **1** (0.76 mmol) [25,33,38,39] and the corresponding cyanamide **2** (0.84 mmol) in 4 mL of a solvent (anhydrous acetonitrile (for **1a–h**) or anhydrous toluene (for **1i**)) was stirred and heated at 95 °C for 16 h (until the disappearance of the dark violet color of the compound **1**) in an oven-dried capped vial. Then the reaction mixture was cooled to room temperature, and the resulting precipitate was filtered off to afford the desired compound **3**. Compound **3** was pure enough and was used further without additional purification.

*5-(Diethylamino)-3,8-diphenyl-[1,3]oxazino[4′,5′:2,3]pyrrolo[1,2-a]quinoxaline-1,2,7(8H)-trione***(3a)**. Yield: 318 mg (85%); yellow solid; mp 200–204 °C (decomp.). ^1^H NMR (400 MHz, DMSO-*d*_6_): δ = 8.07 (m, 2 H), 7.82 (m, 1 H), 7.74 (m, 1 H), 7.66 (m, 2 H), 7.59 (m, 2 H), 7.51 (m, 1 H), 7.27–7.18 (m, 4 H), 6.43 (m, 1 H), 3.56–3.47 (m, 2 H), 3.36–3.26 (m, 2 H), 1.15 (t, *J* 7.1 Hz, 6 H) ppm. ^13^C NMR (100 MHz, DMSO-*d*_6_): δ = 177.0, 163.2, 159.4, 159.0, 150.5, 136.9, 133.7, 132.9, 129.9 (2C), 129.8 (2C), 128.8 (2C), 128.6 (2C), 128.5 (2C), 126.5, 123.0, 122.7, 122.2, 116.0, 104.1, 71.6, 42.4 (2C), 13.3 (2C) ppm. Anal. Calcd (%) for C_29_H_24_N_4_O_4_: C 70.72; H 4.91; N 11.38. Found: C 70.59; H 5.03; N 11.28. Crystal structure of compound **3a** was deposited at the Cambridge Crystallographic Data Centre with the deposition number CCDC 2192396. *Crystal Data of **3a***: C_29_H_24_N_4_O_4_, *M* = 492.52, triclinic, *a* = 9.507(2) Å, *b* = 10.481(2) Å, *c* = 13.277(4) Å, α = 74.93(2)°, β = 79.69(2)°, γ = 79.352(19)°, *V* = 1243.4(6) Å^3^, *T* = 295(2), space group *P*–1, *Z* = 2, μ(MoKα) = 0.090 mm^−1^. The final refinement parameters: *R*_1_ = 0.0697 [for observed 2640 reflections with *I* > 2σ(*I*)], *wR*_2_ = 0.1951 (for all independent 5764 reflections, *R*_int_ = 0.0711), *S* = 1.023. Largest diff. peak and hole 0.223 and −0.217 ēÅ^−3^.

*5-Morpholino-3,8-diphenyl-[1,3]oxazino[4′,5′:2,3]pyrrolo[1,2-a]quinoxaline-1,2,7(8H)-trione***(3b)**. Yield: 343 mg (89%); yellow solid; mp 223–224 °C (decomp.). ^1^H NMR (400 MHz, DMSO-*d*_6_): δ = 8.05 (m, 2 H), 7.81 (m, 1 H), 7.74 (m, 1 H), 7.67–7.57 (m, 4 H), 7.51 (m, 1 H), 7.27 (m, 2 H), 7.21(m, 2 H), 6.41 (m, 1 H), 3.65 (m, 4 H), 3.47 (m, 4 H) ppm. ^13^C ssNMR (100 MHz): δ = 177.0, 163.8, 160.5, 151.3, 137.3, 134.1, 129.8, 127.7, 126.2, 122.4, 115.2, 104.4, 72.3, 66.7, 44.9 ppm. Anal. Calcd (%) for C_29_H_22_N_4_O_5_: C 68.77; H 4.38; N 11.06. Found: C 68.59; H 4.23; N 11.08.

*5-(Dimethylamino)-3,8-diphenyl-[1,3]oxazino[4′,5′:2,3]pyrrolo[1,2-a]quinoxaline-1,2,7(8H)-trione***(3c)**. Yield: 275 mg (78%); yellow solid; mp 220–221 °C (decomp.). ^1^H NMR (400 MHz, DMSO-*d*_6_): δ = 8.08 (m, 2 H), 7.81 (m, 1 H), 7.74 (m, 1 H), 7.65 (m, 2 H), 7.59 (m, 2 H), 7.51 (m, 1 H), 7.27 (m, 2 H), 7.20 (m, 2 H), 6.41 (m, 1 H), 3.01 (s, 6 H) ppm. ^13^C ssNMR (100 MHz): δ = 176.8, 162.2, 151.5, 139.4, 136.3, 131.3, 129.9, 126.9, 124.0, 116.7, 104.2, 74.0, 36.8 ppm. Anal. Calcd (%) for C_27_H_20_N_4_O_4_: C 69.82; H 4.34; N 12.06. Found: C 70.03; H 4.35; N 12.42.

*3,8-Diphenyl-5-(piperidin-1-yl)-[1,3]oxazino[4′,5′:2,3]pyrrolo[1,2-a]quinoxaline-1,2,7(8H)-trione***(3d)**. Yield: 318 mg (83%); yellow solid; mp 221–224 °C (decomp.). ^1^H NMR (400 MHz, DMSO-*d*_6_): δ = 8.04 (m, 2 H), 7.82 (m, 1 H), 7.74 (m, 1 H), 7.65 (m, 2 H), 7.59 (m, 2 H), 7.51 (m, 1 H), 7.26–7.17 (m, 4 H), 6.41 (m, 1 H), 3.49 (m, 4 H), 1.57 (m, 6 H) ppm. ^13^C ssNMR (100 MHz): δ = 165.1, 160.9, 151.4, 135.8, 131.0, 128.7, 126.4, 123.1, 117.9, 104.2, 87.1, 48.2, 25.3, 21.9 ppm. Anal. Calcd (%) for C_30_H_24_N_4_O_4_: C 71.42; H 4.79; N 11.10. Found: C 71.21; H 4.82; N 11.10.

*3-(4-Chlorophenyl)-5-morpholino-8-phenyl-[1,3]oxazino[4′,5′:2,3]pyrrolo[1,2-a]quinoxaline-1,2,7(8H)-trione***(3e)**. Yield: 362 mg (88%); yellow solid; mp 230–235 °C (decomp.). ^1^H NMR (400 MHz, DMSO-*d*_6_): δ = 8.05 (m, 2 H), 7.81 (m, 1 H), 7.72 (m, 2 H), 7.59 (m, 2 H), 7.51 (m, 1 H), 7.27–7.16 (m, 4 H), 6.41 (m, 1 H), 3.65 (m, 4 H), 3.46 (m, 4 H) ppm. ^13^C ssNMR (100 MHz): δ = 178.3, 161.0, 159.1, 150.4, 141.3, 136.1, 132.7, 129.8, 127.9, 124.1, 115.0, 103.8, 66.3, 45.1 ppm. Anal. Calcd (%) for C_29_H_21_ClN_4_O_5_: C 64.39; H 3.91; N 10.36. Found: C 64.02; H 3.85; N 10.11.

*3-(4-Methoxyphenyl)-5-morpholino-8-phenyl-[1,3]oxazino[4′,5′:2,3]pyrrolo[1,2-a]quinoxaline-1,2,7(8H)-trione***(3f)**. Yield: 371 mg (91%); yellow solid; mp 242–244 °C (decomp.). ^1^H NMR (400 MHz, DMSO-*d*_6_): δ = 8.08 (m, 2 H), 7.81 (m, 1 H), 7.59 (m, 2 H), 7.50 (m, 1 H), 7.28–7.16 (m, 5 H), 6.40 (m, 1 H), 3.92 (s, 3 H), 3.65 (m, 4 H), 3.46 (m, 4 H) ppm. ^13^C ssNMR (100 MHz): δ = 178.7, 165.5, 160.2, 152.2, 134.0, 128.8, 125.9, 120.8, 114.9, 99.4, 72.7, 65.7, 56.6, 43.6 ppm. Anal. Calcd (%) for C_30_H_24_N_4_O_6_: C 67.16; H 4.51; N 10.44. Found: C 67.40; H 4.66; N 10.38.

*5-(Dimethylamino)-3-(4-nitrophenyl)-8-phenyl-[1,3]oxazino[4′,5′:2,3]pyrrolo[1,2-a]quinoxaline-1,2,7(8H)-trione***(3g)**. Yield: 314 mg (81%); yellow solid; mp 210–211 °C (decomp.). ^1^H NMR (400 MHz, DMSO-*d*_6_): δ = 8.46 (m, 2 H), 8.29 (m, 2 H), 7.81 (m, 1 H), 7.60 (m, 2 H), 7.51 (m, 1 H), 7.27 (m, 2 H), 7.22 (m, 2 H), 6.43 (m, 1 H), 3.02 (s, 6 H) ppm. ^13^C NMR (100 MHz, DMSO-*d*_6_): δ = 177.3, 163.2, 158.6, 156.6, 151.6, 149.9, 136.8, 134.0, 132.9, 131.4 (2C), 129.9 (2C), 128.9 (2C), 128.7, 126.6, 123.4, 123.1 (2C), 122.8, 122.1, 116.0, 106.1, 71.7, 37.2 (2C) ppm. Anal. Calcd (%) for C_27_H_19_N_5_O_6_: C 63.65; H 3.76; N 13.75. Found: C 63.87; H 4.06; N 13.91.

*8-Methyl-3-(4-methylphenyl)-5-morpholino-[1,3]oxazino[4′,5′:2,3]pyrrolo[1,2-a]quinoxaline-1,2,7(8H)-trione***(3h)**. Yield: 300 mg (86%); yellow solid; mp 208–210 °C (decomp.). ^1^H NMR (400 MHz, DMSO-*d*_6_): δ = 7.96 (m, 2 H), 7.74 (m, 1 H), 7.46 (m, 2 H), 7.42–7.35 (m, 2 H), 7.25 (m, 1 H), 3.60 (m, 4 H), 3.42–3.32 (m, 7 H), 2.47 (s, 3 H) ppm. ^13^C NMR (100 MHz, DMSO-*d*_6_): δ = 176.5, 163.6, 159.9, 159.0, 151.2, 144.7, 132.0, 130.2 (2 C), 129.0 (2 C), 126.9, 125.7, 122.9, 122.7, 122.5, 115.5, 103.5, 71.3, 65.2 (2 C), 44.7 (2 C), 29.5, 21.3 ppm. Anal. Calcd (%) for C_25_H_22_N_4_O_5_: C 65.49; H 4.84; N 12.22. Found: C 65.57; H 4.96; N 11.99.

*3-(tert-Butyl)-5-morpholino-8-phenyl-[1,3]oxazino[4′,5′:2,3]pyrrolo[1,2-a]quinoxaline-1,2,7(8H)-trione***(3i)**. After cooling the reaction mixture, no precipitate was formed. As such, the reaction solvent (acetonitrile) was removed on a rotary evaporator. The resulting solid was dissolved in toluene (2 mL). Then, petroleum ether (bp 70–100 °C) (6 mL) was added to the toluene solution, and the resulting precipitate was filtered off to afford compound **3i**. Yield: 310 mg (79%, solvate with toluene); yellow solid; mp 176–179 °C (decomp.). ^1^H NMR (400 MHz, DMSO-*d*_6_): δ = 7.75 (m, 1 H), 7.58 (m, 2 H), 7.50 (m, 1 H), 7.24–7.14 (m, 4 H), 6.38 (m, 1 H), 3.60 (m, 4 H), 3.35 (m, 4 H), 1.41 (s, 9 H) ppm. ^13^C NMR (100 MHz, DMSO-*d*_6_): δ = 178.2, 172.9, 162.8, 158.4, 151.8, 136.9, 132.9, 129.8, 128.8 (2 C), 128.6 (2 C), 126.5, 123.1, 122.7, 122.0, 116.0, 103.1, 71.5, 65.2 (2 C), 44.8 (2 C), 37.9, 26.4 (3 C) ppm. Anal. Calcd (%) for 3C_27_H_26_N_4_O_5_ · C_7_H_8_: C 68.12; H 5.59; N 10.83. Found: C 67.81; H 5.24; N 10.50. Crystal structure of compound **3i** was deposited at the Cambridge Crystallographic Data Centre with the deposition number CCDC 2192397. *Crystal Data of **3i***: C_27_H_26_N_4_O_5_, *M* = 486.52, monoclinic, *a* = 18.457(11) Å, *b* = 9.5254(17) Å, *c* = 15.057(6) Å, β = 112.96(6) °, *V* = 2438(2) Å^3^, *T* = 295(2), space group *P*2_1_/c, *Z* = 4, μ(Mo Kα) = 0.093 mm^−1^. The final refinement parameters: *R*_1_ = 0.1170 [for observed 2112 reflections with *I* > 2σ(*I*)], *wR*_2_ = 0.3010 (for all independent 6027 reflections, *R*_int_ = 0.0895), *S* = 1.049. Largest diff. peak and hole 0.315 and –0.257 ēÅ^−3^.

*5-Morpholino-3-phenyl-[1,3]oxazino[4′,5′:2,3]pyrrolo[1,2-a]quinoxaline-1,2,7(8H)-trione***(3j)**. Yield: 301 mg (92%); yellow solid; mp 201–203 °C (decomp.). ^1^H NMR (400 MHz, DMSO-*d*_6_): δ = 10.76 (s, 1 H), 8.05 (m, 2 H), 7.73 (m, 2 H), 7.65 (m, 2 H), 7.29 (m, 1 H), 7.18–7.09 (m, 2 H), 3.60 (m, 4 H), 3.40 (m, 4 H) ppm. ^13^C ssNMR (100 MHz): δ = 164.8, 160.9, 158.3, 149.5, 144.6, 139.4, 133.0, 128.4, 121.6, 117.9, 105.6, 89.4, 67.6, 45.6 ppm. Anal. Calcd (%) for C_23_H_18_N_4_O_5_: C 64.18; H 4.22; N 13.02. Found: C 64.01; H 4.25; N 12.99.

*5-Morpholino-3-phenyl-7H-benzo[5′,6′][1,4]oxazino[4′,3′:1,2]pyrrolo[2,3-d][1,3]oxazine-1,2,7-trione***(3l)**. Yield: 266 mg (81%); yellow solid; mp 214–219 °C (decomp.). ^1^H NMR (400 MHz, DMSO-*d*_6_): δ = 8.06 (m, 2 H), 7.82–7.73 (m, 2 H), 7.66 (m, 2 H), 7.43–7.31 (m, 3 H), 3.62 (m, 4 H), 3.43 (m, 4 H) ppm. ^13^C NMR (100 MHz, DMSO-*d*_6_): δ = 176.3, 161.1, 159.4, 158.7, 151.9, 143.0, 133.9, 130.2 (2 C), 128.5 (2 C), 128.1, 127.2, 124.6, 122.8, 121.3, 116.5, 103.5, 70.5, 65.2 (2 C), 44.7 (2 C) ppm. Anal. Calcd (%) for C_23_H_17_N_3_O_6_: C 64.04; H 3.97; N 9.74. Found: C 64.32; H 4.04; N 9.50.

*5-(Dimethylamino)-3-phenyl-7H-benzo[5′,6′][1,4]oxazino[4′,3′:1,2]pyrrolo[2,3-d][1,3]oxazine-1,2,7-trione***(3m)**. Yield: 219 mg (74%); yellow solid; mp 225–230 °C (decomp.). ^1^H NMR (400 MHz, DMSO-*d*_6_): δ = 8.08 (m, 2 H), 7.81–7.73 (m, 2 H), 7.67 (m, 2 H), 7.42–7.32 (m, 3 H), 2.97 (s, 6 H) ppm. ^13^C NMR (100 MHz, DMSO-*d*_6_): δ = 176.4, 161.3, 159.5, 158.7, 152.3, 143.0, 133.9, 130.1 (2 C), 128.5 (2 C), 128.1, 127.1, 124.5, 122.8, 121.3, 116.5, 103.5, 70.5, 37.0 (2 C) ppm. Anal. Calcd (%) for C_21_H_15_N_3_O_5_: C 64.78; H 3.88; N 10.79. Found: C 64.52; H 4.01; N 10.60.

#### 3.2.2. General Procedure to Compounds **4a–i,l,m**

A mixture of the corresponding compound **3** (0.4 mmol) and the corresponding cyanamide **2** (0.04 mmol) was put into an oven-dried tube, pressed slightly, and then heated in a metal bath at 190–245 °C (the temperature for each compound is given in Table 2; caution: CO evolves during the reaction) for 3 min. The reaction mixture was cooled to room temperature and recrystallized from about 3 mL of a solvent (acetonitrile (for **3a–h**) or toluene (for **3l,m**)) to give the appropriate compound **4**. In the case of compound **3i**, the reaction mixture was cooled to room temperature, dissolved in 1 mL of ethyl acetate. Then, 5 mL of *n*-hexane were added to it, and the resulting precipitate was filtered off to afford compound **4i**.

*2-(Diethylamino)-5-(3-oxo-4-phenyl-3,4-dihydroquinoxalin-2-yl)-6-phenyl-4H-1,3-oxazin-4-one***(4a)**. Yield: 132 mg (71%); yellow solid; mp 271–273 °C. ^1^H NMR (400 MHz, DMSO-*d*_6_): δ = 7.80 (m, 1 H), 7.70–7.32 (m, 12 H), 6.65 (m, 1 H), 3.58 (m, 4 H), 1.25 (m, 6 H) ppm. ^13^C NMR (100 MHz, DMSO-*d*_6_): δ = 166.1, 158.0, 156.9, 154.1, 153.2, 135.2, 133.9, 131.9, 131.1, 130.9, 130.2, 130.1 (2 C), 129.4, 129.3, 128.9 (2 C), 128.3, 128.2, 127.5 (2 C), 123.8, 115.2, 114.0, 41.7 (2 C), 12.3 (2 C) ppm. Anal. Calcd (%) for C_28_H_24_N_4_O_3_: C 72.40; H 5.21; N 12.06. Found: C 72.20; H 5.17; N 11.93.

*2-Morpholino-5-(3-oxo-4-phenyl-3,4-dihydroquinoxalin-2-yl)-6-phenyl-4H-1,3-oxazin-4-one***(4b)**. Yield: 163 mg (85%); yellow solid; mp 285–287 °C. ^1^H NMR (400 MHz, CDCl_3_): δ = 7.87 (m, 1 H), 7.58–7.26 (m, 11 H), 7.06 (br.s, 1 H), 6.67 (m, 1 H), 3.78 (m, 8 H) ppm. ^13^C NMR (100 MHz, CDCl_3_): δ = 167.2, 159.6, 157.1, 153.6, 153.5, 135.6, 134.6, 132.8, 131.1, 130.9, 130.6, 130.2 (2 C), 130.0, 129.3, 128.7 (2 C), 128.6, 128.2, 128.1 (2 C), 123.7, 115.4, 114.8, 66.3 (2 C), 44.5 (2 C) ppm. Anal. Calcd (%) for C_28_H_22_N_4_O_4_: C 70.28; H 4.63; N 11.71. Found: C 70.38; H 4.41; N 11.53. Crystal structure of compound **4b** was deposited at the Cambridge Crystallographic Data Centre with the deposition number CCDC 2192400. *Crystal Data of **4b**:* C_28_H_22_N_4_O_4_, *M* = 478.49, monoclinic, *a* = 12.995(3) Å, *b* = 9.3586(15) Å, *c* = 19.719(6) Å, β = 105.98(3) °, *V* = 2305.5(10) Å^3^, *T* = 295(2), space group *P*2_1_/c, *Z* = 4, μ(Mo Kα) = 0.094 mm^−1^. The final refinement parameters: *R*_1_ = 0.0498 [for observed 3563 reflections with *I* > 2σ(*I*)], *wR*_2_ = 0.1360 (for all independent 5442 reflections, *R*_int_ = 0.0265), *S* = 1.043. Largest diff. peak and hole 0.191 and –0.230 ēÅ^−3^.

*2-(Dimethylamino)-5-(3-oxo-4-phenyl-3,4-dihydroquinoxalin-2-yl)-6-phenyl-4H-1,3-oxazin-4-one***(4c)**. Yield: 143 mg (78%, solvate with acetonitrile); yellow solid; mp 279–280 °C. ^1^H NMR (400 MHz, DMSO-*d*_6_): δ = 7.81 (m, 1 H), 7.70–7.30 (m, 12 H), 6.63 (m, 1 H), 3.16 (m, 6 H) ppm. ^13^C NMR (100 MHz, DMSO-*d*_6_): δ = 166.1, 157.8, 157.7, 154.1, 153.1, 135.2, 133.9, 131.8, 131.1, 130.9, 130.2, 130.1 (2 C), 129.4, 129.3, 128.9 (2 C), 128.3, 128.2, 127.6 (2 C), 123.8, 115.2, 113.8, 37.0, 35.9 ppm. Anal. Calcd (%) for 2C_26_H_20_N_4_O_3_ C_2_H_3_N: C 70.96; H 4.74; N 13.79. Found: C 71.32; H 4.69; N 13.61.

*5-(3-Oxo-4-phenyl-3,4-dihydroquinoxalin-2-yl)-6-phenyl-2-(piperidin-1-yl)-4H-1,3-oxazin-4-one***(4d)**. Yield: 147 mg (77%); yellow solid; mp 275–277 °C. ^1^H NMR (400 MHz, DMSO-*d*_6_): δ = 7.80 (m, 1 H), 7.70–7.30 (m, 12 H), 6.64 (m, 1 H), 3.69 (m, 4 H), 1.66 (m, 6 H) ppm. ^13^C NMR (100 MHz, DMSO-*d*_6_): δ = 166.2, 157.9, 156.5, 154.0, 153.1, 135.2, 133.9, 131.8, 131.1, 130.9, 130.2, 130.1 (2 C), 129.4, 129.3, 128.9 (2 C), 128.3, 128.2, 127.6 (2 C), 123.8, 115.2, 113.9, 44.7 (2 C), 24.8 (2 C), 23.4 ppm. Anal. Calcd (%) for C_29_H_24_N_4_O_3_: C 73.09; H 5.08; N 11.76. Found: C 73.41; H 5.09; N 11.92.

*6-(4-Chlorophenyl)-2-morpholino-5-(3-oxo-4-phenyl-3,4-dihydroquinoxalin-2-yl)-4H-1,3-oxazin-4-one***(4e)**. Yield: 176 mg (86%); yellow solid; mp 311–315 °C. ^1^H NMR (400 MHz, DMSO-*d*_6_): δ = 7.81 (m, 1 H), 7.70–7.48 (m, 8 H), 7.38 (m, 3 H), 6.64 (m, 1 H), 3.71 (m, 8 H) ppm. ^13^C ssNMR (100 MHz): δ = 167.0, 157.3, 155.3, 152.6, 138.7, 134.2, 131.4, 129.5, 127.6, 124.6, 115.6, 65.6, 42.9 ppm. Anal. Calcd (%) for C_28_H_21_ClN_4_O_4_: C 65.56; H 4.13; N 10.92. Found: C 65.34; H 4.11; N 10.59.

*6-(4-Methoxyphenyl)-2-morpholino-5-(3-oxo-4-phenyl-3,4-dihydroquinoxalin-2-yl)-4H-1,3-oxazin-4-one***(4f)**. Yield: 185 mg (91%); yellow solid; mp 293–295 °C. ^1^H NMR (400 MHz, DMSO-*d*_6_): δ = 7.82 (m, 1 H), 7.71–7.48 (m, 6 H), 7.42–7.35 (m, 3 H), 7.01 (m, 2 H), 6.64 (m, 1 H), 3.77 (s, 3 H), 3.72 (m, 8 H) ppm. ^13^C NMR (100 MHz, DMSO-*d*_6_): δ = 166.4, 161.4, 157.8, 156.9, 154.2, 153.1, 135.3, 134.0, 131.9, 130.9, 130.1 (2 C), 129.4 (4 C), 129.3, 128.2, 123.8, 122.1, 115.2, 114.4 (2 C), 112.7, 65.3 (2 C), 55.3, 43.9 (2 C) ppm. Anal. Calcd (%) for C_29_H_24_N_4_O_5_: C 68.49; H 4.76; N 11.02. Found: C 68.78; H 4.71; N 11.08. Crystal structure of compound **4f** was deposited at the Cambridge Crystallographic Data Centre with the deposition number CCDC 2192399. *Crystal Data of **4f**:* C_29_H_24_N_4_O_5_, *M* = 508.52, monoclinic, *a* = 13.137(2) Å, *b* = 10.009(3) Å, *c* = 19.142(4) Å, β = 101.80(2) °, *V* = 2463.8(10) Å^3^, *T* = 295(2), space group *P*2_1_/c, *Z* = 4, μ(Mo Kα) = 0.096 mm^−1^. The final refinement parameters: *R*_1_ = 0.0651 [for observed 3086 reflections with *I* > 2σ(*I*)], *wR*_2_ = 0.2110 (for all independent 5836 reflections, *R*_int_ = 0.0479), *S* = 1.036. Largest diff. peak and hole 0.324 and −0.238 ēÅ^−3^.

*2-(Dimethylamino)-6-(4-nitrophenyl)-5-(3-oxo-4-phenyl-3,4-dihydroquinoxalin-2-yl)-4H-1,3-oxazin-4-one***(4g)**. Yield: 152 mg (79%); pale yellow solid; mp 298–300 °C. ^1^H NMR (400 MHz, DMSO-*d*_6_): δ = 8.30 (m, 2 H), 7.87 (m, 2 H), 7.80 (m, 1 H), 7.70–7.34 (m, 7 H), 6.65 (m, 1 H), 3.17 (m, 6 H) ppm. ^13^C NMR (100 MHz, DMSO-*d*_6_): δ = 170.1, 165.6, 157.6, 155.9, 153.3, 153.1, 148.5, 136.0, 135.2, 134.1, 131.9, 131.1, 130.1 (2 C), 129.5, 129.4, 129.2 (2 C), 128.1, 124.0 (2 C), 123.8, 115.5, 115.3, 37.1, 36.0 ppm. Anal. Calcd (%) for C_26_H_19_N_5_O_5_: C 64.86; H 3.98; N 14.55. Found: C 64.89; H 4.01; N 14.19. Crystal structure of compound **4g** was deposited at the Cambridge Crystallographic Data Centre with the deposition number CCDC 2192398. *Crystal Data of **4g***: C_26_H_19_N_5_O_5_, *M* = 481.46, triclinic, *a* = 9.8633(13) Å, *b* = 10.7810(14) Å, *c* = 11.3896(14) Å, α = 74.960(11) °, β = 86.914(10) °, γ = 75.606(11) °, *V* = 1132.8(3) Å^3^, *T* = 295(2), space group *P*–1, *Z* = 2, μ(MoKα) = 0.101 mm^−1^. The final refinement parameters: *R*_1_ = 0.0530 [for observed 3900 reflections with *I* > 2σ(*I*)], *wR*_2_ = 0.1505 (for all independent 5227 reflections, *R*_int_ = 0.0300), *S* = 1.043. Largest diff. peak and hole 0.310 and −0.309 ēÅ^−3^.

*5-(4-Methyl-3-oxo-3,4-dihydroquinoxalin-2-yl)-6-(4-methylphenyl)-2-morpholino-4H-1,3-oxazin-4-one***(4h)**. Yield: 141 mg (82%); yellow solid; mp 271–273 °C. ^1^H NMR (400 MHz, DMSO-*d*_6_): δ = 7.75 (m, 1 H), 7.69 (m, 1 H), 7.61 (m, 1 H), 7.43–7.36 (m, 3 H), 7.20 (m, 2 H), 3.72 (m, 8 H), 3.66 (s, 3 H), 2.26 (s, 3 H) ppm. ^13^C ssNMR (100 MHz): δ = 167.4, 165.9, 162.6, 161.3, 156.4, 152.6, 150.6, 143.5, 142.4, 131.6, 130.1, 127.6, 122.8, 115.0, 113.6, 110.6, 66.8, 44.5, 28.4, 22.7, 20.6 ppm. Anal. Calcd (%) for C_24_H_22_N_4_O_4_: C 66.97; H 5.15; N 13.02. Found: C 66.81; H 4.99; N 13.19.

*6-(tert-Butyl)-2-morpholino-5-(3-oxo-4-phenyl-3,4-dihydroquinoxalin-2-yl)-4H-1,3-oxazin-4-one***(4i)**. Yield: 154 mg (84%); pale yellow solid; mp 160–162 °C. ^1^H NMR (400 MHz, DMSO-*d*_6_): δ = 7.88 (m, 1 H), 7.69–7.58 (m, 4 H), 7.52 (m, 1 H), 7.43–7.32 (m, 3 H), 6.65 (m, 1 H), 3.71 (m, 4 H), 3.65 (m, 4 H), 1.19 (s, 9 H) ppm. ^13^C NMR (100 MHz, DMSO-*d*_6_): δ = 167.1 (2 C), 157.2, 155.2, 153.3, 135.3, 133.9, 131.5, 130.8, 130.2 (2 C), 129.4 (2 C), 129.3, 128.2, 123.8, 115.2, 112.9, 65.2 (2 C), 43.8 (2 C), 37.1, 28.0 (3 C) ppm. Anal. Calcd (%) for C_26_H_26_N_4_O_4_: C 68.11; H 5.72; N 12.22. Found: C 68.27; H 5.59; N 12.30. Crystal structure of compound **4i** was deposited at the Cambridge Crystallographic Data Centre with the deposition number CCDC 2196232. *Crystal Data of **4i***: C_26_H_26_N_4_O_4_, *M* = 458.51, orthorhombic, *a* = 17.435(7) Å, *b* = 15.185(4) Å, *c* = 17.942(4) Å, *V* = 4750(3) Å^3^, *T* = 295(2), space group *P*bca, *Z* = 8, μ(MoKα) = 0.088 mm^−1^. The final refinement parameters: *R*_1_ = 0.0984 [for observed 1995 reflections with *I* > 2σ(*I*)], *wR*_2_ = 0.2775 (for all independent 5965 reflections, *R*_int_ = 0.1670), *S* = 1.025. Largest diff. peak and hole 0.255 and −0.215 ēÅ^−3^.

*3-(2-Morpholino-4-oxo-6-phenyl-4H-1,3-oxazin-5-yl)-2H-benzo[b][1,4]oxazin-2-one***(4l)**. Yield: 105 mg (65%); yellow solid; mp 208–211 °C. ^1^H NMR (400 MHz, DMSO-*d*_6_): δ = 7.73 (m, 1 H), 7.65 (m, 3 H), 7.54–7.42 (m, 5 H), 3.72 (m, 8 H) ppm. ^13^C NMR (100 MHz, DMSO-*d*_6_): δ = 166.0, 159.1, 156.9, 151.4, 150.4, 146.1, 132.2, 131.5, 130.5, 129.4, 129.0, 128.9 (2 C), 128.1 (2 C), 125.8, 116.5, 112.5, 65.2 (2 C), 44.0 (2 C) ppm. Anal. Calcd (%) for C_22_H_17_N_3_O_5_: C 65.50; H 4.25; N 10.42. Found: C 65.67; H 4.12; N 10.32.

*3-(2-(Dimethylamino)-4-oxo-6-phenyl-4H-1,3-oxazin-5-yl)-2H-benzo[b][1,4]oxazin-2-one***(4m)**. Yield: 98 mg (68%); yellow solid; mp 159–163 °C. ^1^H NMR (400 MHz, DMSO-*d*_6_): δ = 7.73 (m, 1 H), 7.64 (m, 3 H), 7.54–7.42 (m, 5 H), 3.17 (m, 6 H) ppm. ^13^C NMR (100 MHz, DMSO-*d*_6_): δ = 166.0, 158.9, 157.8, 151.4, 150.6, 146.0, 132.2, 131.4, 130.5, 129.5, 129.0, 128.9 (2 C), 128.0 (2 C), 125.8, 116.5, 112.2, 37.1, 36.0 ppm. Anal. Calcd (%) for C_20_H_15_N_3_O_4_: C 66.48; H 4.18; N 11.63. Found: C 66.09; H 4.00; N 11.71.

#### 3.2.3. Procedure to Compound **I**

Compound **3j** (22 mg, 0.05 mmol) was put into an oven-dried tube, pressed slightly, and heated in a metal bath at 210–215 °C (caution: CO evolves during the reaction) for 3 min. The reaction mixture was cooled to room temperature and recrystallized from about 5 mL of 1,4-dioxane to give compound **I**.

*3-Benzoylfuro[2,3-b]quinoxalin-2(4H)-one***(I)** [33]. Yield: 9.9 mg (68%); yellow solid; mp 273–274 °C (reported mp 274–275 °C [33]). ^1^H NMR (400 MHz, DMSO-*d*_6_): δ = 13.98 (br.s, 1 H), 8.20 (m, 1 H), 7.84 (m, 3 H), 7.66–7.48 (m, 5 H) ppm. ^13^C NMR (100 MHz, DMSO-*d*_6_): δ = 188.4, 163.9, 154.6, 141.9, 137.8, 134.6, 131.8, 128.6, 128.4 (2 C), 128.3, 127.9, 127.7 (2 C), 126.4, 118.6, 91.6 ppm. Anal. Calcd (%) for C_17_H_10_N_2_O_3_: C 70.34; H 3.47; N 9.65. Found: C 70.53; H 3.37; N 9.71.

## 4. Conclusions

In conclusion, we have developed a novel diversity-oriented approach to two series of skeletally diverse 4*H*-1,3-oxazines (tetracyclic alkaloid-like 4*H*-1,3-oxazines **3** and 5-heteryl-4*H*-1,3-oxazines **4**) via a hetero-Diels–Alder reaction of 4-acyl-1*H*-pyrrole-2,3-diones fused at [*e*]-side **1** with cyanamides **2**. Tetracyclic alkaloid-like 4*H*-1,3-oxazines **3** were achieved through [4 + 2] cycloaddition of cyanamides **2** to oxa-diene system of 4-acyl-1*H*-pyrrole-2,3-diones fused at [*e*]-side **1**. 5-Heteryl-4*H*-1,3-oxazines **4** were formed as the result of thermal decomposition of tetracyclic alkaloid-like 4*H*-1,3-oxazines **3**, which proceeded via three steps (retro-Diels–Alder reaction that afforded 4-acyl-1*H*-pyrrole-2,3-diones fused at [*e*]-side **1** and cyanamides **2**; thermolytical decarbonylation of 4-acyl-1*H*-pyrrole-2,3-diones fused at [*e*]-side **1** that resulted in formation of highly reactive acyl(imidoyl)ketenes **C**; [4 + 2] cycloaddition of acyl(imidoyl)ketenes **C** as oxa-dienes with cyanamides **2** that produced 5-heteryl-4*H*-1,3-oxazines **4**).

## Figures and Tables

**Table 1:** Cycloaddition reaction of FPDs **1a–i** with cyanamides **2a–f**.

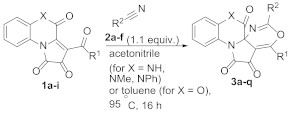						
Entry	FPD 1	Cyanamide 2	X	R^1^	R^2^	Yield ^1^ of 3, %
**3a**	**1a**	**2a**	NPh	Ph	NEt_2_	85
**3b**	**1a**	**2b**	NPh	Ph	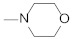	89
**3c**	**1a**	**2c**	NPh	Ph	NMe_2_	78
**3d**	**1a**	**2d**	NPh	Ph	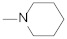	83
**3e**	**1b**	**2b**	NPh	4-ClC_6_H_4_	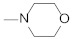	88
**3f**	**1c**	**2b**	NPh	4-MeOC_6_H_4_	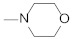	91
**3g**	**1d**	**2c**	NPh	4-NO_2_C_6_H_4_	NMe_2_	81
**3h**	**1e**	**2b**	NMe	4-MeC_6_H_4_	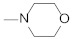	86
**3i**	**1f**	**2b**	NPh	*t*-Bu	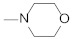	79
**3j**	**1g**	**2b**	NH	Ph	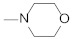	92
**3k**	**1h**	**2b**	NPh	MeO	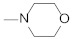	0^2^
**3l**	**1i**	**2b**	O	Ph	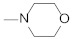	81
**3m**	**1i**	**2c**	O	Ph	NMe_2_	74
**3n**	**1d**	**2b**	NPh	4-NO_2_C_6_H_4_	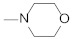	Traces ^2^
**3o**	**1a**	**2e**	NPh	Ph	NHC_6_H_4_Cl-4	Traces ^2^
**3p**	**1a**	**2f**	NPh	Ph	NHC_6_H_4_OMe-4	Traces ^2^
**3q**	**1l**	**2e**	O	Ph	NHC_6_H_4_Cl-4	Traces ^2^

^1^ Isolated yields (reaction scale of 0.76 mmol). ^2^ According to UPLC-UV-MS.

**Table 2:** Thermal decomposition of compounds **3a–j,l,m**.

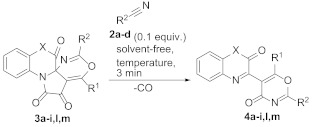							
Entry	Precursor 3	Cyanamide 2	X	R^1^	R^2^	Temperature ^1^, °C	Yield ^2^ of 4, %
**4a**	**3a**	**2a**	NPh	Ph	NEt_2_	215–220	71
**4b**	**3b**	**2b**	NPh	Ph	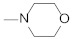	235–240	85
**4c**	**3c**	**2c**	NPh	Ph	NMe_2_	230–235	78
**4d**	**3d**	**2d**	NPh	Ph	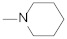	230–235	77
**4e**	**3e**	**2b**	NPh	C_6_H_4_Cl-4	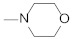	240–245	86
**4f**	**3f**	**2b**	NPh	C_6_H_4_OMe-4	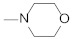	250–255	91
**4g**	**3g**	**2c**	NPh	C_6_H_4_NO_2_-4	NMe_2_	220–225	79
**4h**	**3h**	**2b**	NMe	C_6_H_4_Me-4	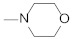	220–225	82
**4i**	**3i**	**2b**	NPh	Bu-*t*	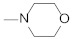	190–195	84
**4j**	**3j**	**2b**	NH	Ph	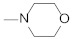	210–215	0 ^3^
**4l**	**3l**	**2b**	O	Ph	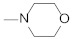	230–235	65
**4m**	**3m**	**2c**	O	Ph	NMe_2_	240–245	68

^1^ Bath temperature. ^2^ Isolated yields (reaction scale of 0.4 mmol). ^3^ According to UPLC-UV-MS.

## Data Availability

The presented data are available in this article.

## Supplementary Materials

The following supporting information can be downloaded at: https://www.mdpi.com/article/10.3390/molecules27165257/s1, Copies of NMR spectra for all new compounds, ORTEP images of X-ray crystal structures.

## Sample Availability

Samples of the all compounds are available from the authors.
