# Supplementary material for: Cycloaddition of 4-Acyl-1H-pyrrole-2,3-diones Fused at [e]-Side and Cyanamides: Divergent Approach to 4H-1,3-Oxazines
Source: Molecules. 2022 Aug 17;27(16):5257. doi: 10.3390/molecules27165257 (PMC9414543; doi:10.3390/molecules27165257)
Supplement: Supplementary file 1 [file molecules-27-05257-s001.zip › Experimental_SI.pdf]

Supplementary Materials

for

Hetero-Diels–Alder Reaction of 4-Acyl-1*H*-pyrrole-2,3-diones Fused at [*e*]-Side and Cyanamides: Divergent Approach to 4*H*-1,3-Oxazines

Ekaterina E. Khramtsova *, Aleksandr D. Krainov, Maksim V. Dmitriev, Andrey N. Maslivets

Department of Chemistry

Perm State University

ul. Bukireva 15, 614990 Perm, Russia

* E-mail: caterina.stepanova@psu.ru

Table of contents

NMR charts of compounds **3**……………………………………………………….S3
NMR charts of compounds **4**……….……………………………….……………...S27
NMR charts of compound **I**………….………………………….………………...S49
ORTEP images of X-ray crystal structures……..…………….…………….…...S51

NMR charts of compounds 3

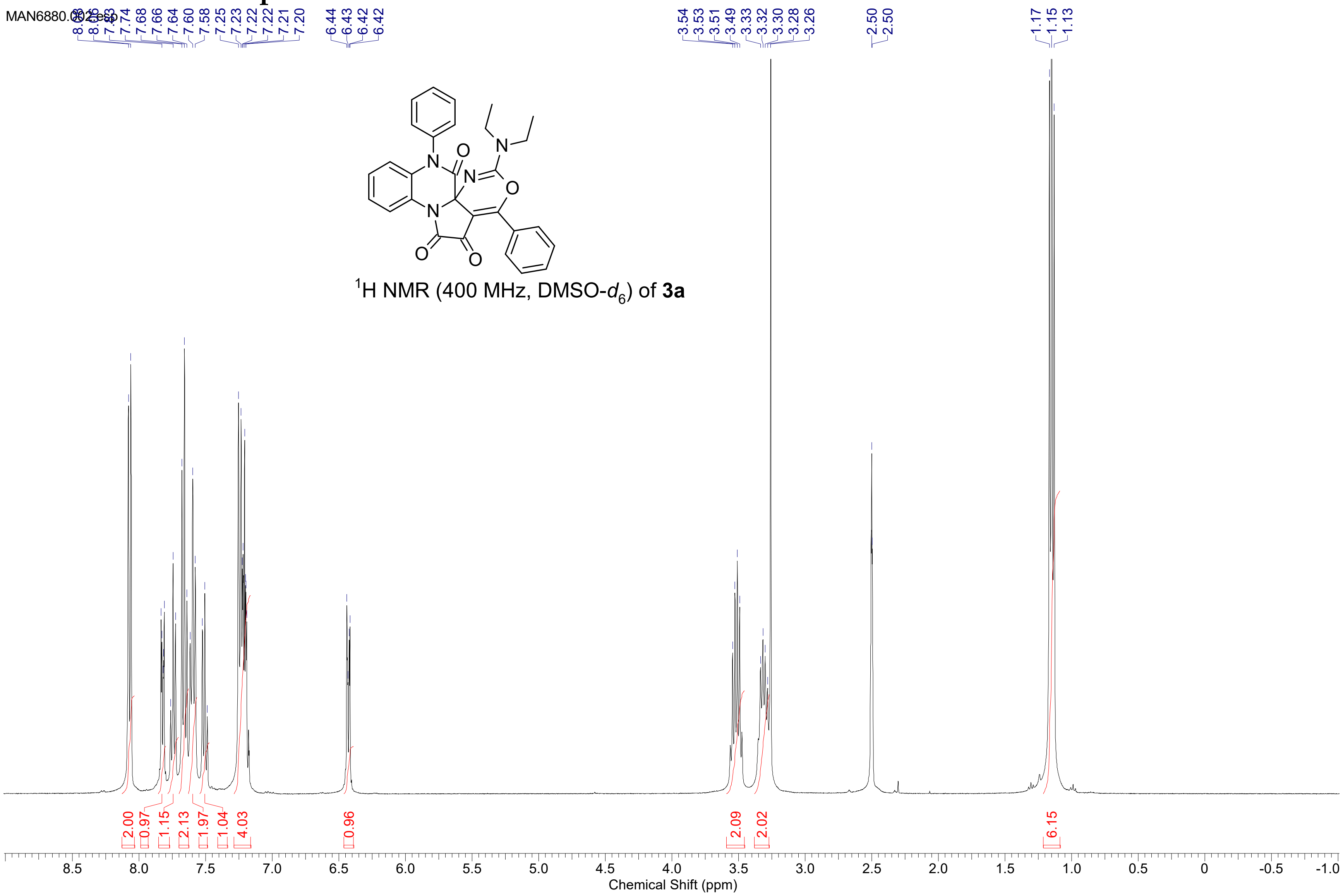

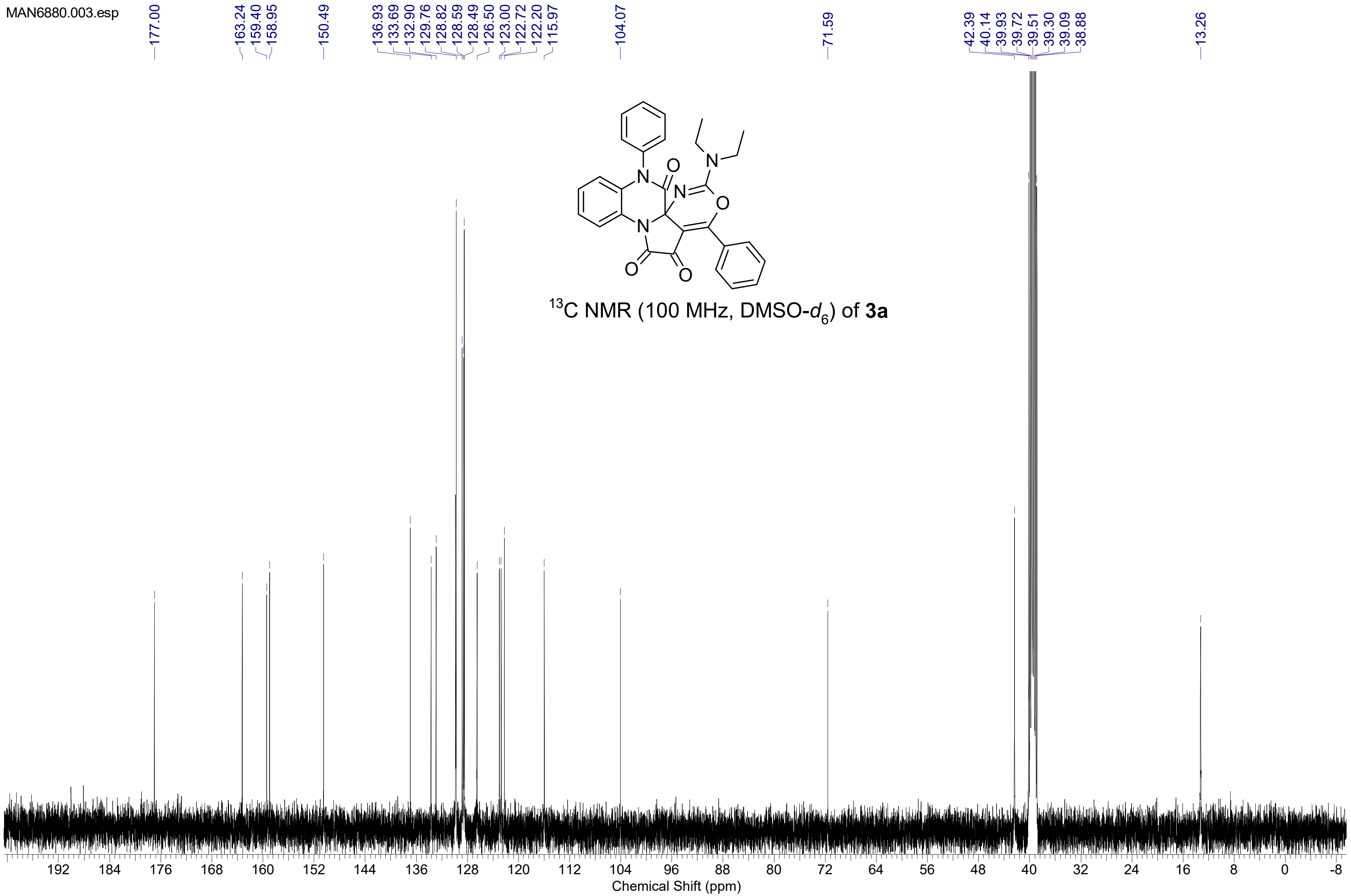

^{13}C NMR (100 MHz, DMSO-d_6) of **3a**

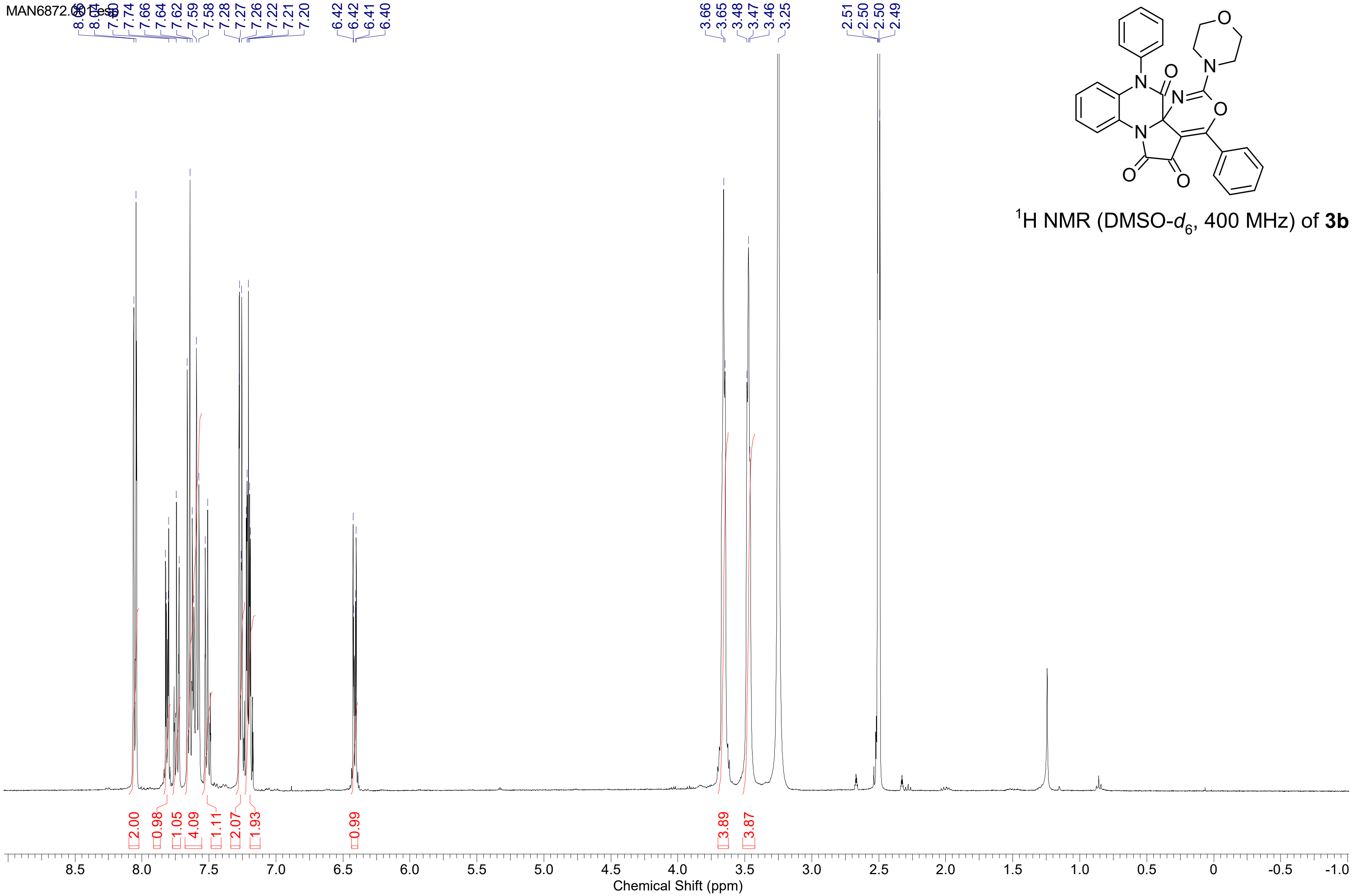

^{1}H NMR (DMSO-d_6, 400 MHz) of **3b**

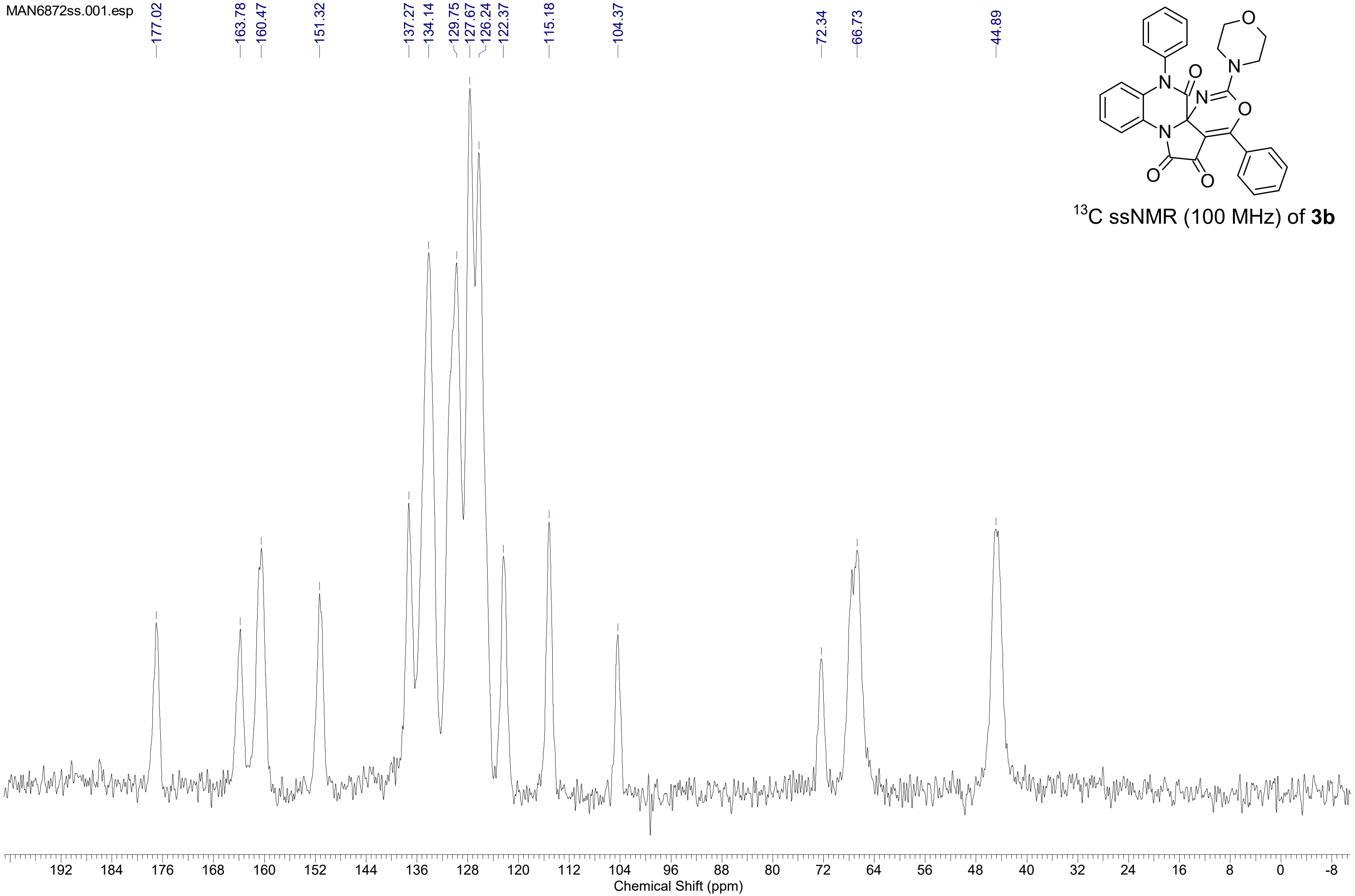

^{13}C ssNMR (100 MHz) of **3b**

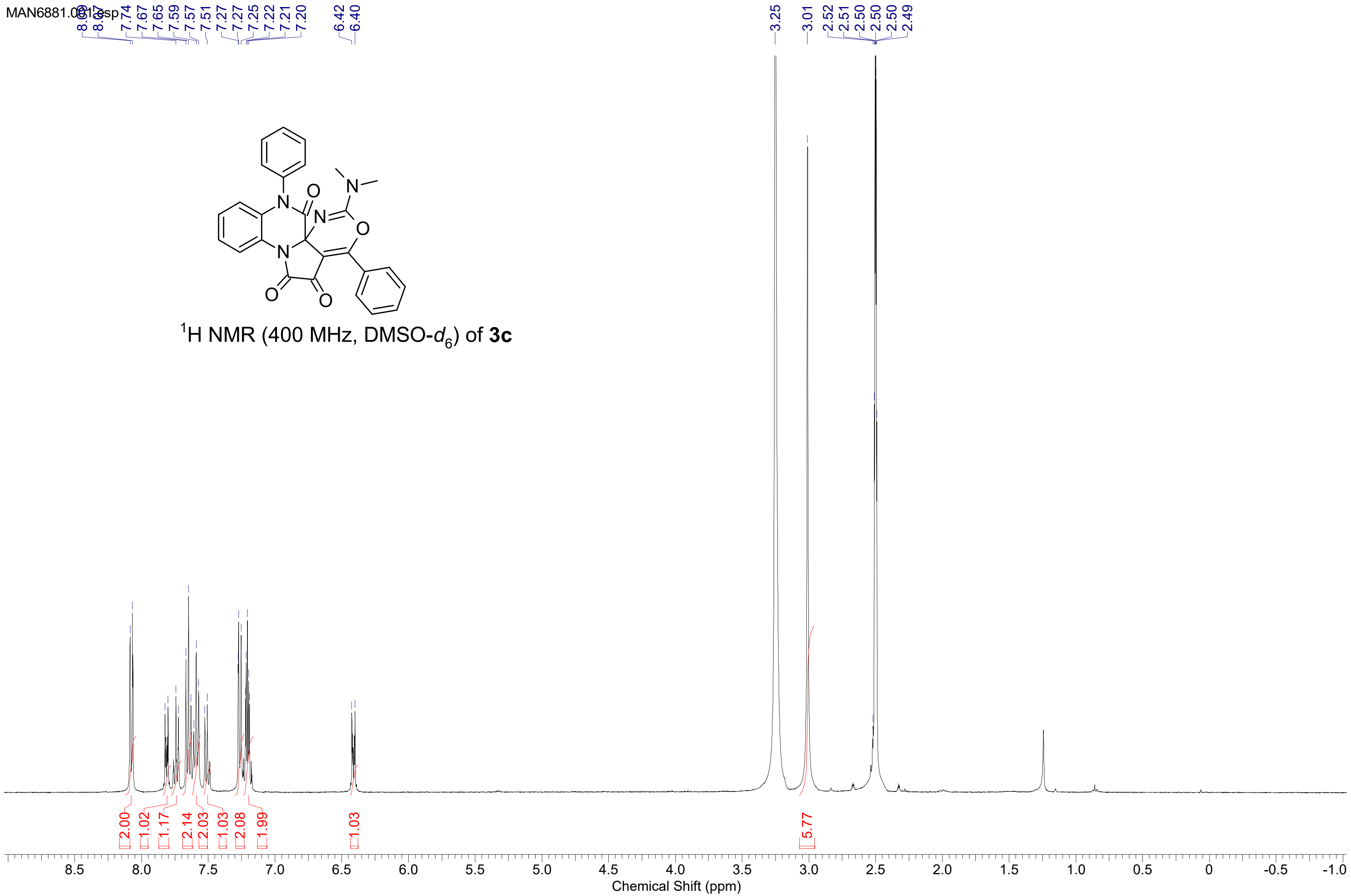

^{1}H NMR (400 MHz, DMSO-d_6) of **3c**

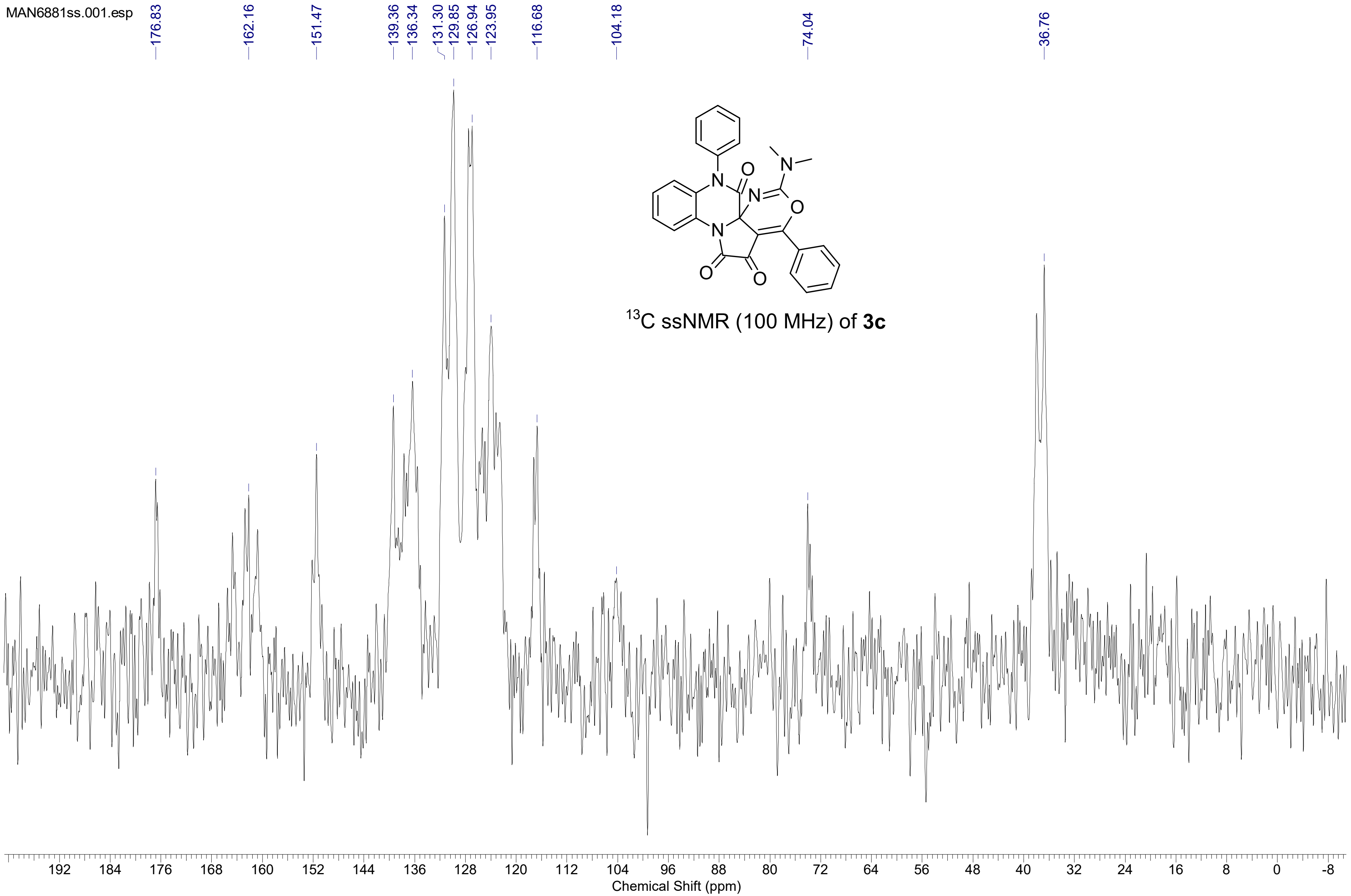

^{13}C ssNMR (100 MHz) of **3c**

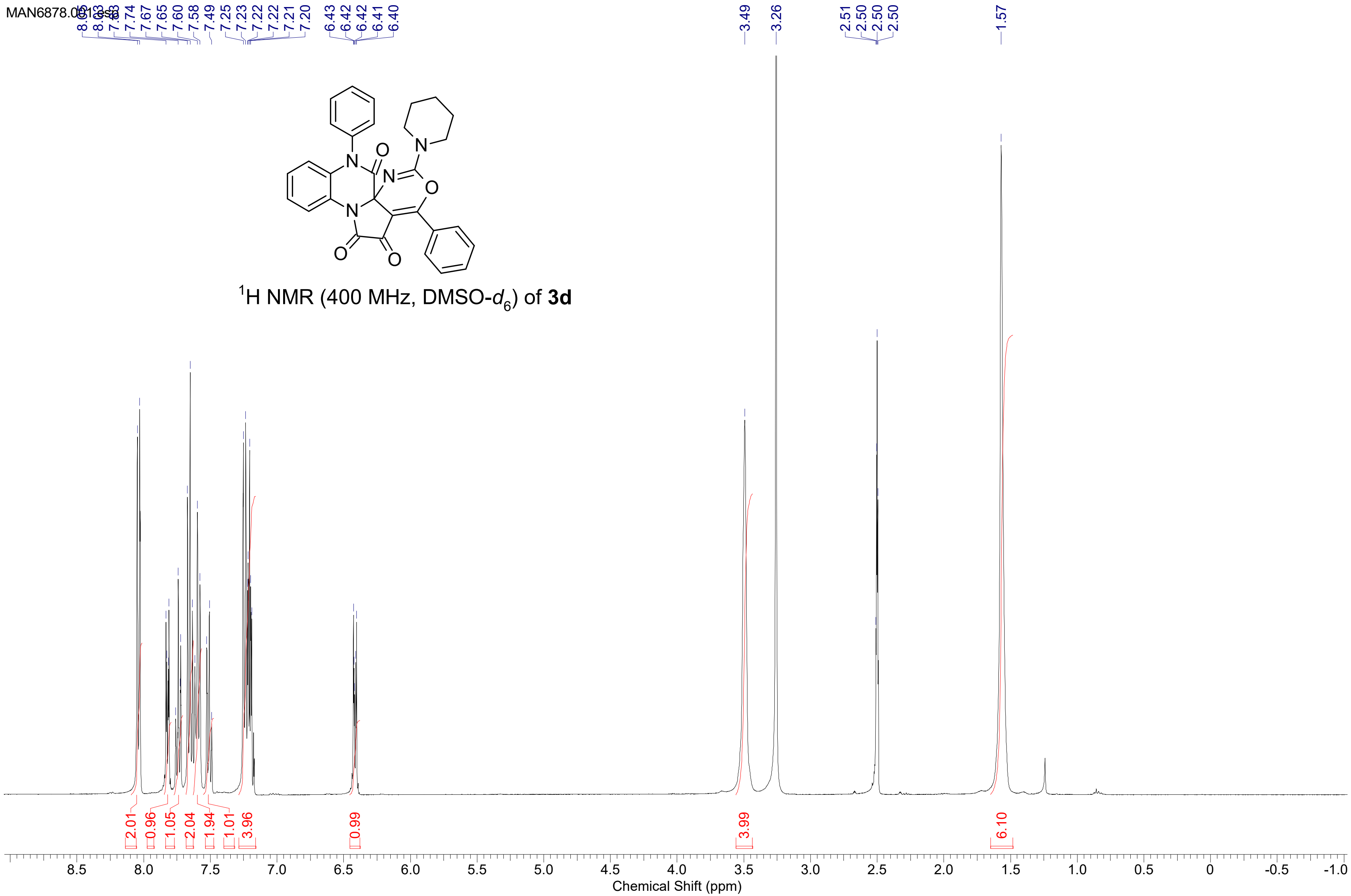

^{1}H NMR (400 MHz, DMSO-d_6) of **3d**

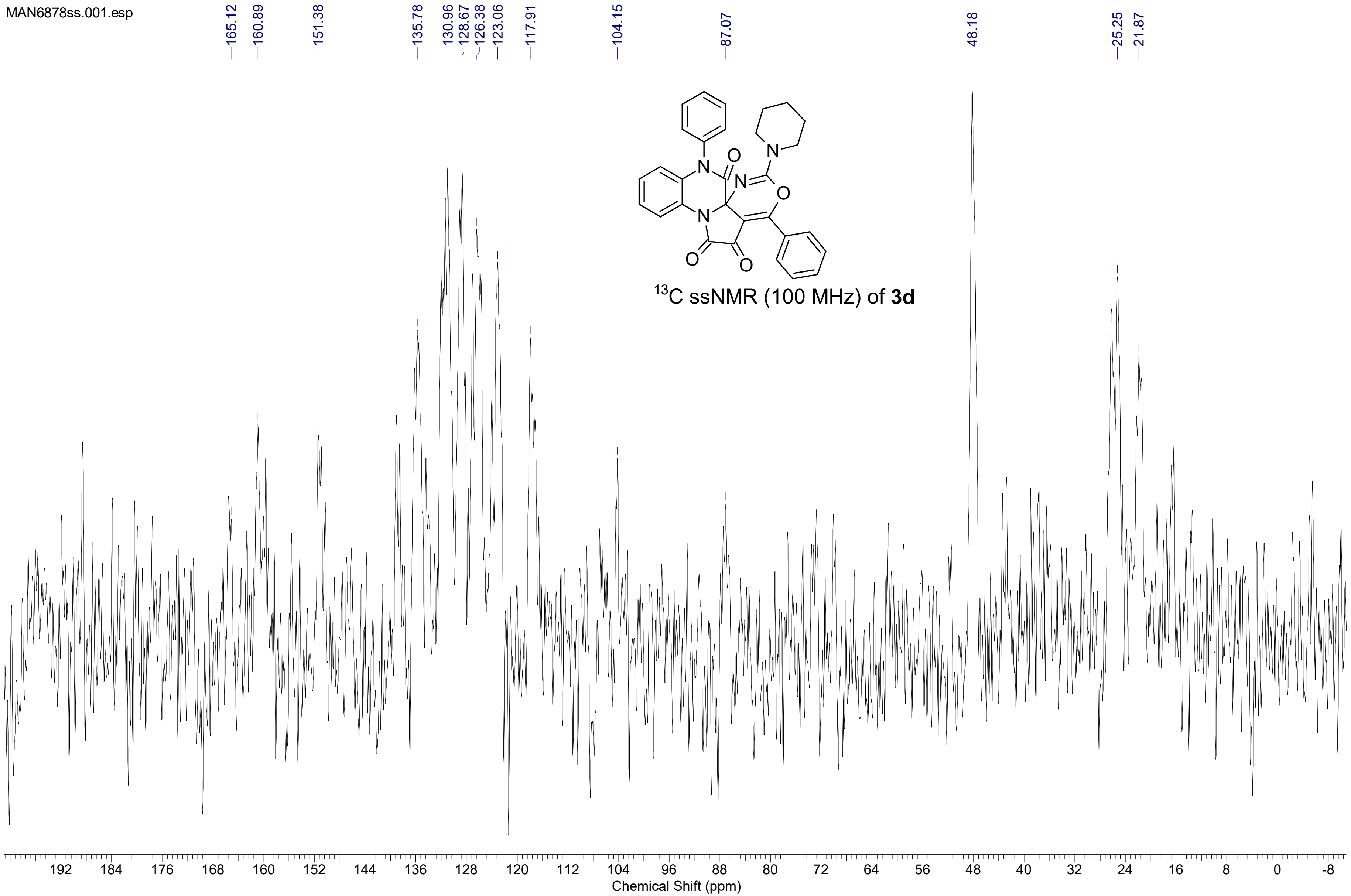

^{13}C ssNMR (100 MHz) of **3d**

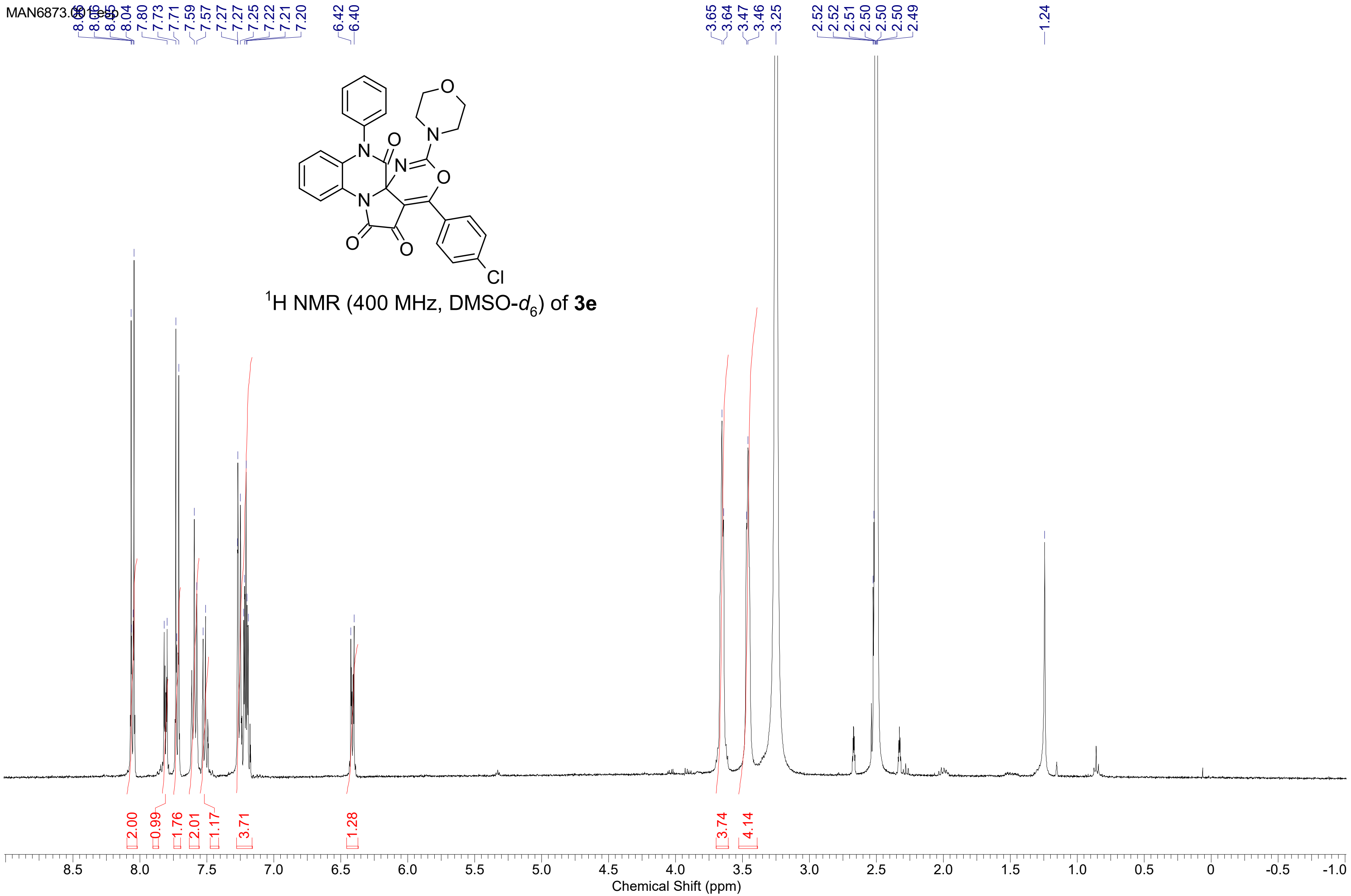

^{1}H NMR (400 MHz, DMSO-d_6) of **3e**

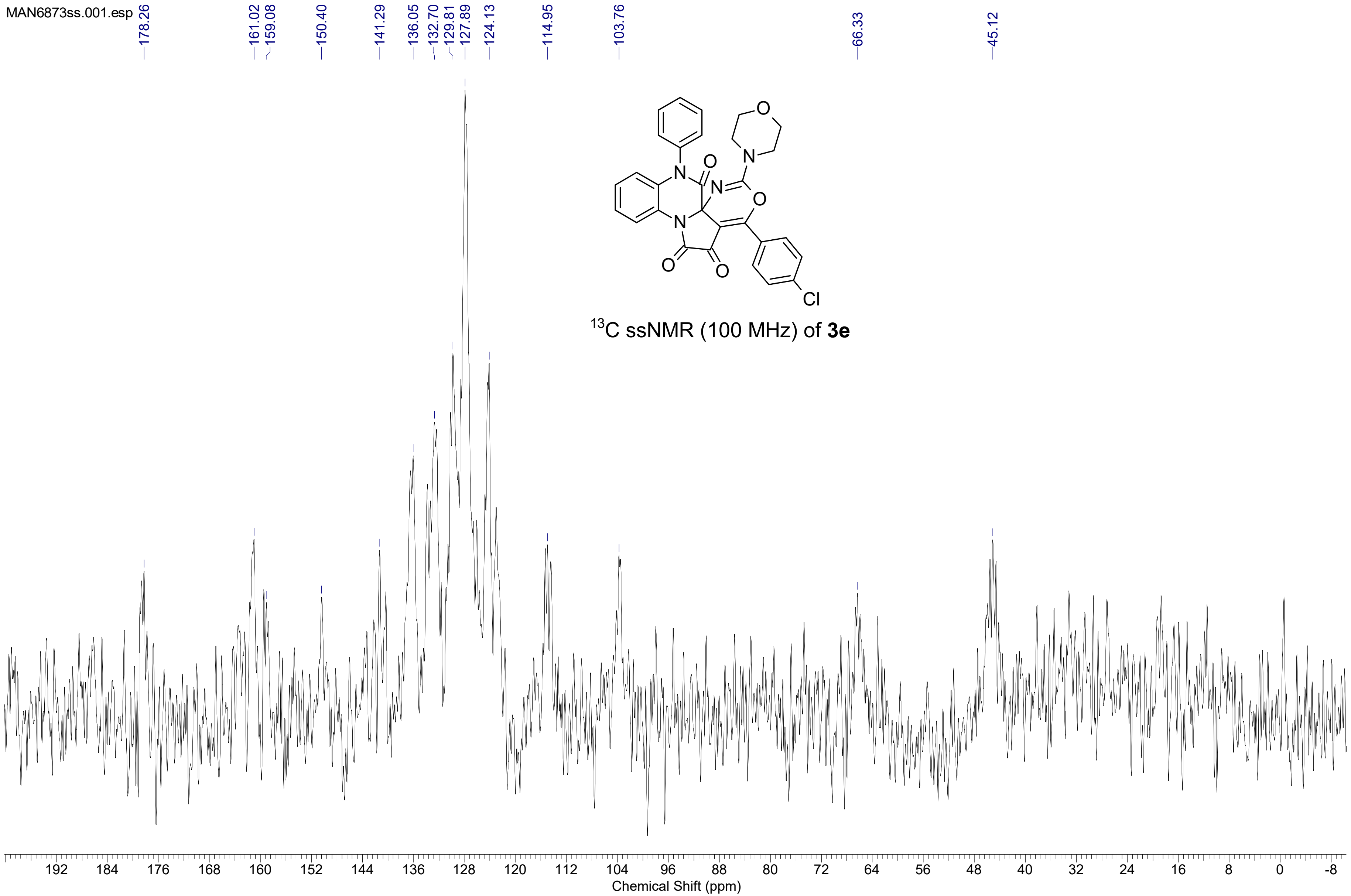
MAN6873ss.001.esp
178.26
161.02
159.08
150.40
141.29
136.05
132.70
129.81
127.89
124.13
114.95
103.76
66.33
45.12
O
N
N
O
N
O
N
O
O
O
Cl
13C ssNMR (100 MHz) of 3e
192
184
176
168
160
152
144
136
128
120
112
104
96
88
80
72
64
56
48
40
32
24
16
8
0
-8
Chemical Shift (ppm)

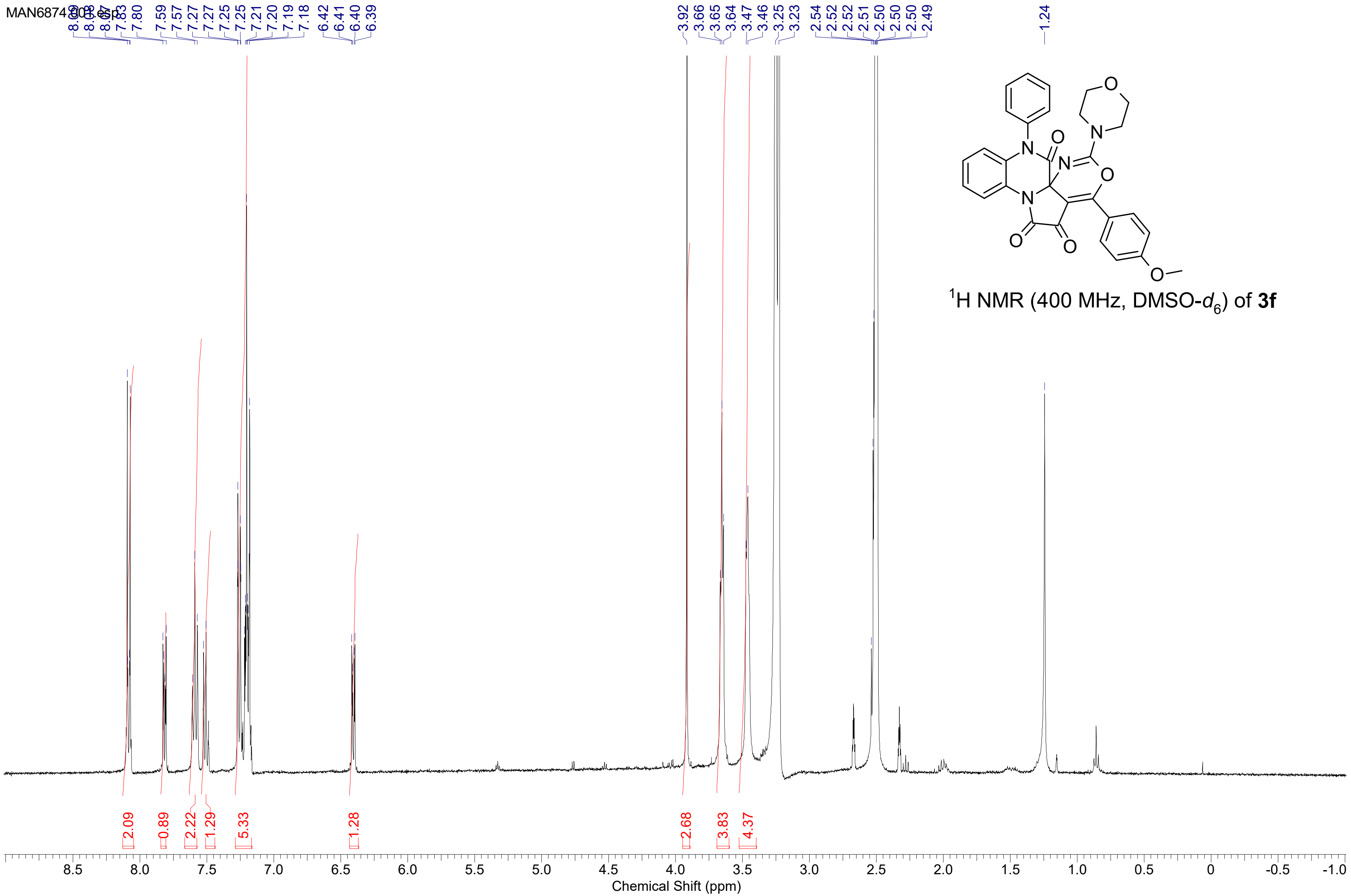

^{1}H NMR (400 MHz, DMSO-d_6) of **3f**

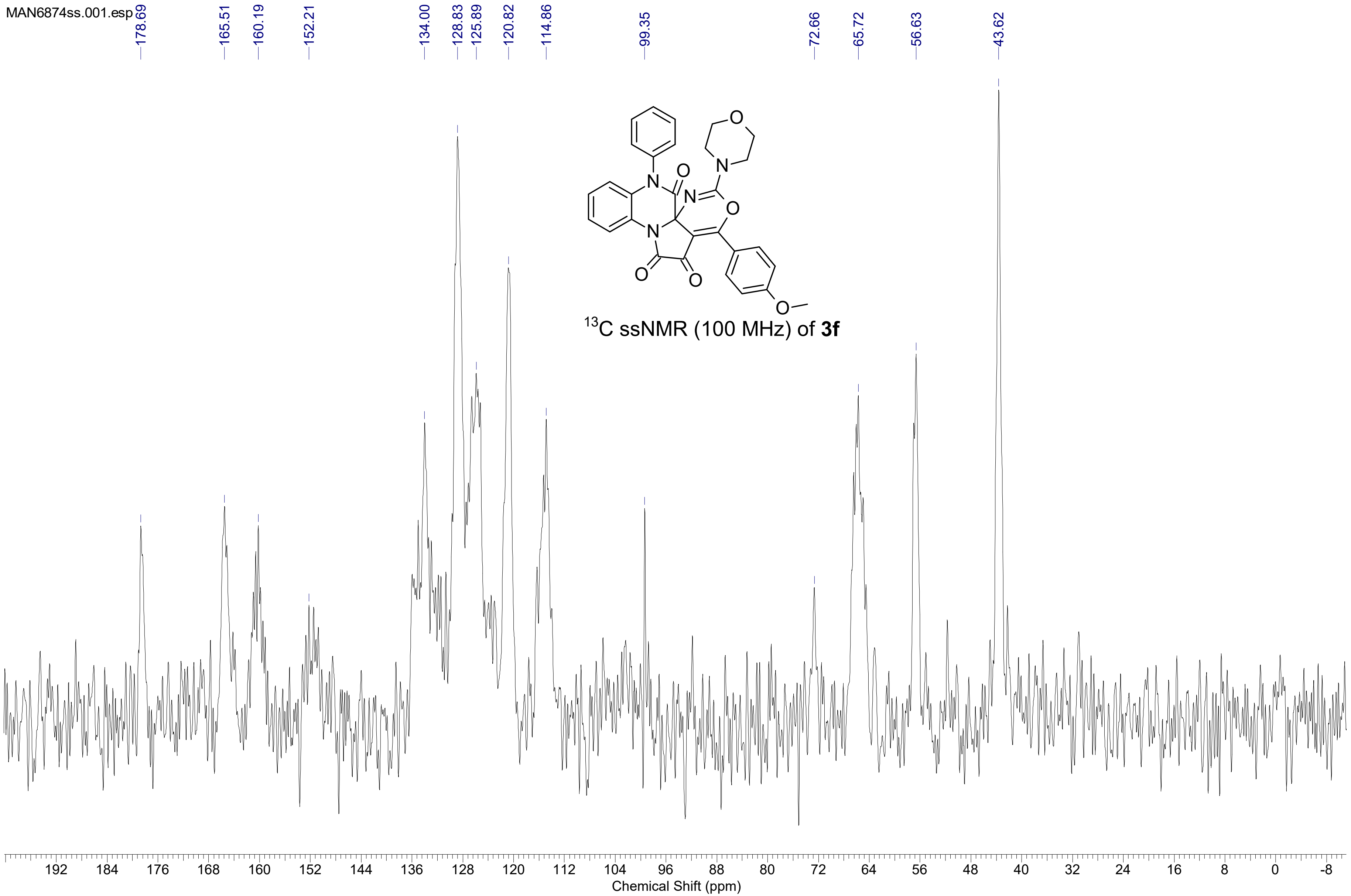

^{13}C ssNMR (100 MHz) of **3f**

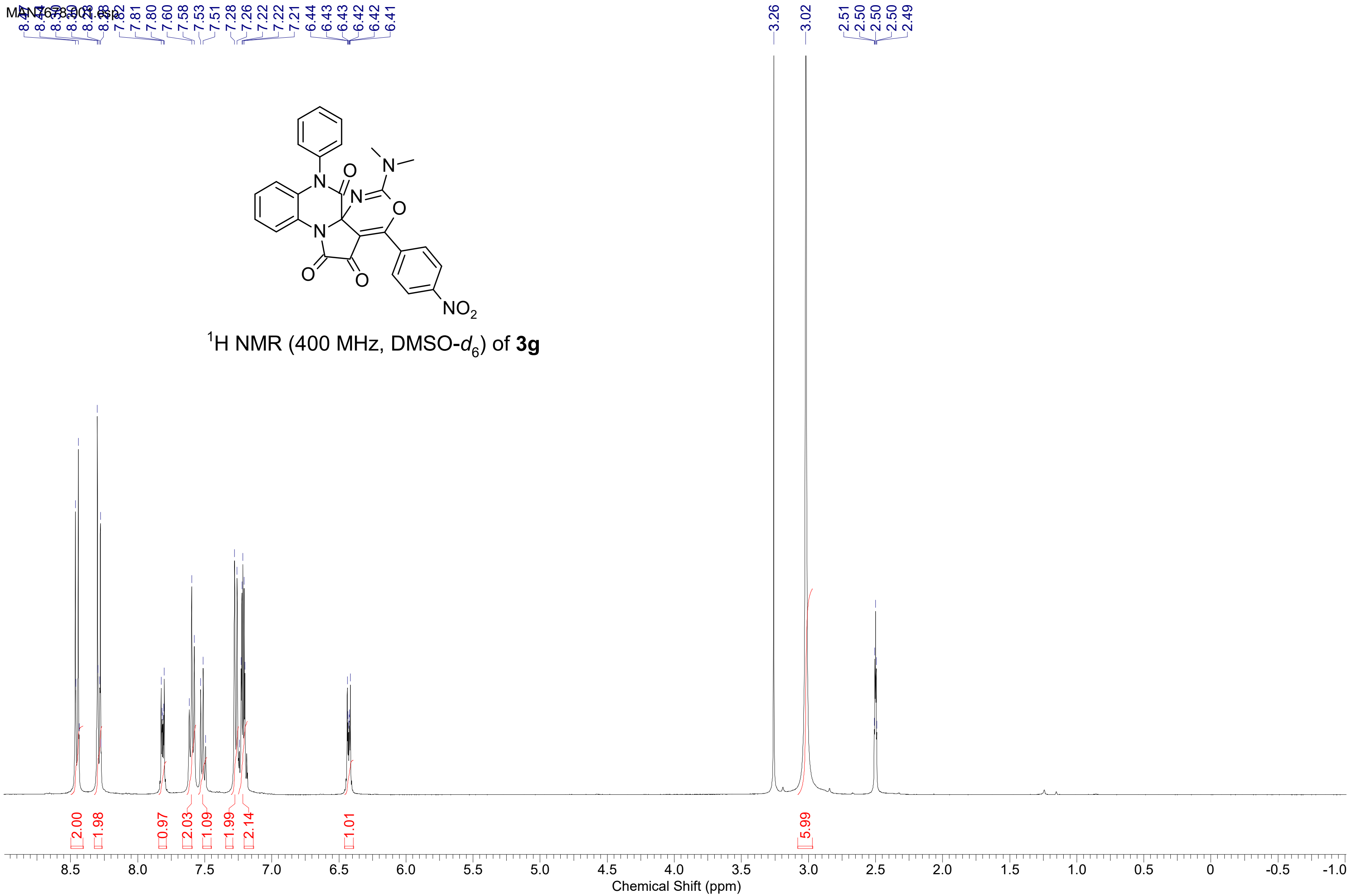

^{1}H NMR (400 MHz, DMSO-d_6) of **3g**

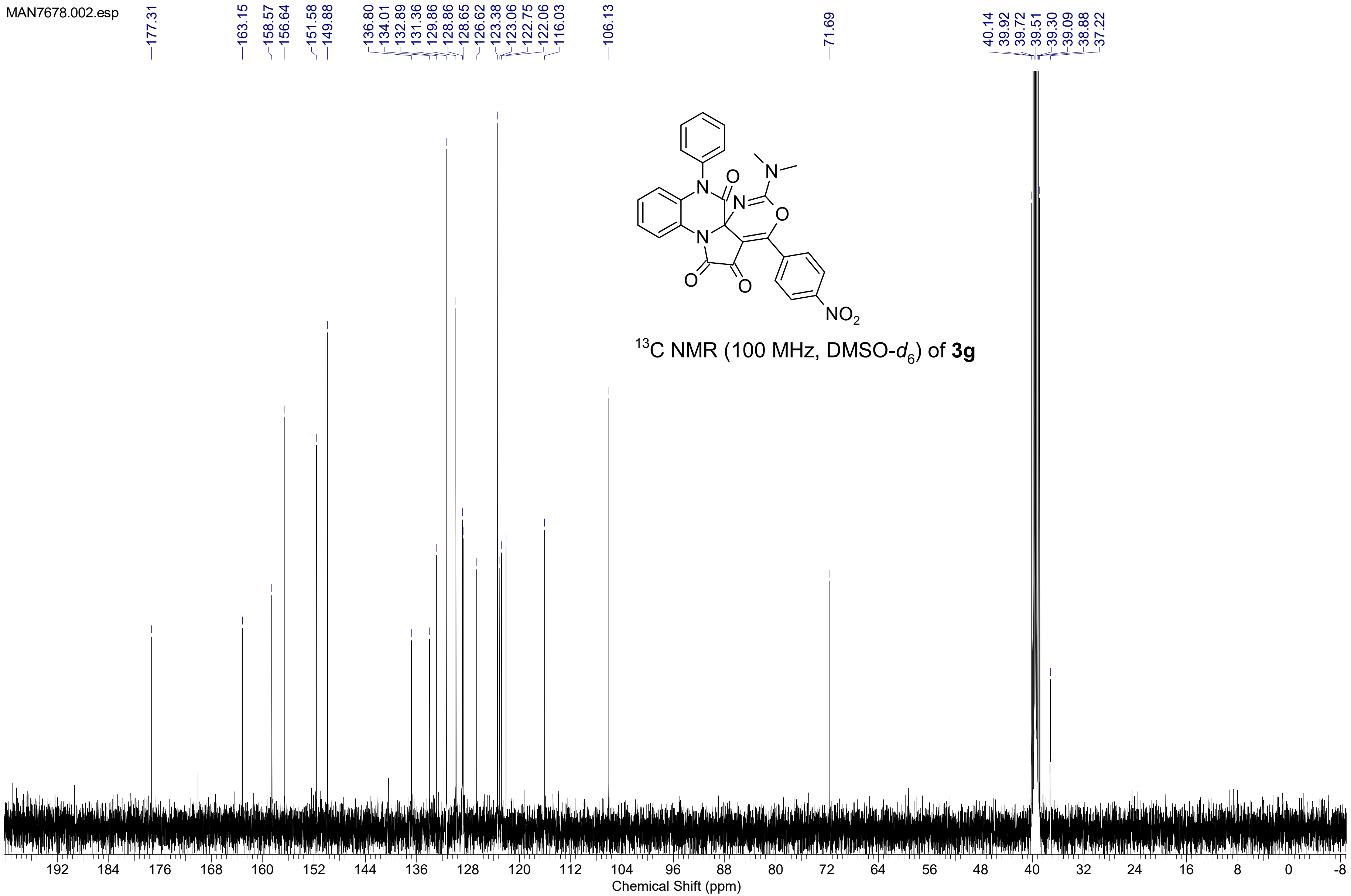

^{13}C NMR (100 MHz, DMSO-d_6) of **3g**

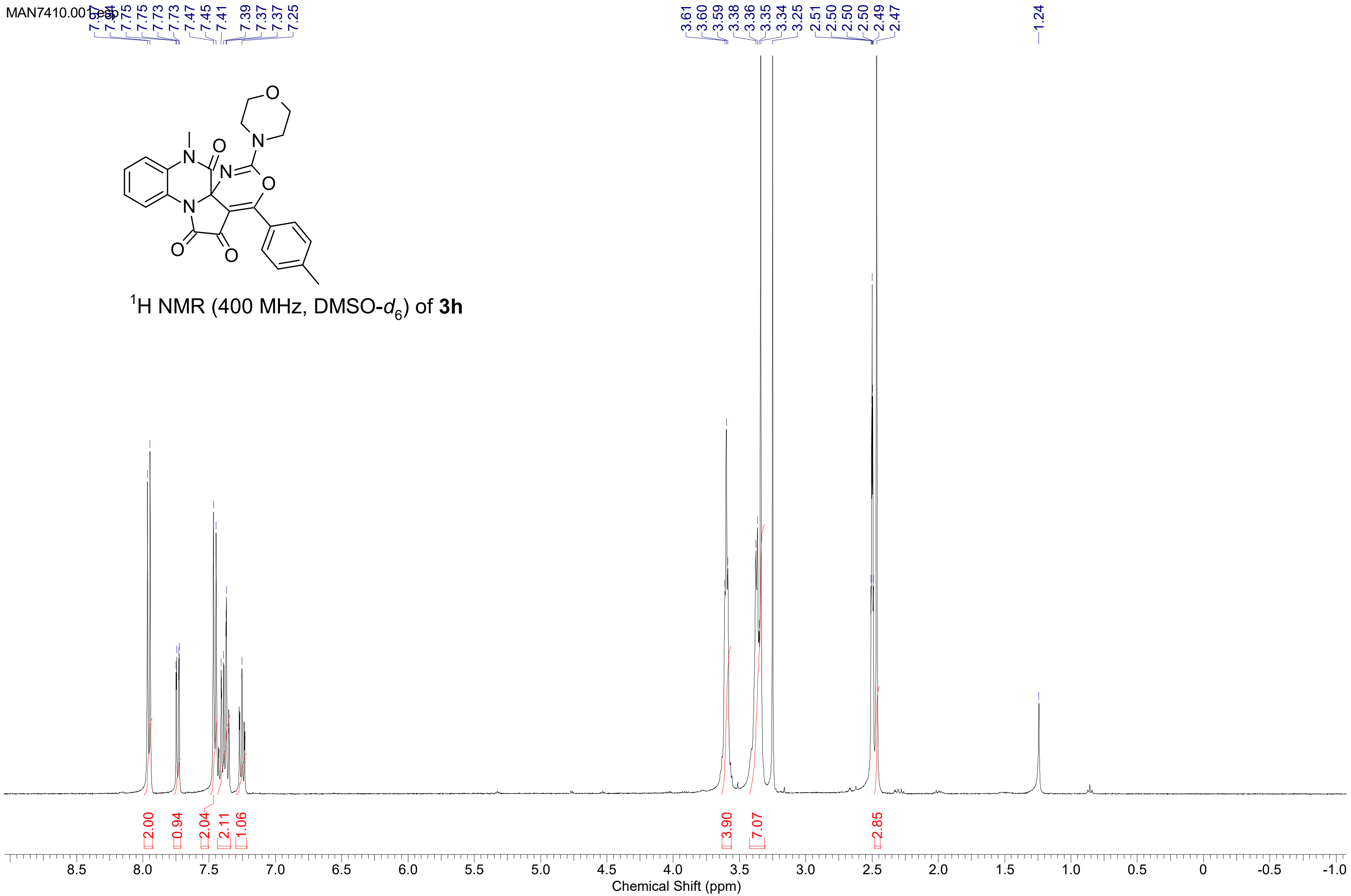

^{1}H NMR (400 MHz, DMSO-d_6) of **3h**

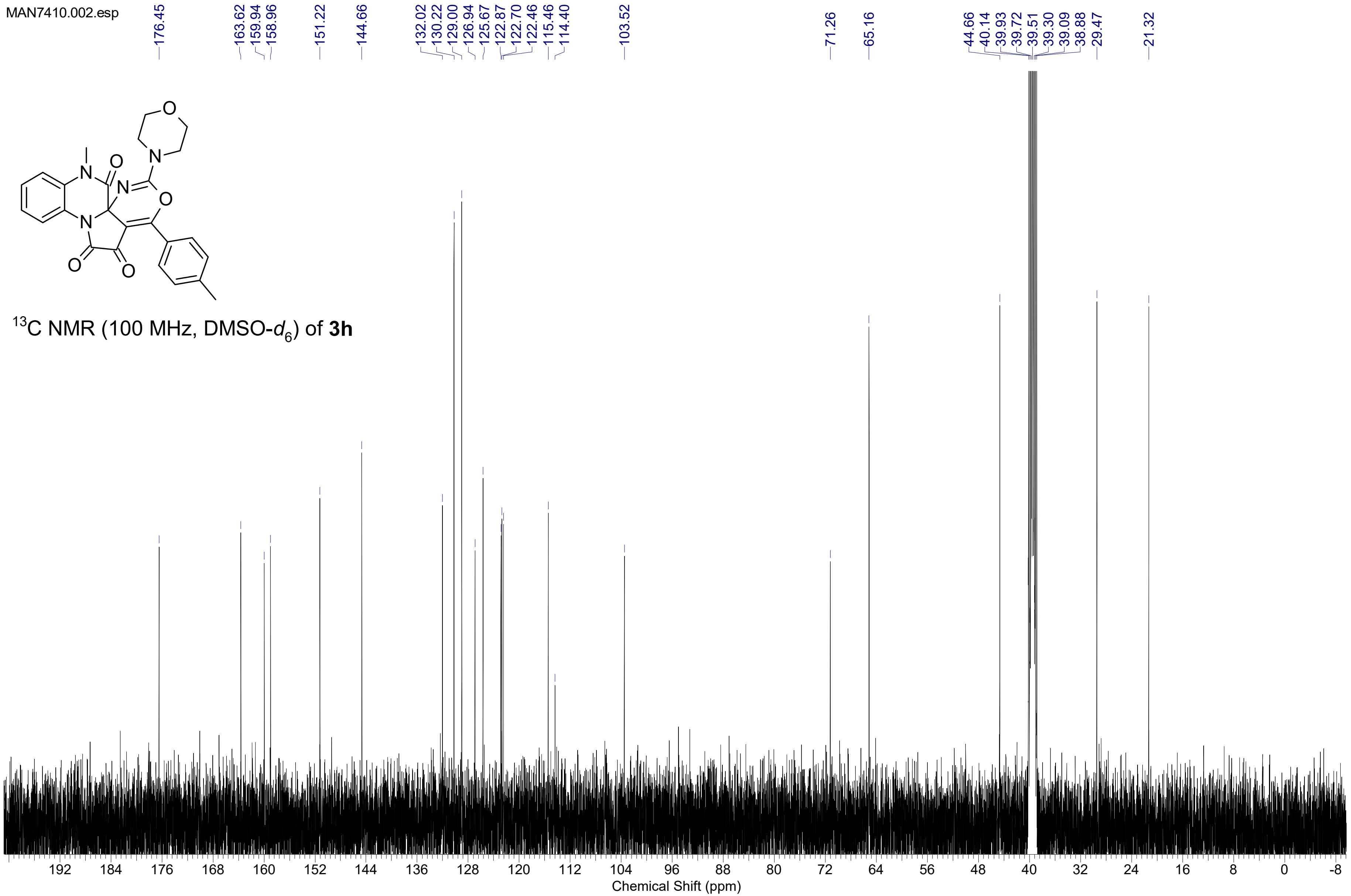

^{13}C NMR (100 MHz, DMSO-d_6) of **3h**

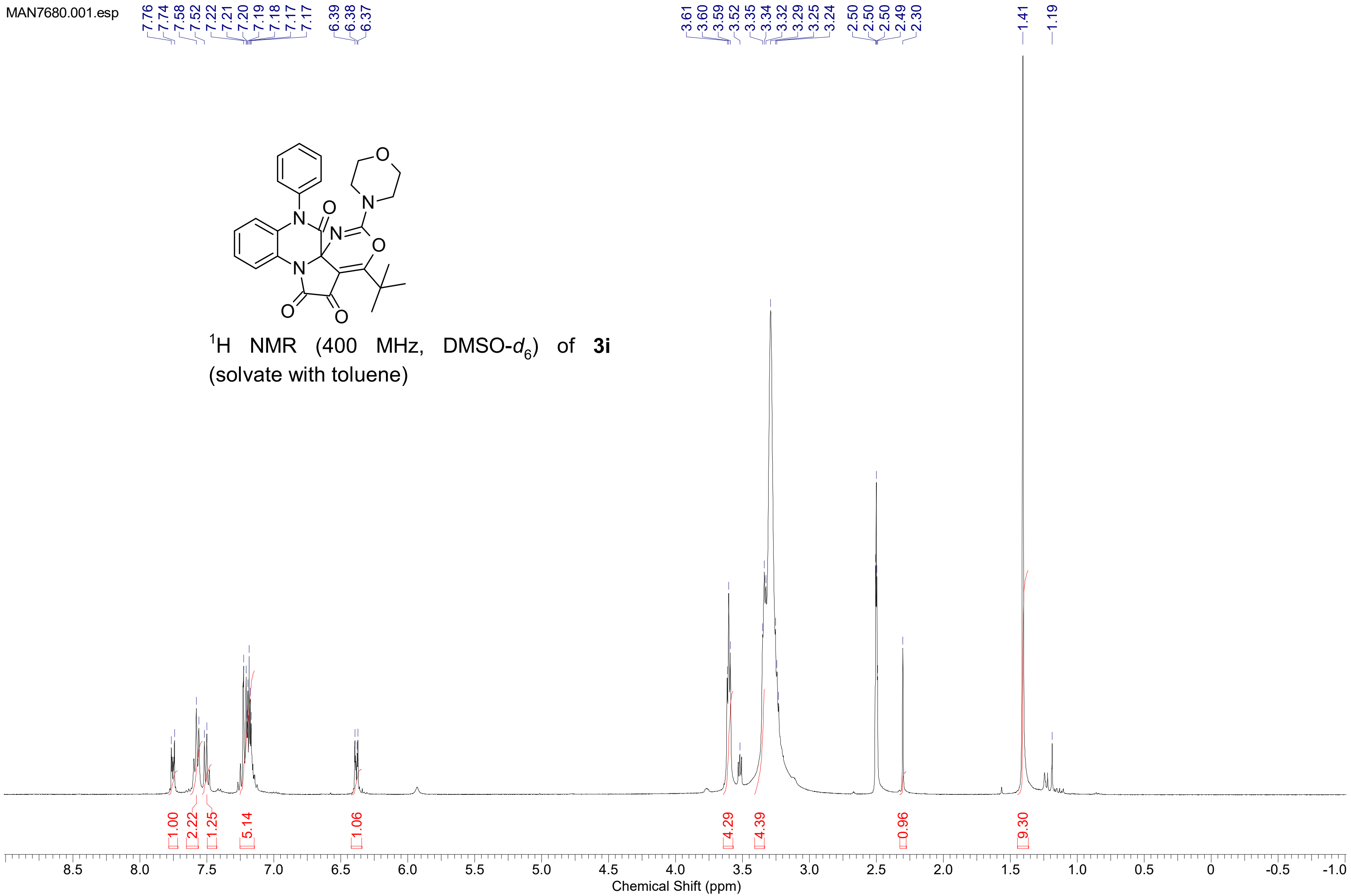

^{1}H NMR (400 MHz, DMSO-d_6) of **3i** (solvate with toluene)

MAN7680.003.esp

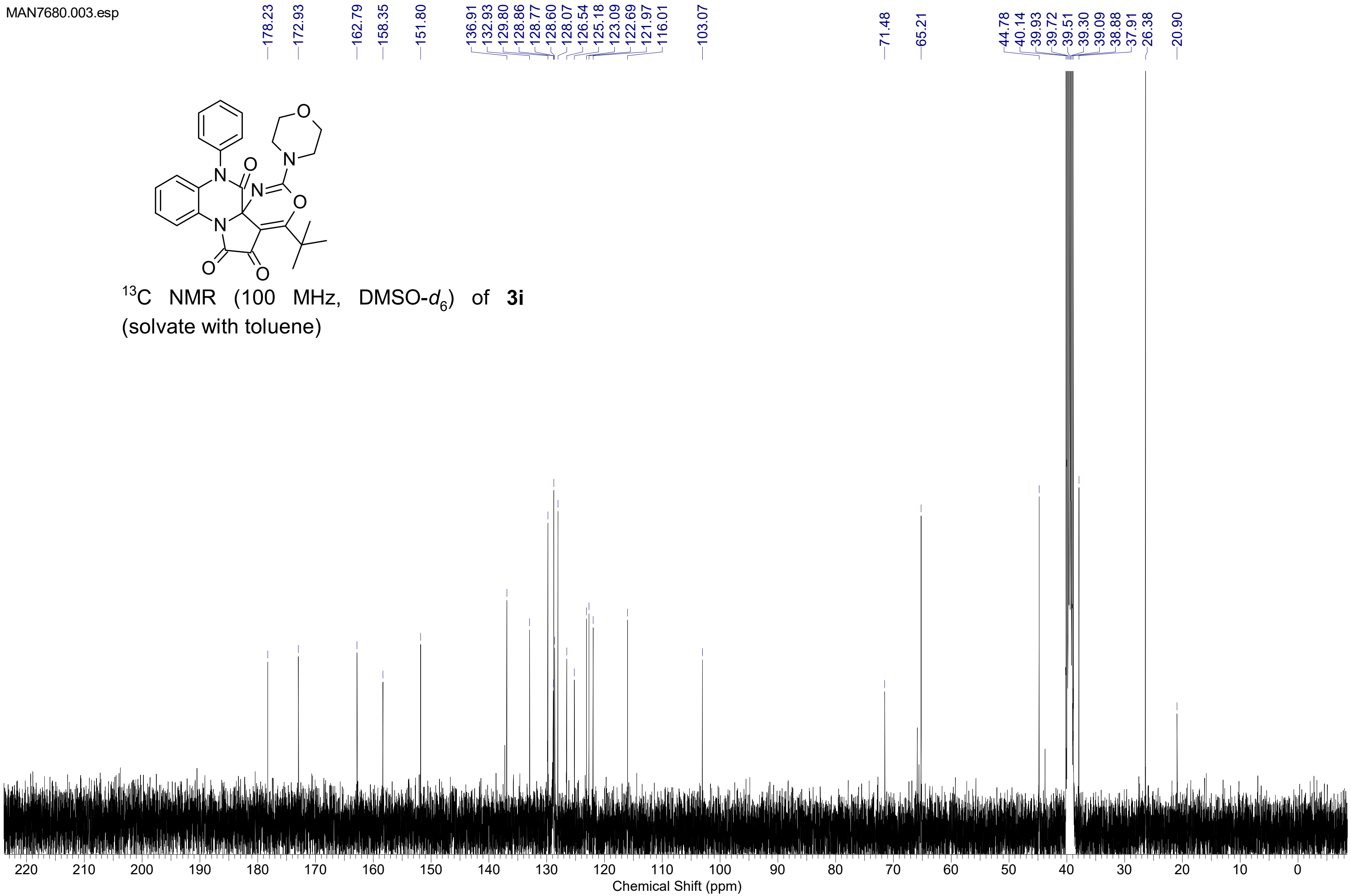

^{13}C NMR (100 MHz, DMSO-d_6) of **3i** (solvate with toluene)

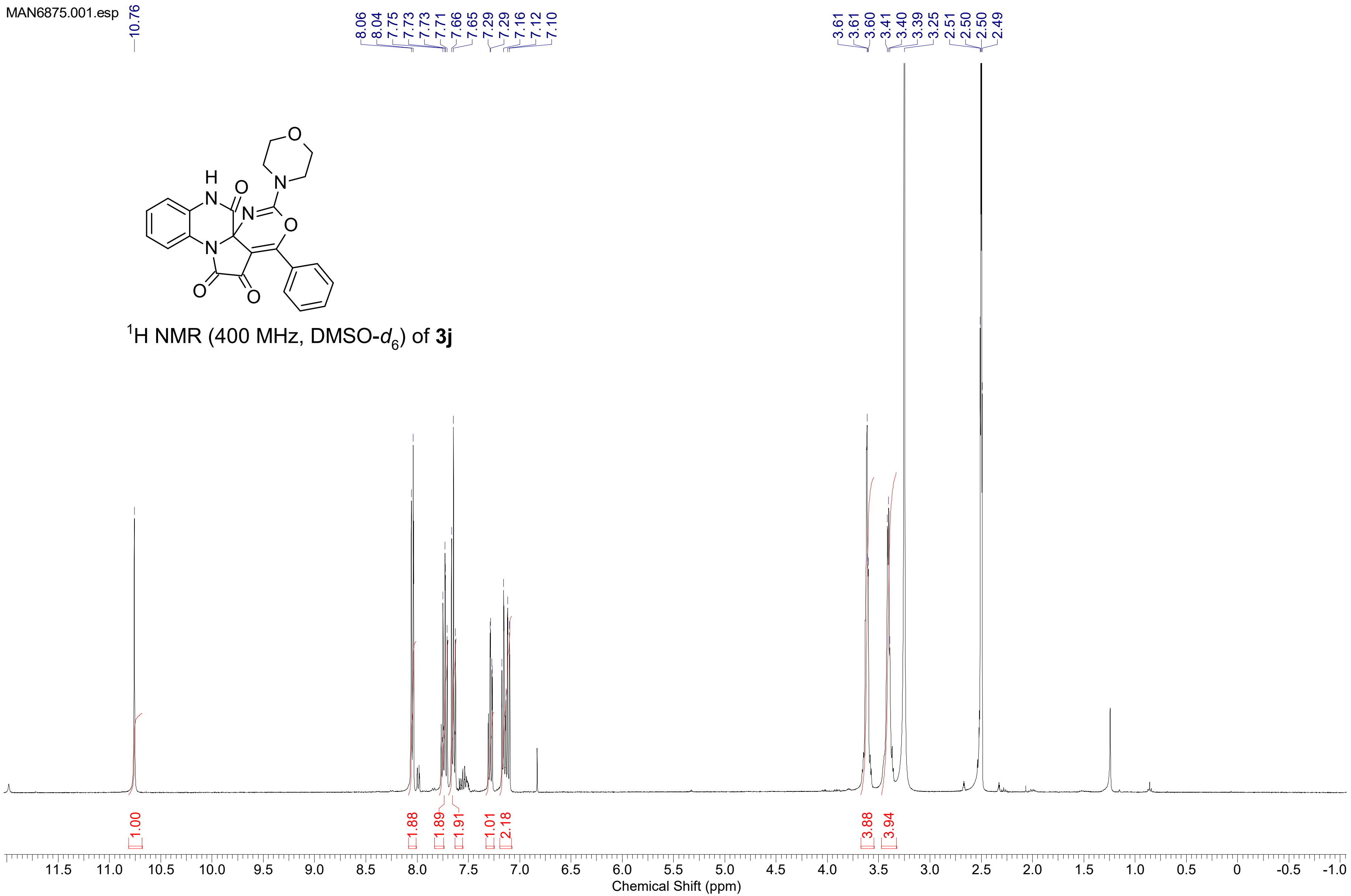

^{1}H NMR (400 MHz, DMSO-d_6) of **3j**

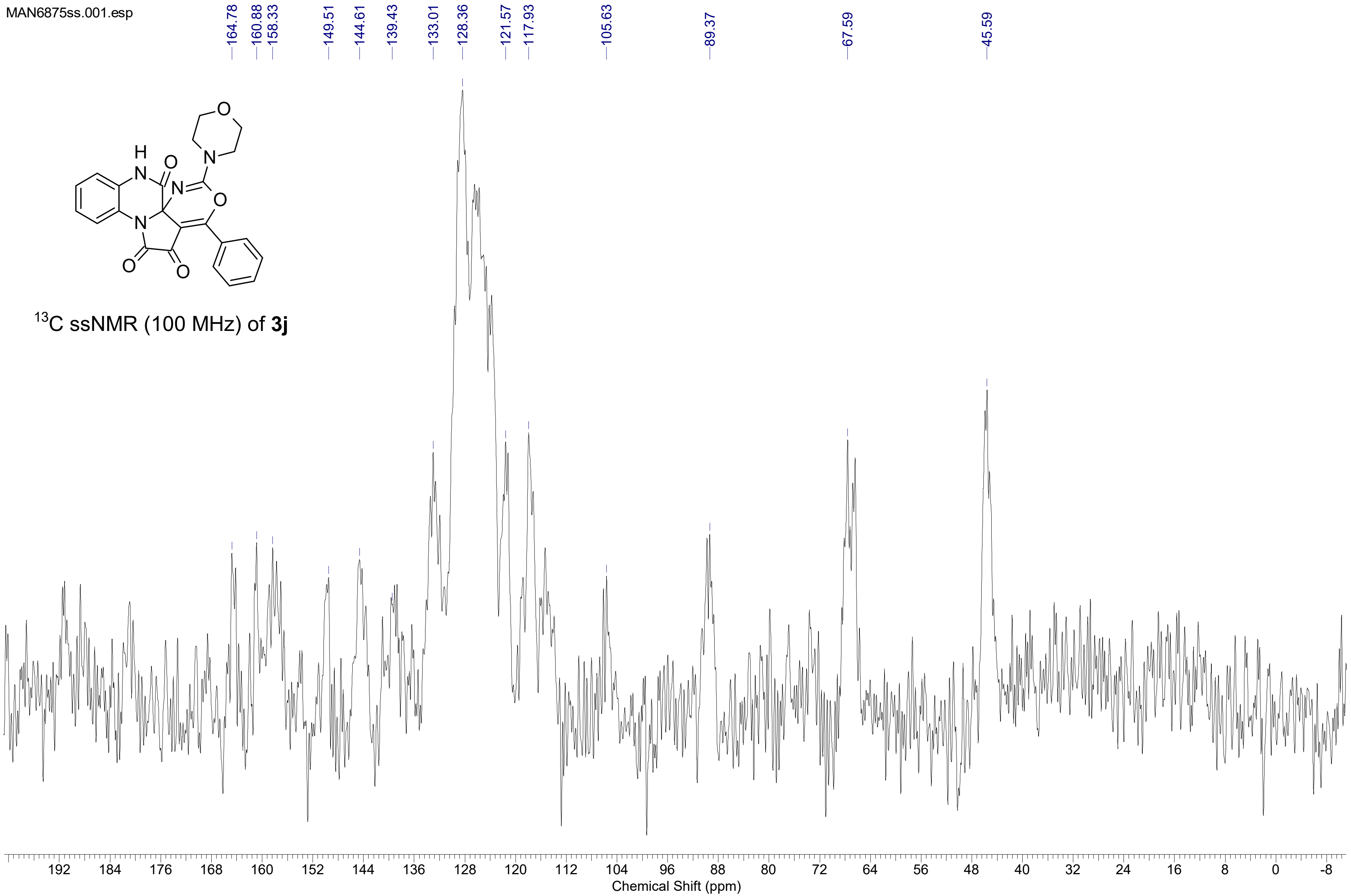
MAN6875ss.001.esp
164.78
160.88
158.33
149.51
144.61
139.43
133.01
128.36
121.57
117.93
105.63
89.37
67.59
45.59
13C ssNMR (100 MHz) of 3j
192 184 176 168 160 152 144 136 128 120 112 104 96 88 80 72 64 56 48 40 32 24 16 8 0 -8
Chemical Shift (ppm)

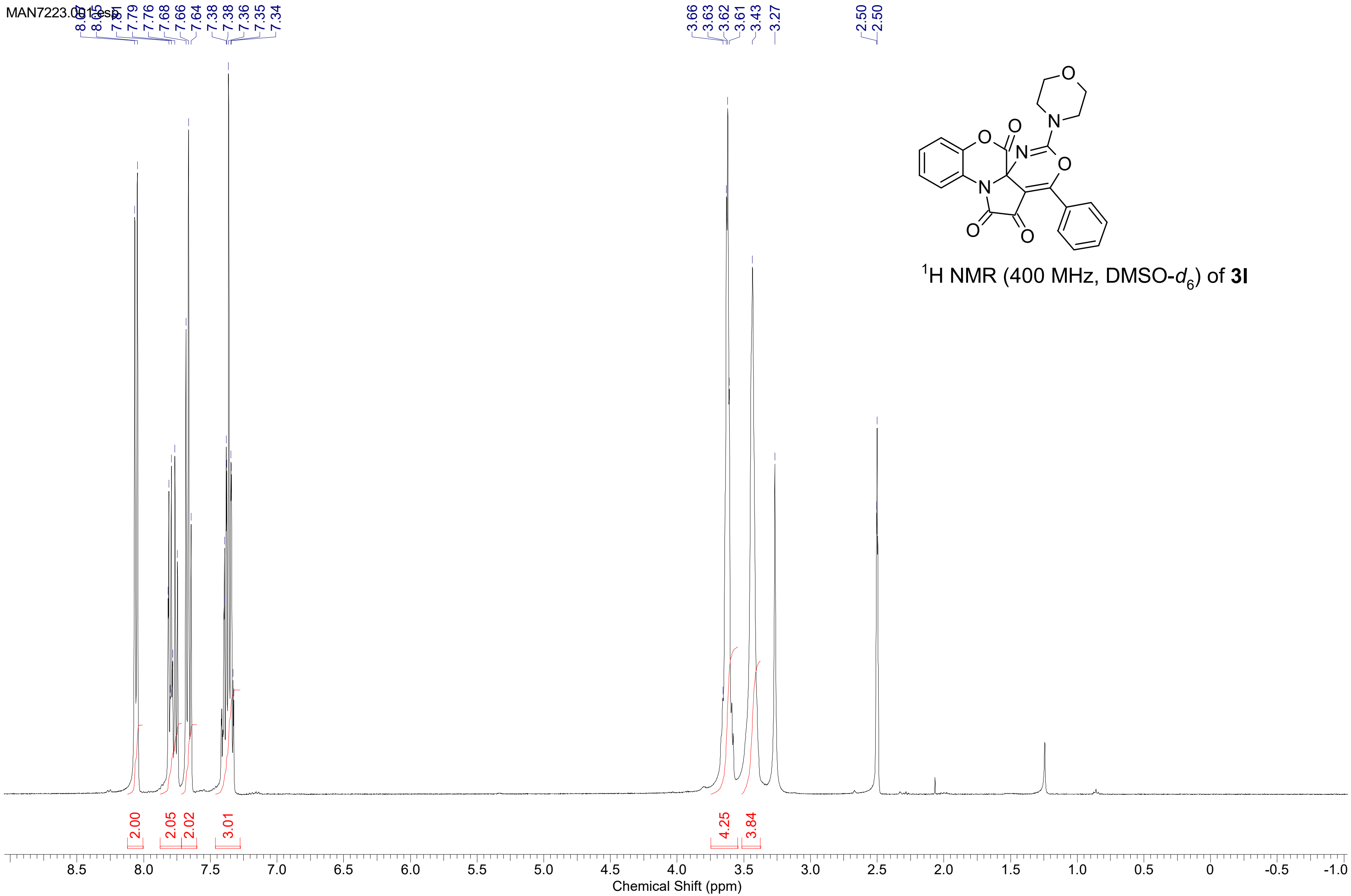

^{1}H NMR (400 MHz, DMSO-d_6) of **3l**

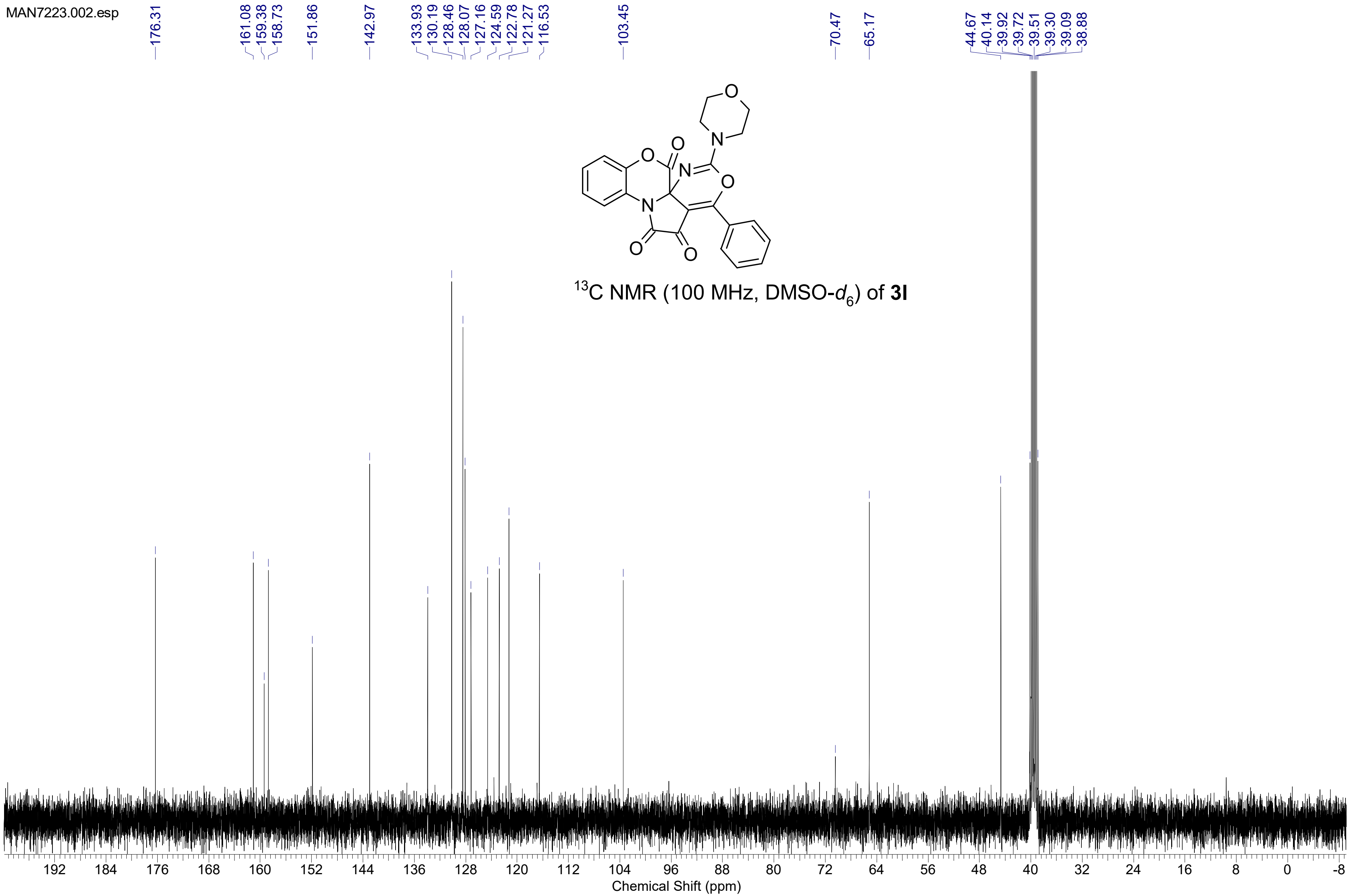

^{13}C NMR (100 MHz, DMSO-d_6) of **3l**

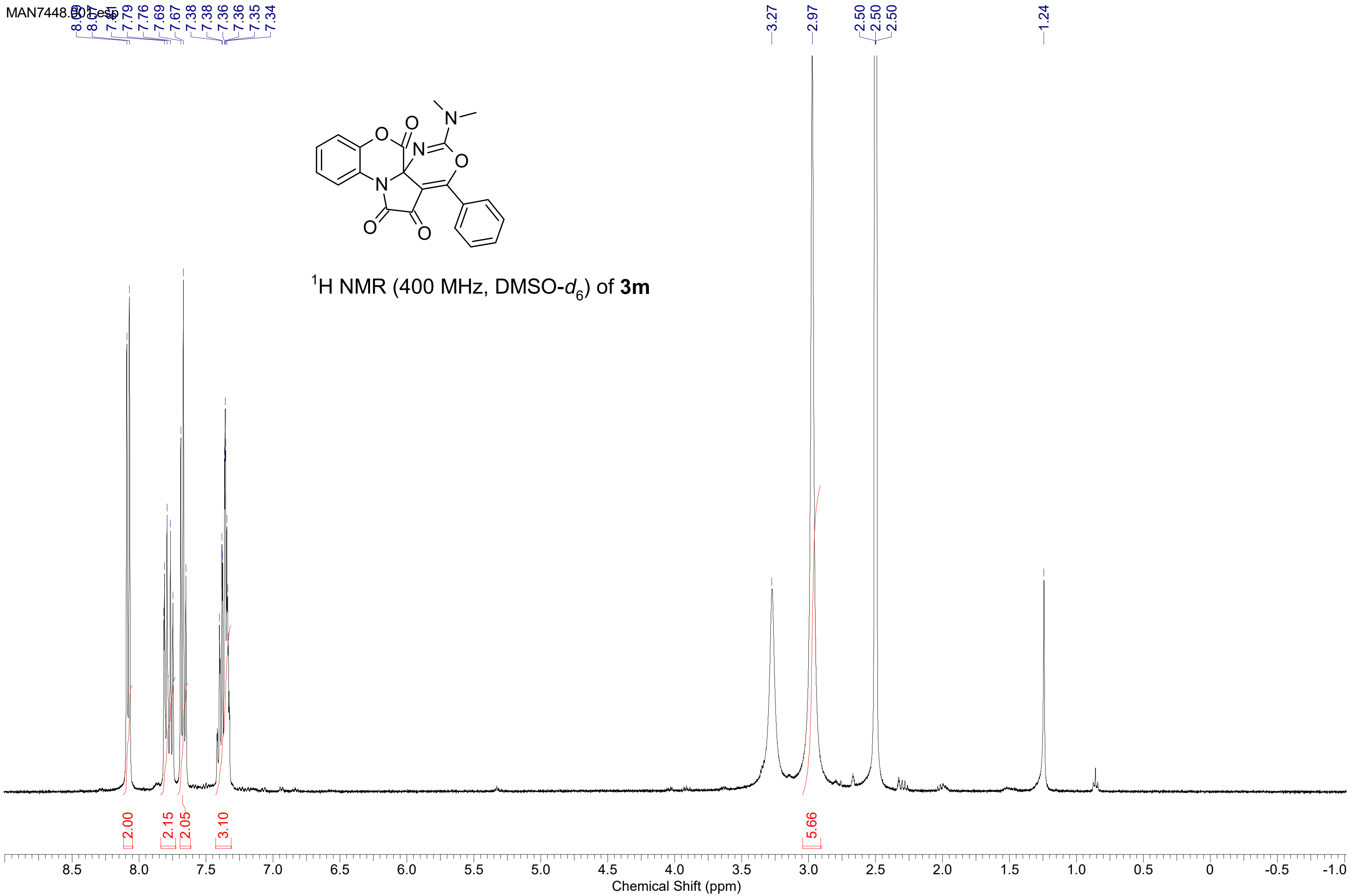

^{1}H NMR (400 MHz, DMSO-d_6) of **3m**

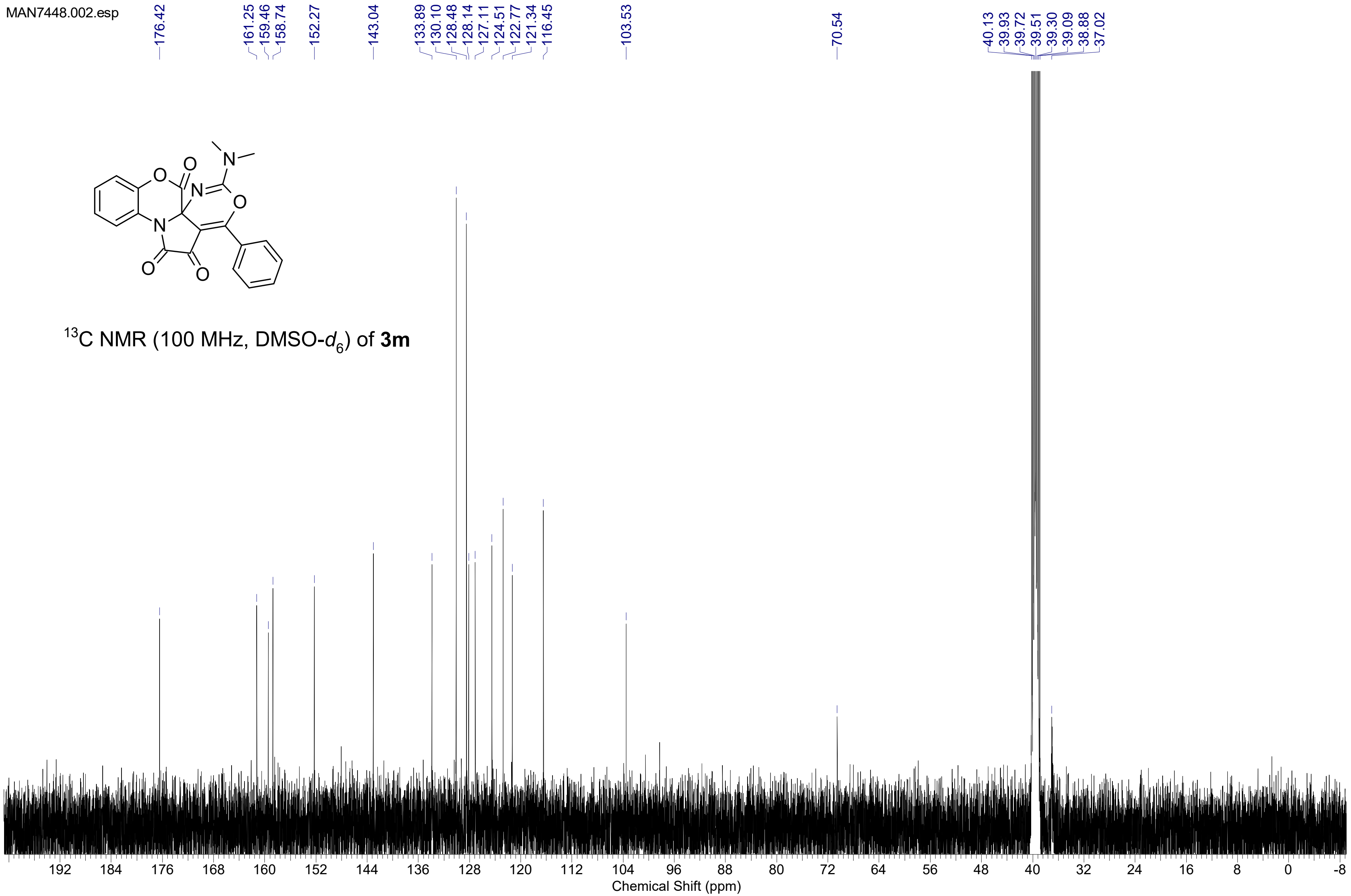

^{13}C NMR (100 MHz, DMSO-d_6) of **3m**

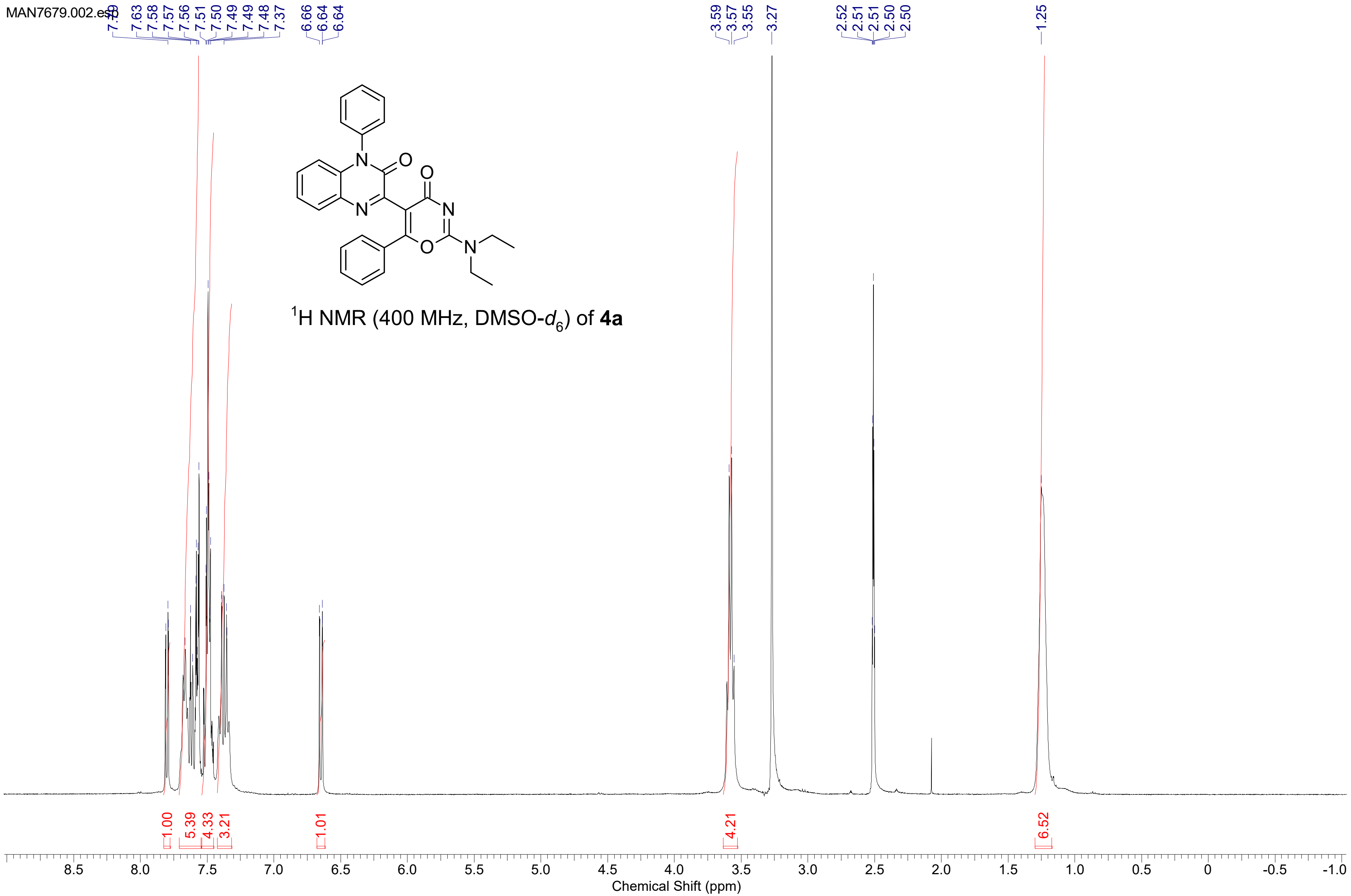

^{1}H NMR (400 MHz, DMSO-d_6) of **4a**

MAN7679.003.esp

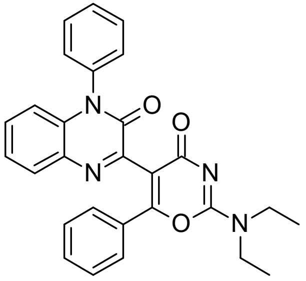

^{13}C NMR (100 MHz, DMSO-d_6) of **4a**

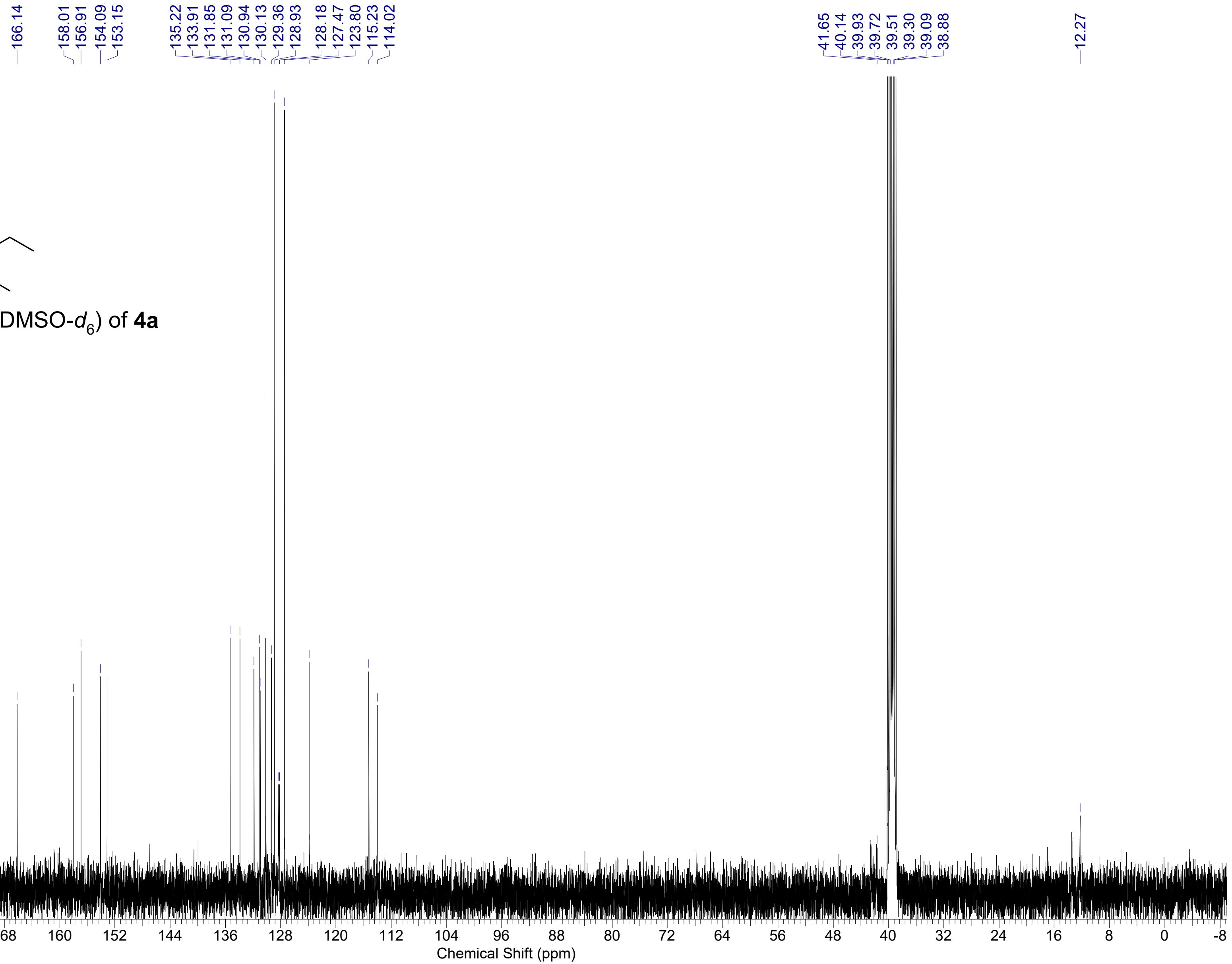

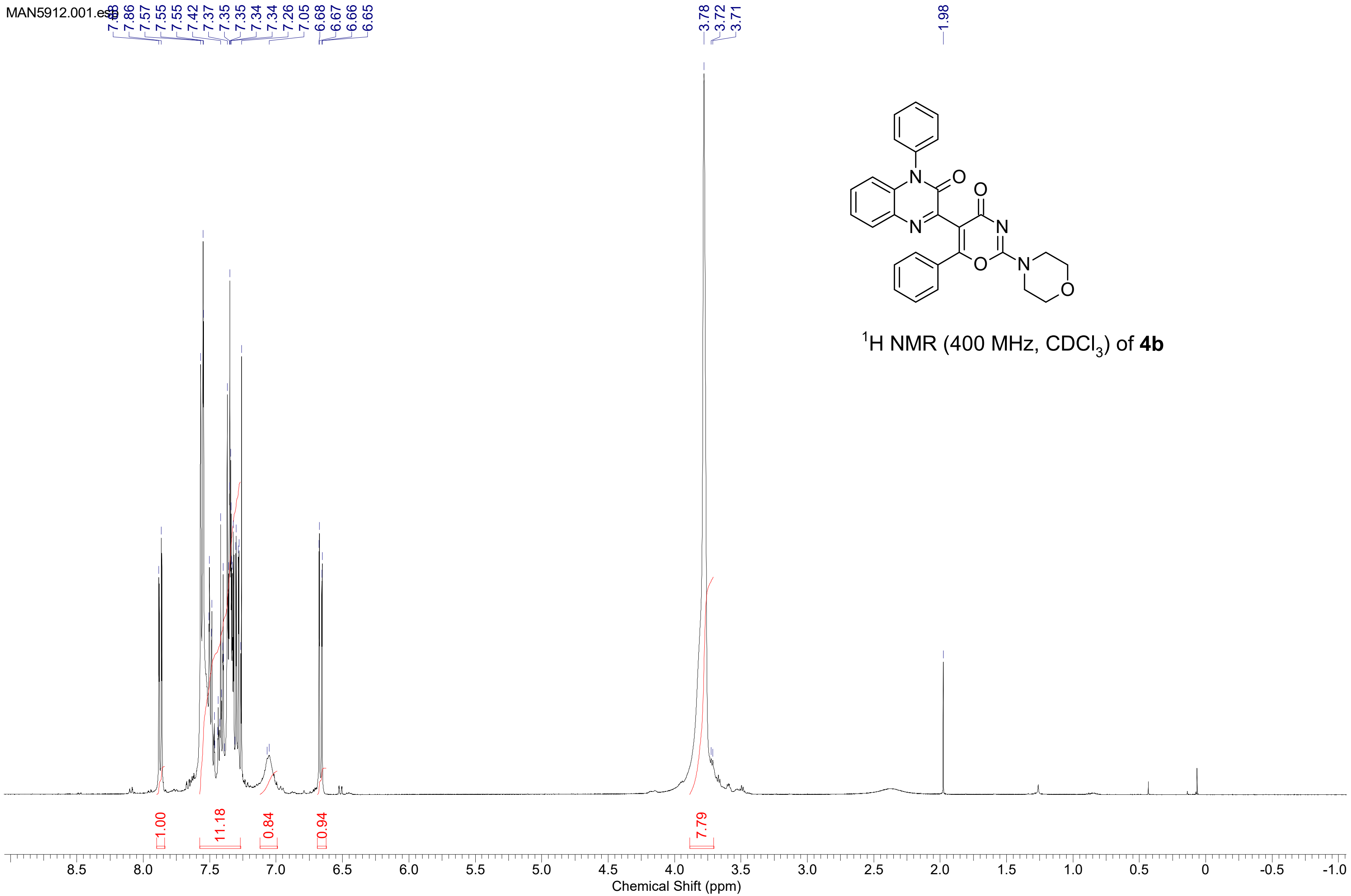

^{1}H NMR (400 MHz, $CDCl_3$) of **4b**

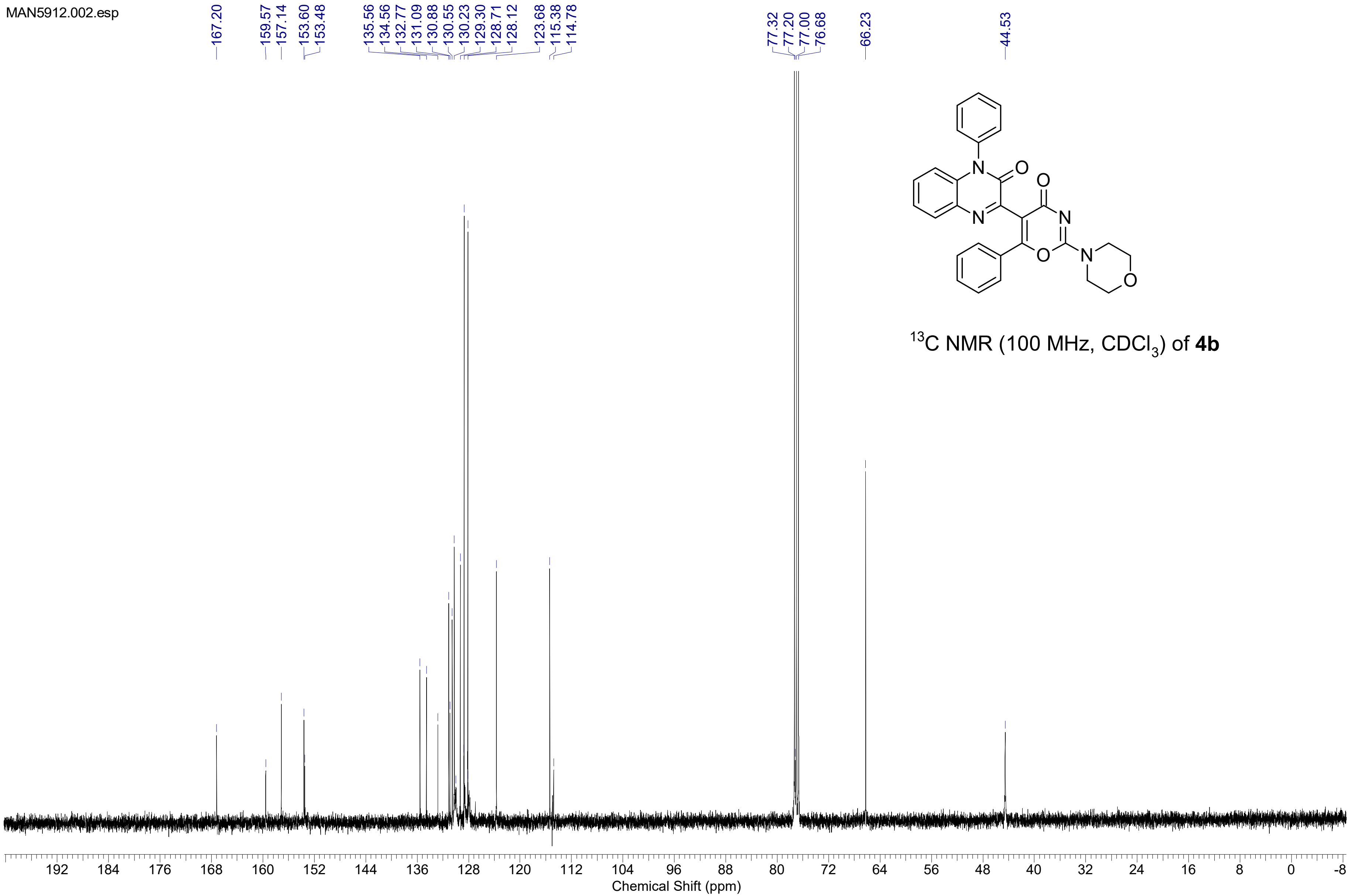

^{13}C NMR (100 MHz, $CDCl_3$) of **4b**

MAN7254.001.esp

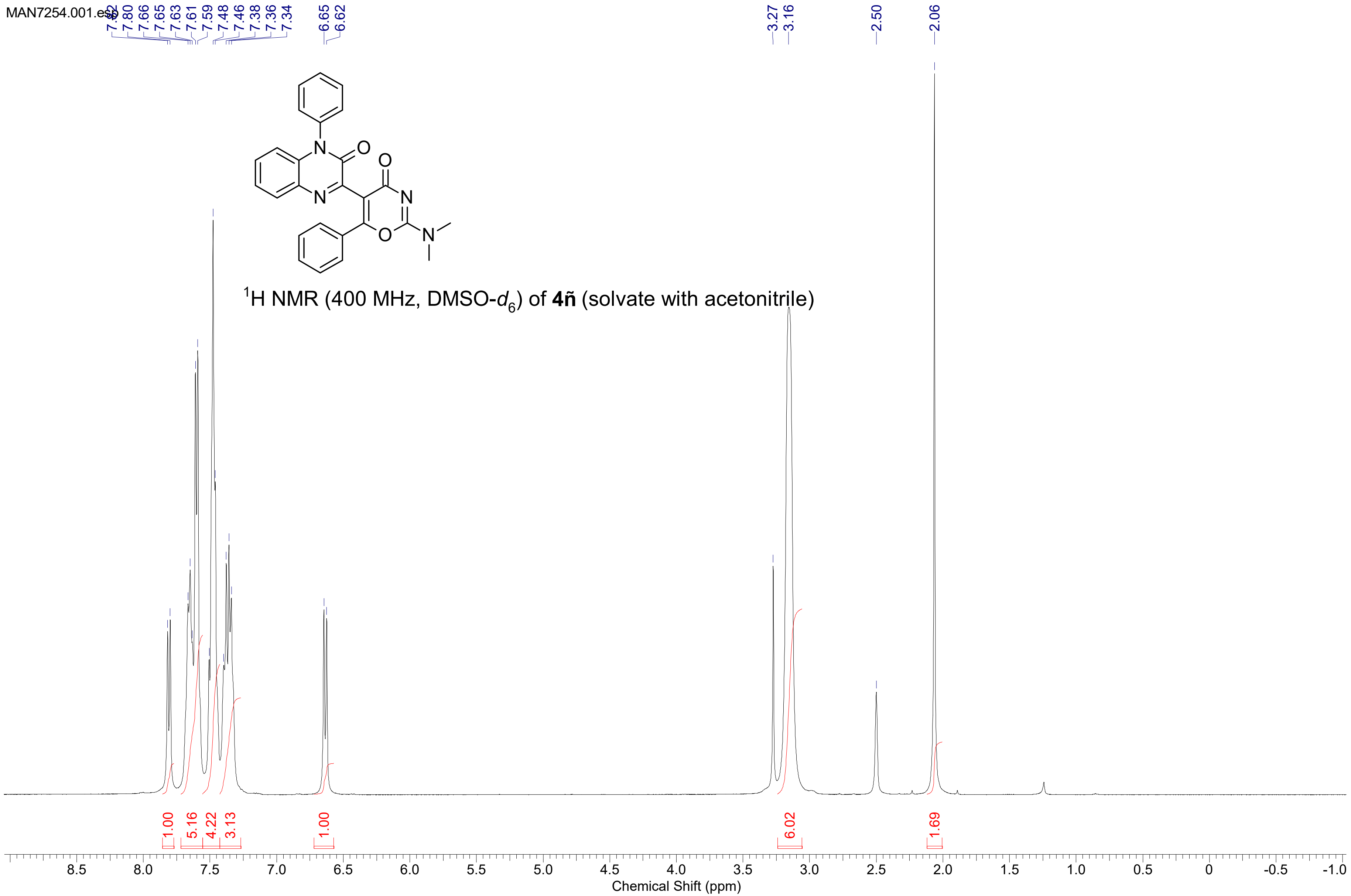

^{1}H NMR (400 MHz, DMSO-d_6) of **4ñ** (solvate with acetonitrile)

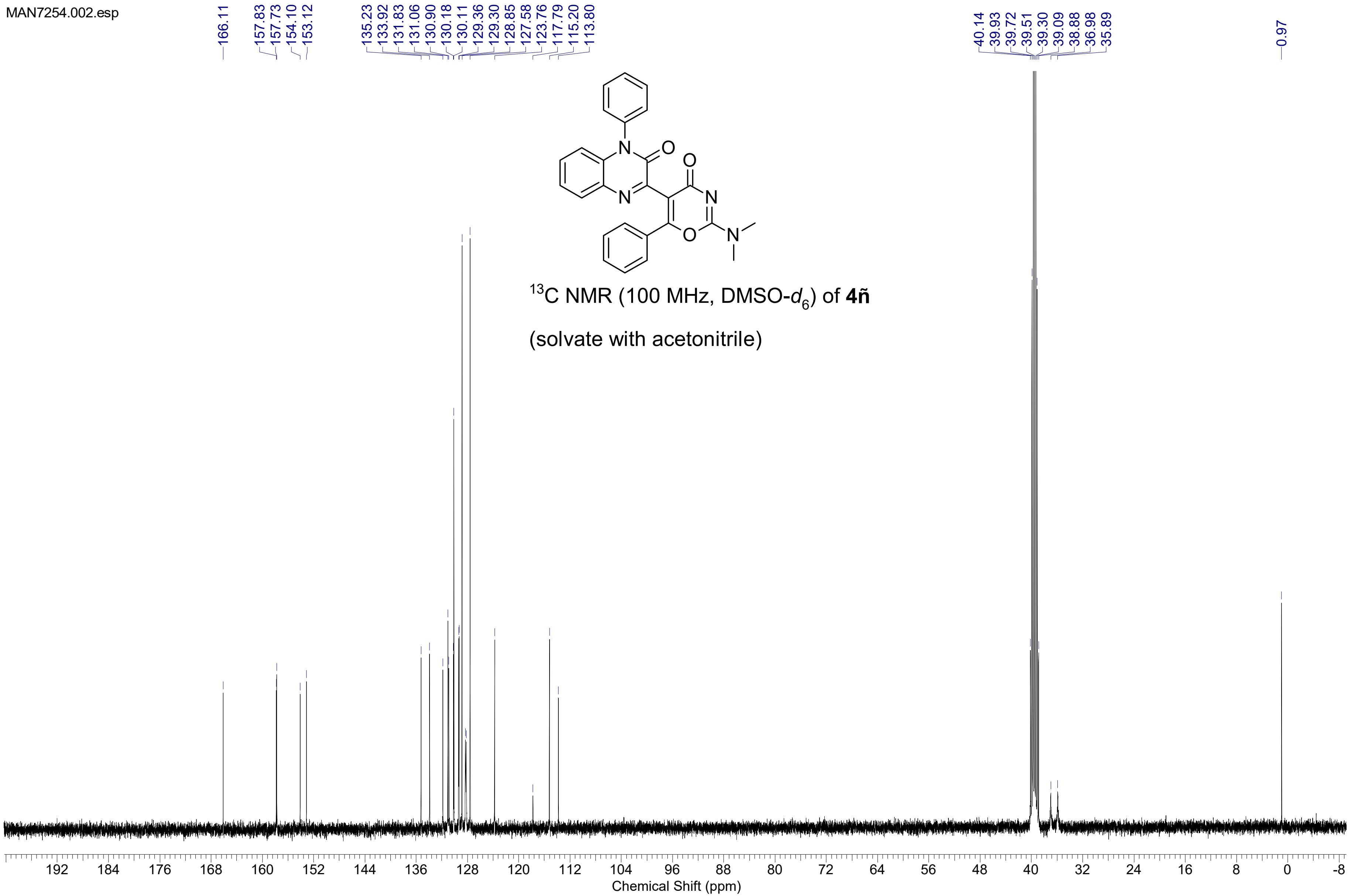
MAN7254.002.esp
166.11
157.83
157.73
154.10
153.12
135.23
133.92
131.83
131.06
130.90
130.18
130.11
129.36
129.30
128.85
127.58
123.76
117.79
115.20
113.80
40.14
39.93
39.72
39.51
39.30
39.09
38.88
36.98
35.89
0.97
O
N
O
N
N
O
N
^{13}C NMR (100 MHz, DMSO-d_6) of **4ñ**
(solvate with acetonitrile)
192 184 176 168 160 152 144 136 128 120 112 104 96 88 80 72 64 56 48 40 32 24 16 8 0 -8
Chemical Shift (ppm)

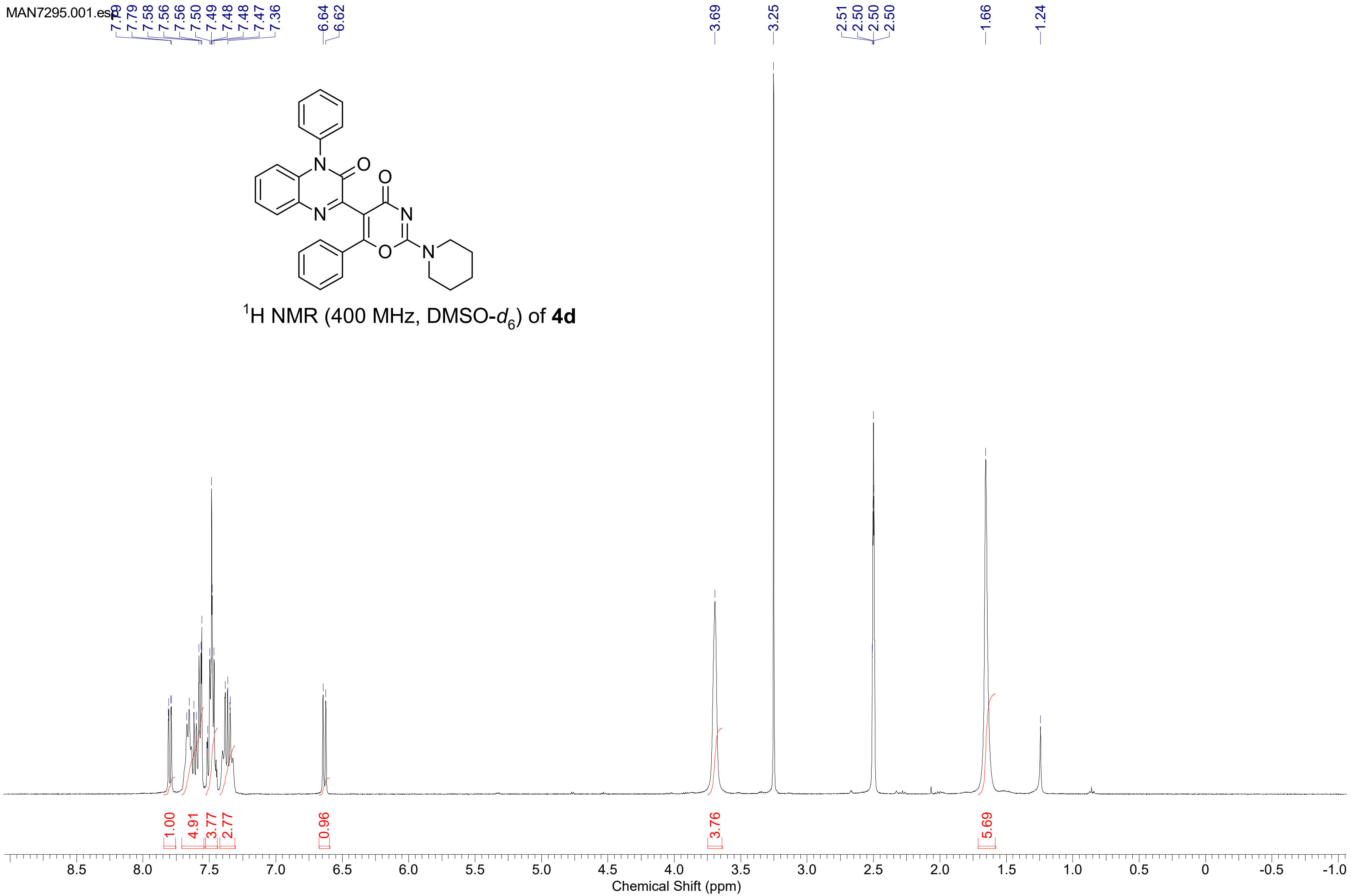

^{1}H NMR (400 MHz, DMSO-d_6) of **4d**

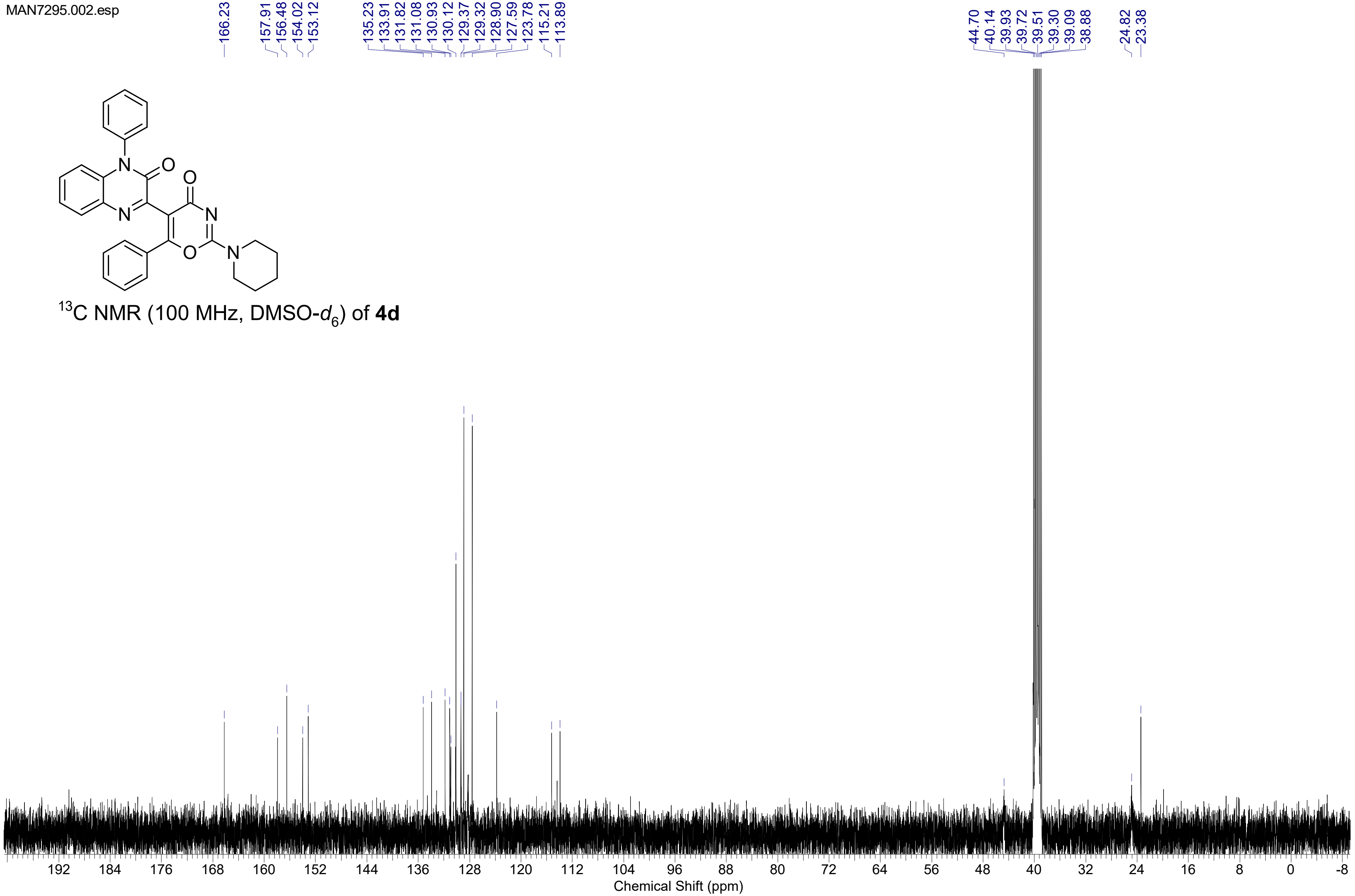

^{13}C NMR (100 MHz, DMSO-d_6) of **4d**

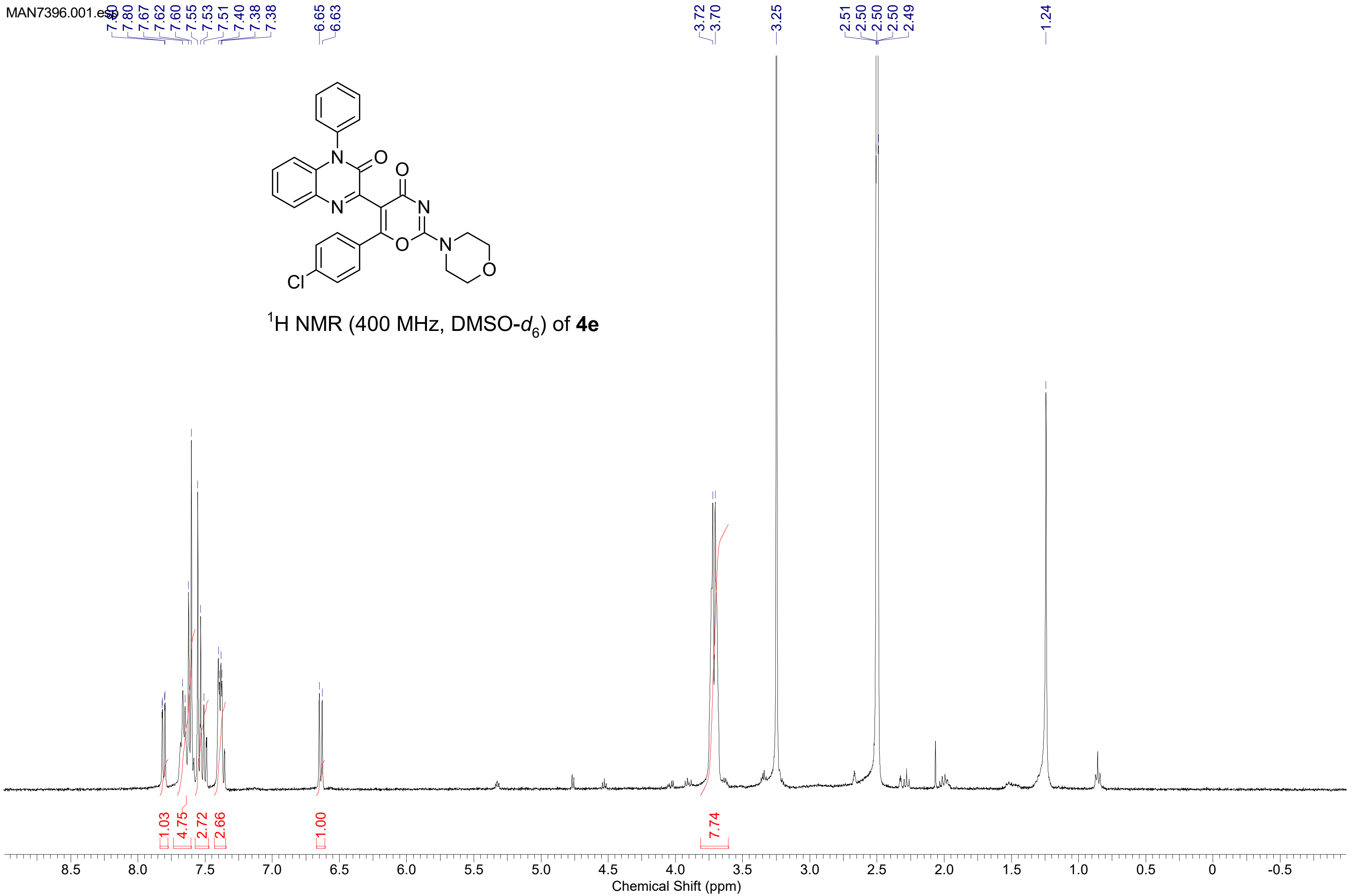

^{1}H NMR (400 MHz, DMSO-d_6) of **4e**

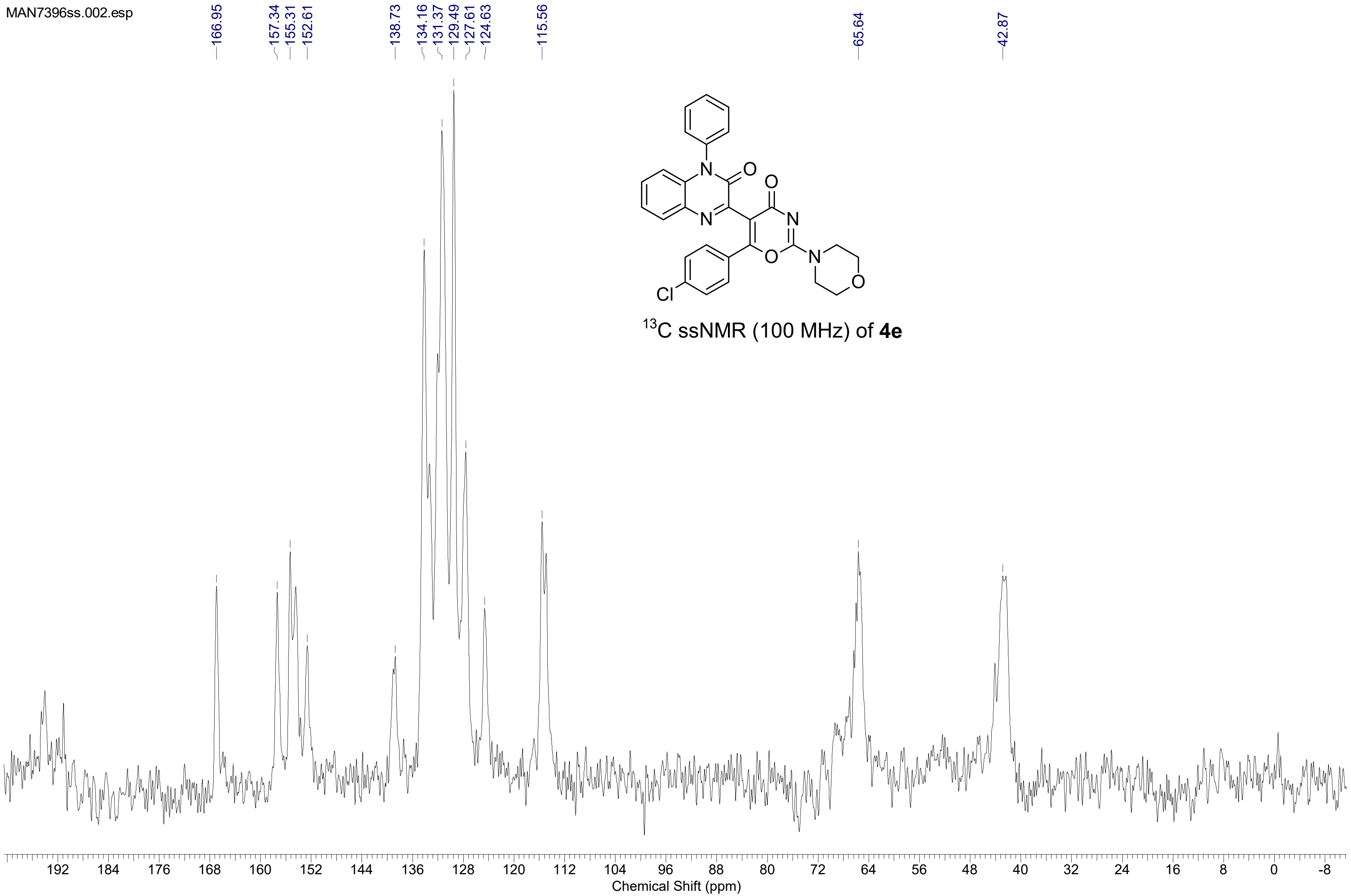

^{13}C ssNMR (100 MHz) of **4e**

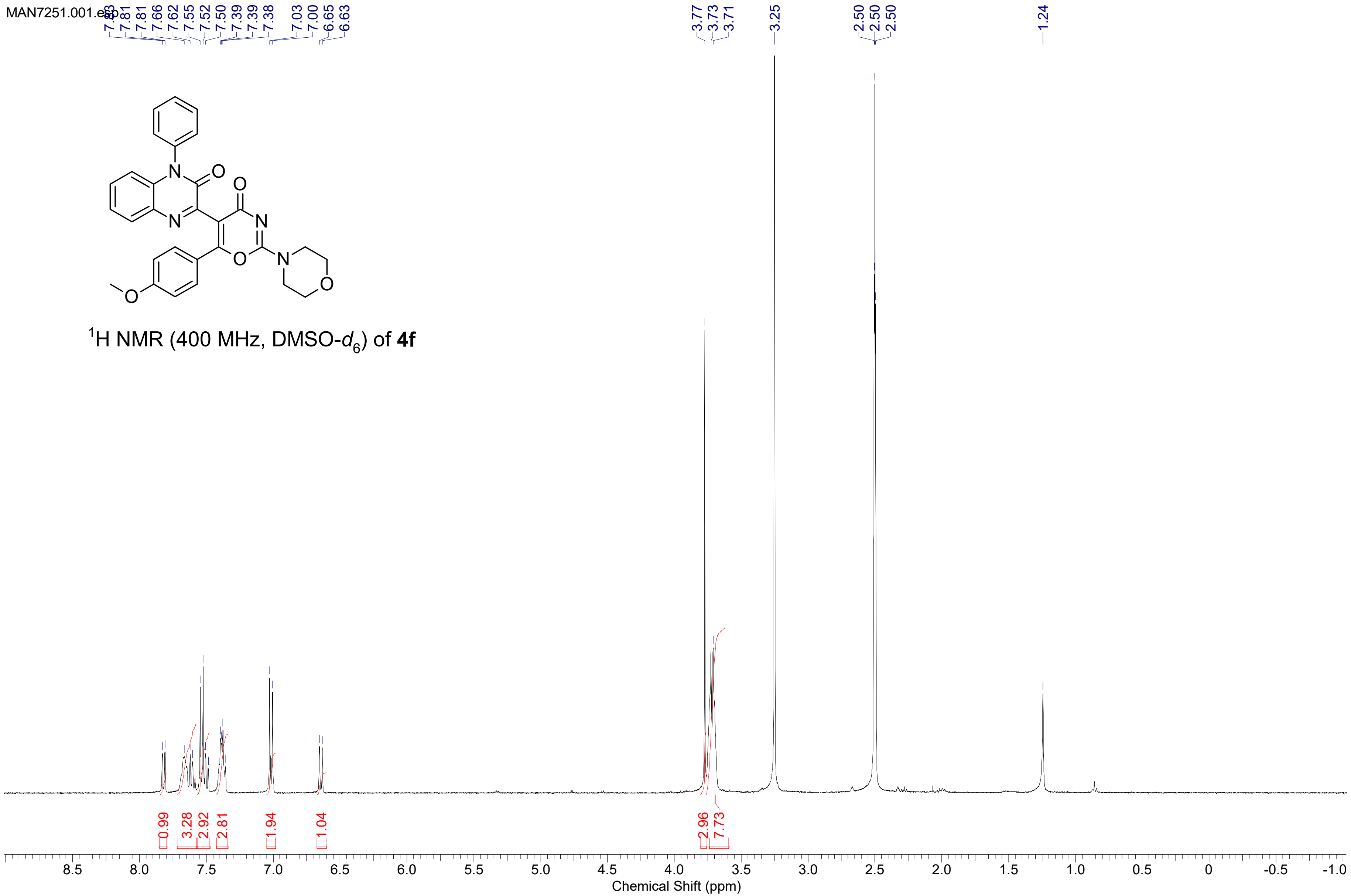

^{1}H NMR (400 MHz, DMSO-d_6) of **4f**

MAN7251.004.esp

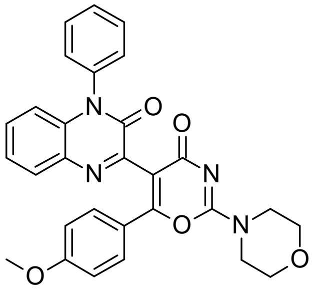

^{13}C NMR (100 MHz, DMSO-d_6) of **4f**

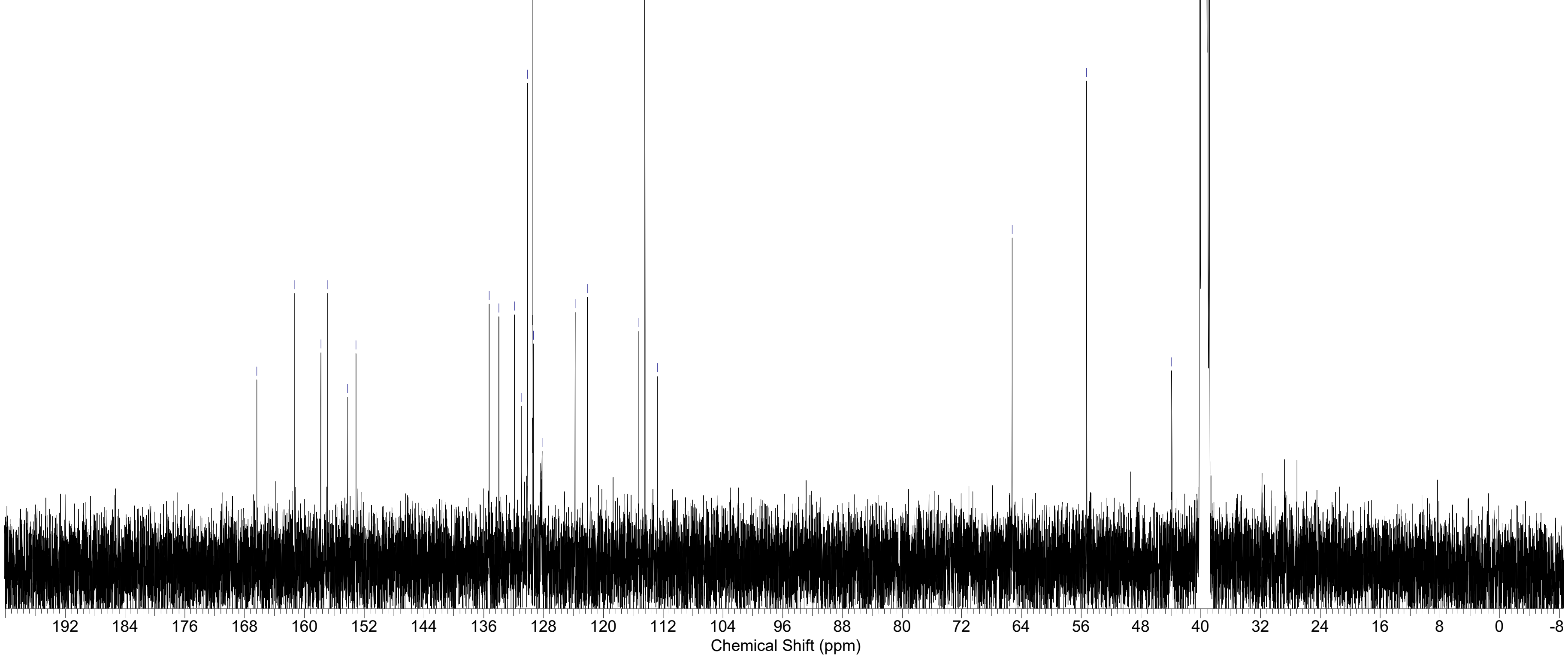

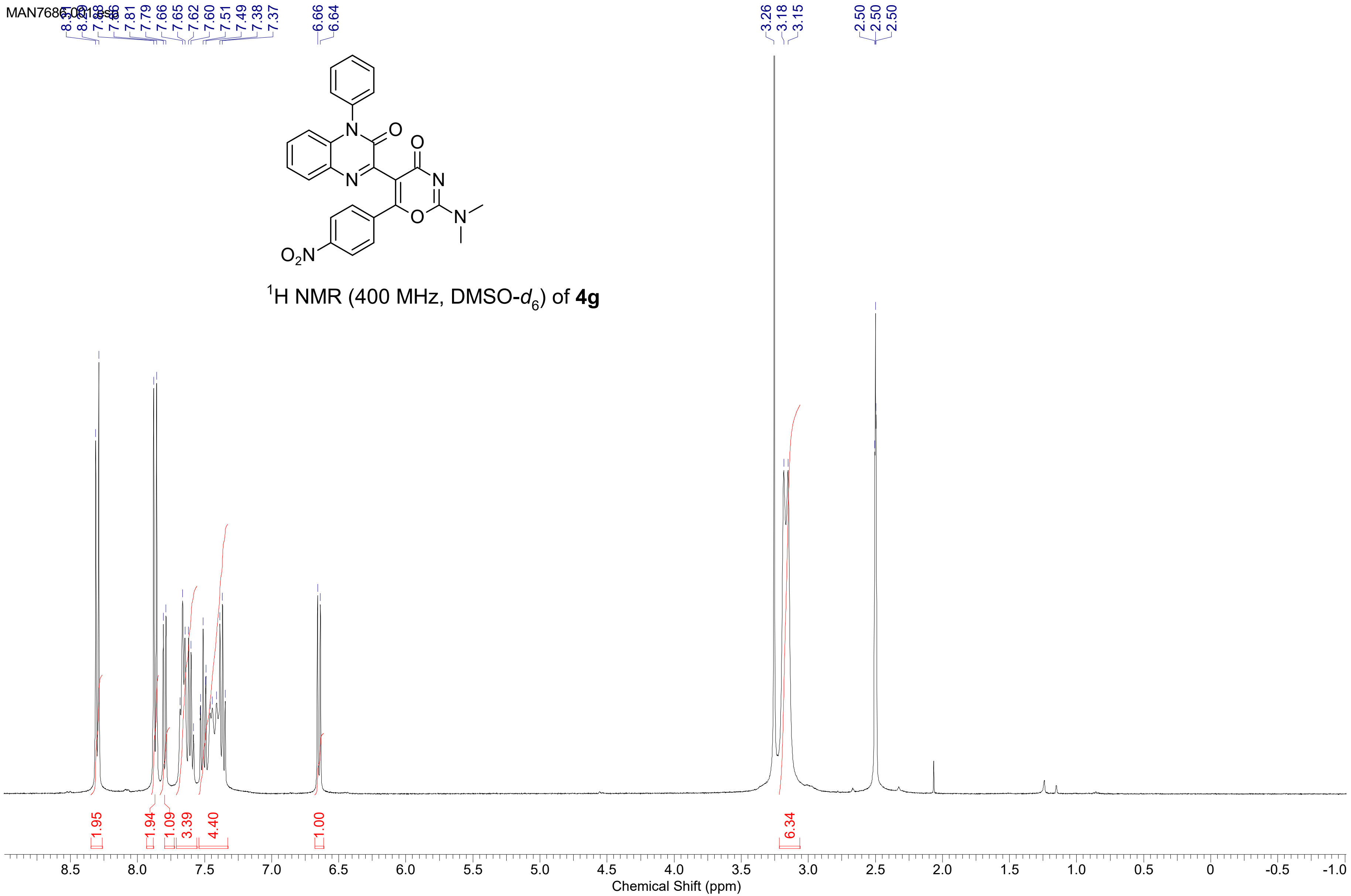

^{1}H NMR (400 MHz, DMSO-d_6) of **4g**

MAN7686.002.esp

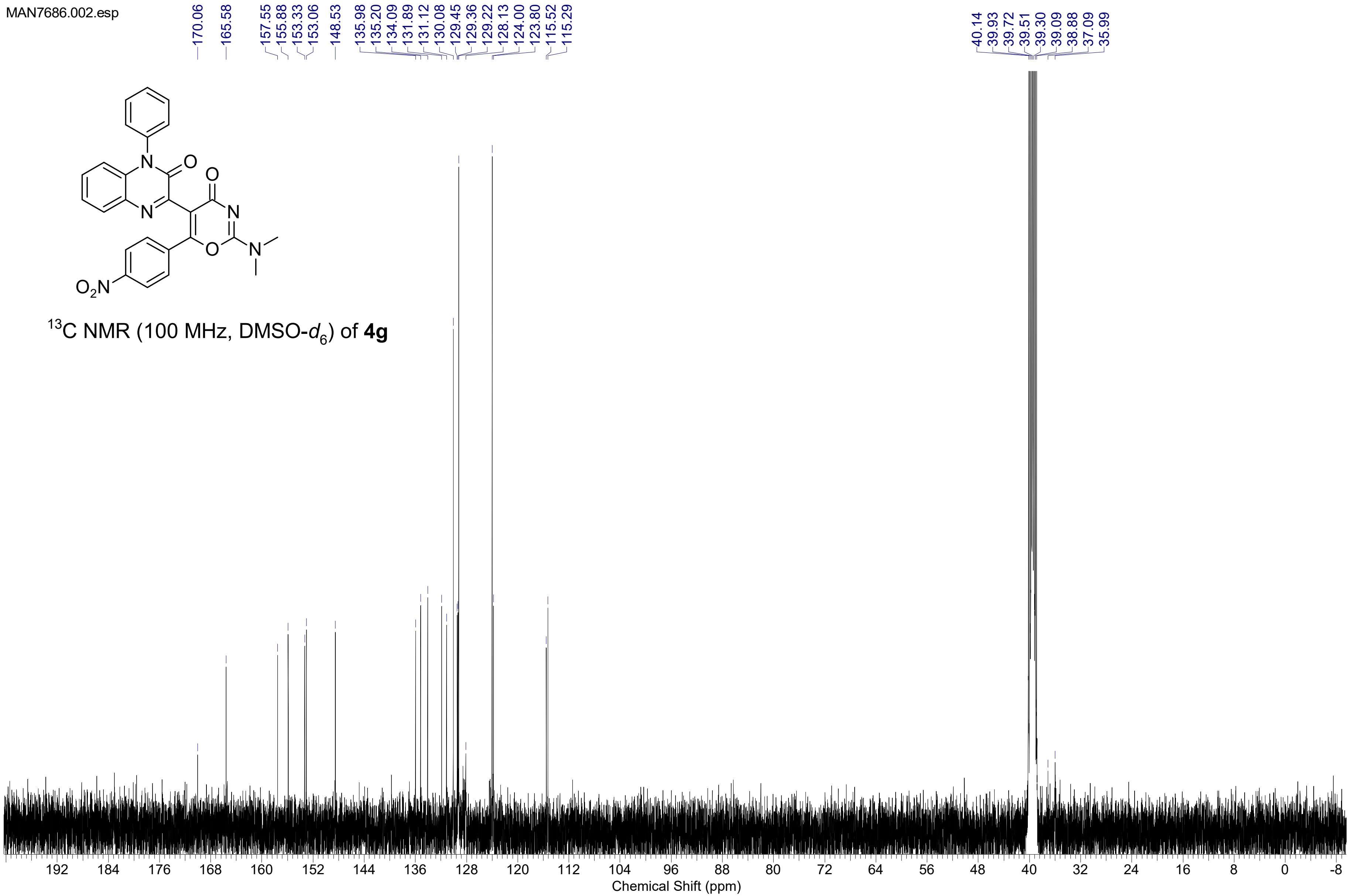

^{13}C NMR (100 MHz, DMSO-d_6) of **4g**

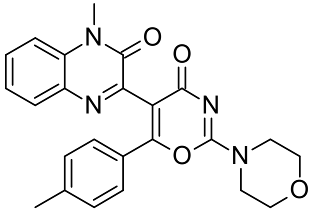

^1H NMR (400 MHz, DMSO-d_6) of **4h**

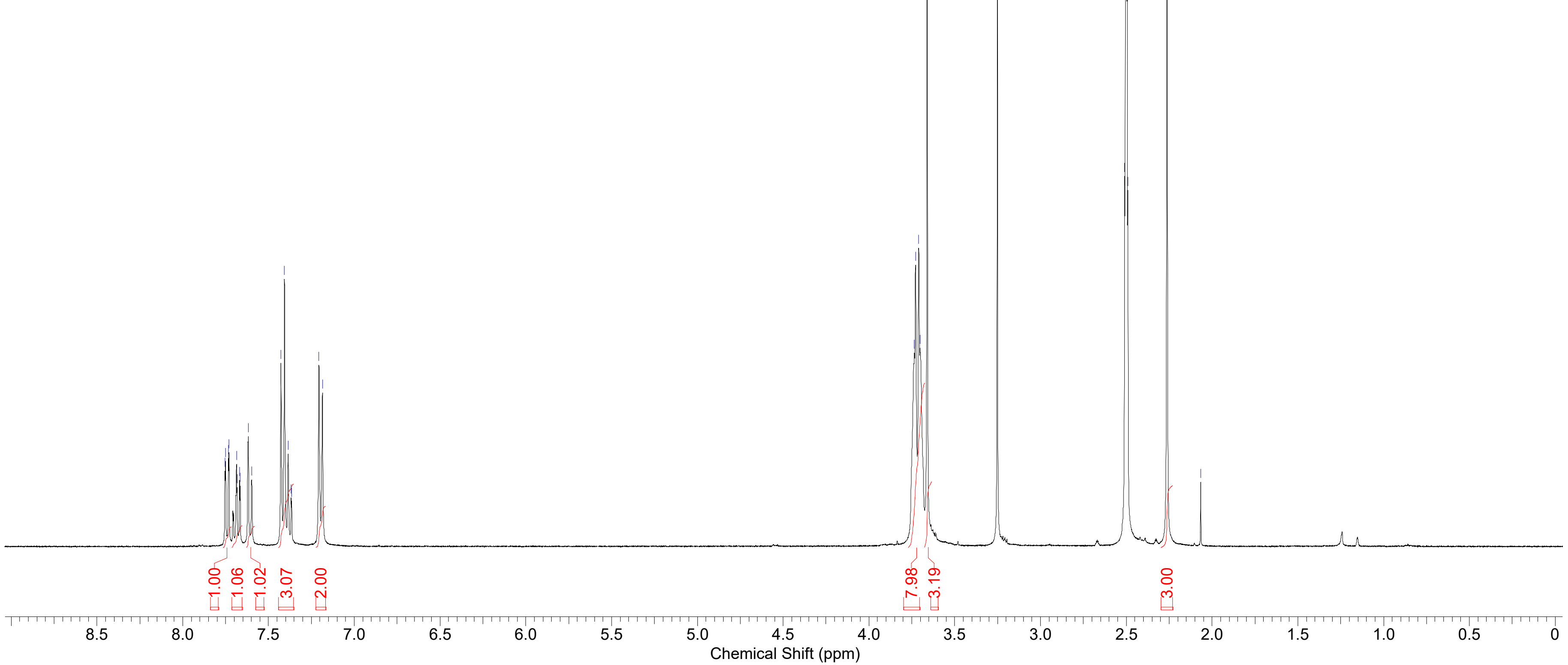

MAN7575ss.002.esp

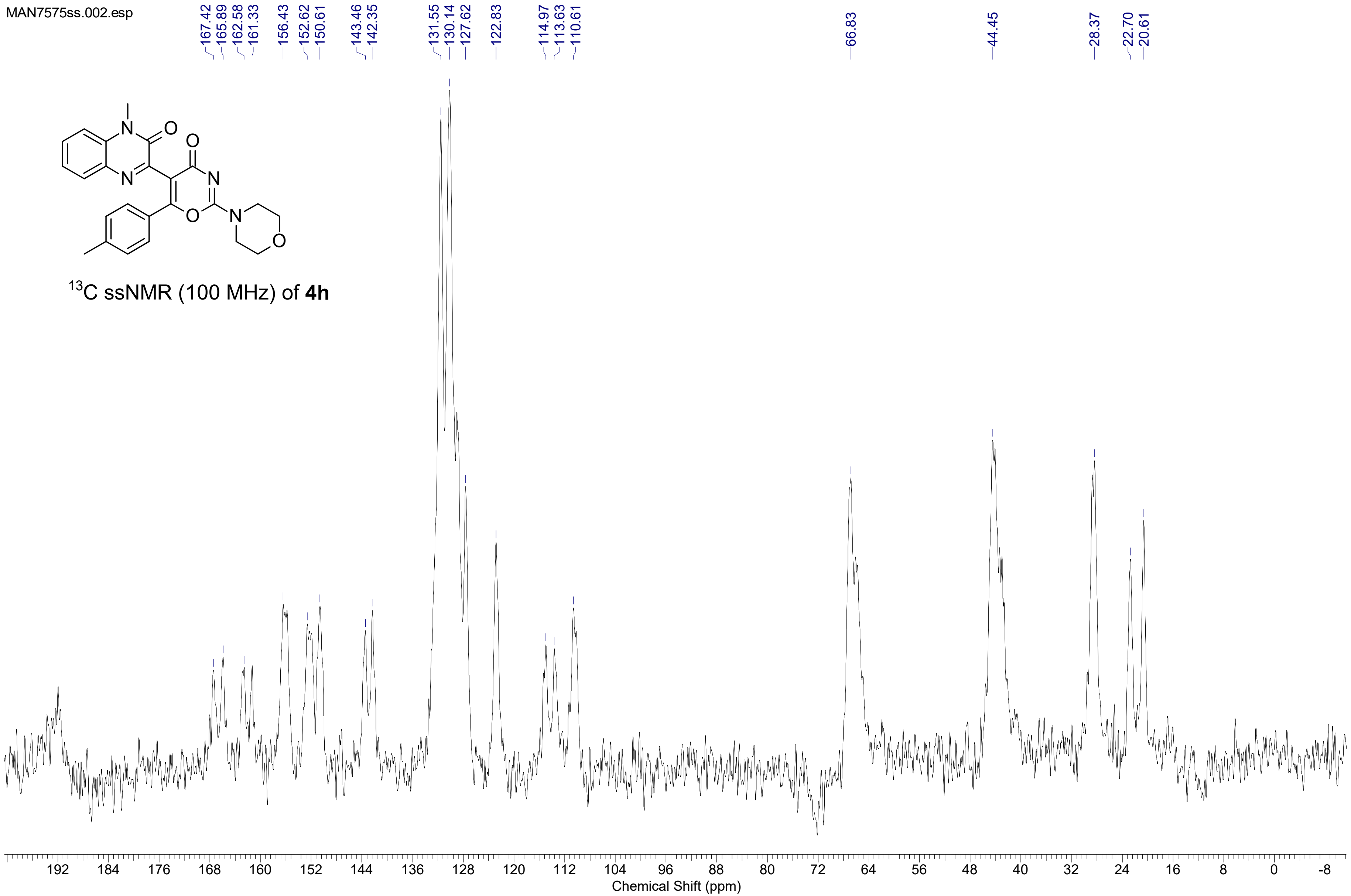

^{13}C ssNMR (100 MHz) of **4h**

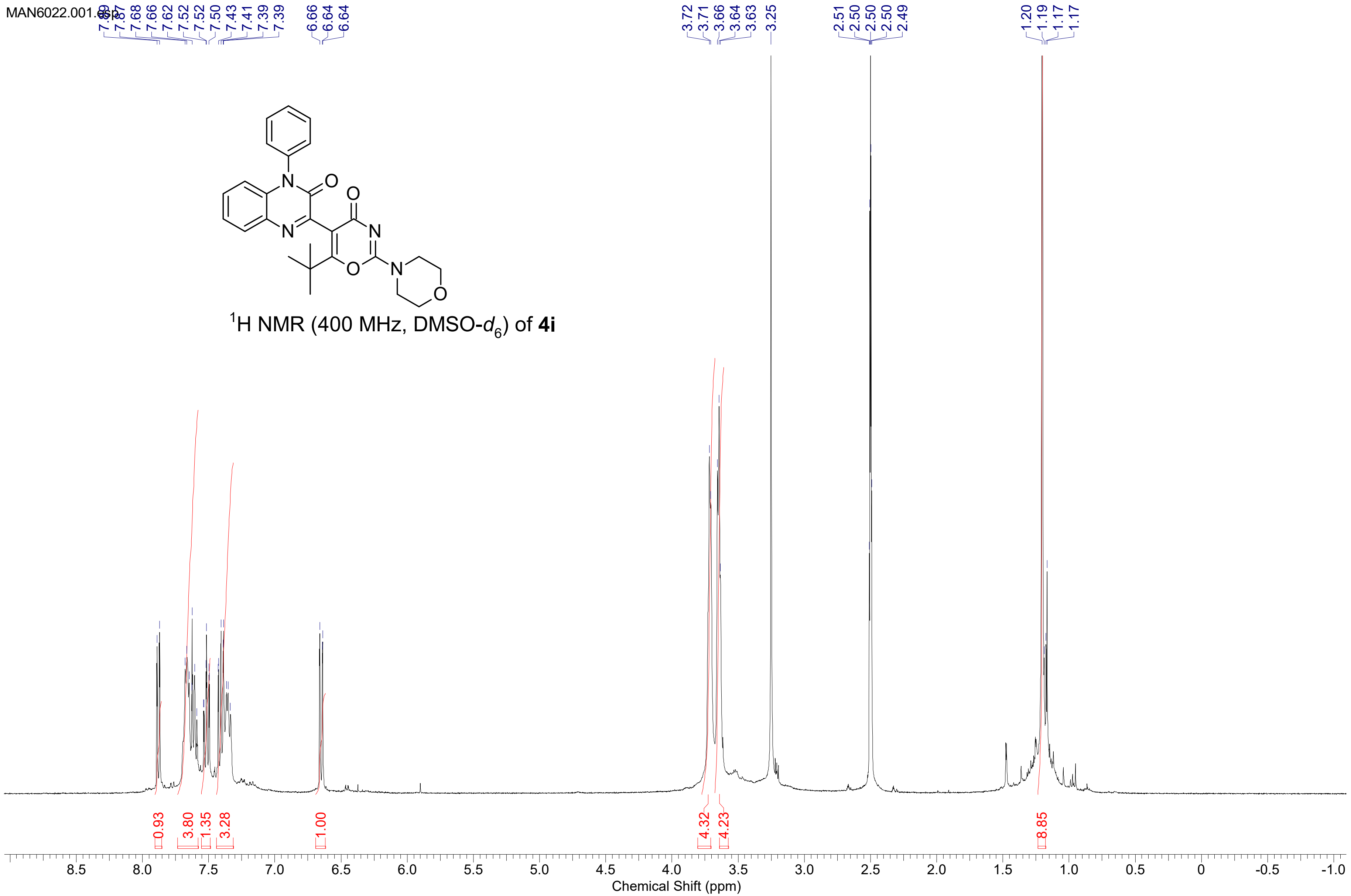

^{1}H NMR (400 MHz, DMSO-d_6) of **4i**

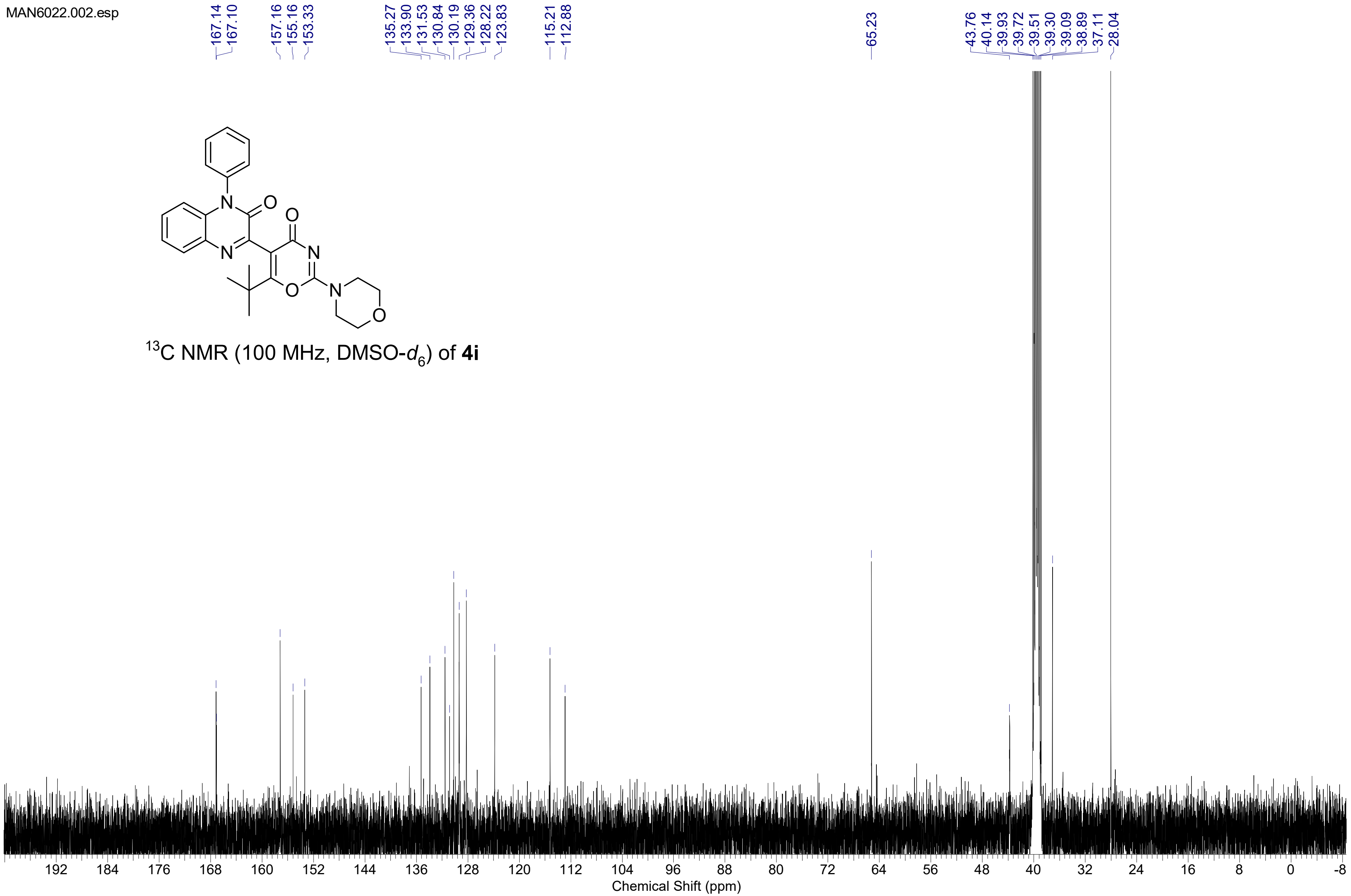
MAN6022.002.esp
167.14
167.10
157.16
155.16
153.33
135.27
133.90
131.53
130.84
130.19
129.36
128.22
123.83
115.21
112.88
65.23
43.76
40.14
39.93
39.72
39.51
39.30
39.09
38.89
37.11
28.04
^{13}C NMR (100 MHz, DMSO-d_6) of **4i**
Chemical Shift (ppm)

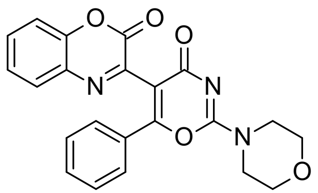

^1H NMR (400 MHz, DMSO-d_6) of **4l**

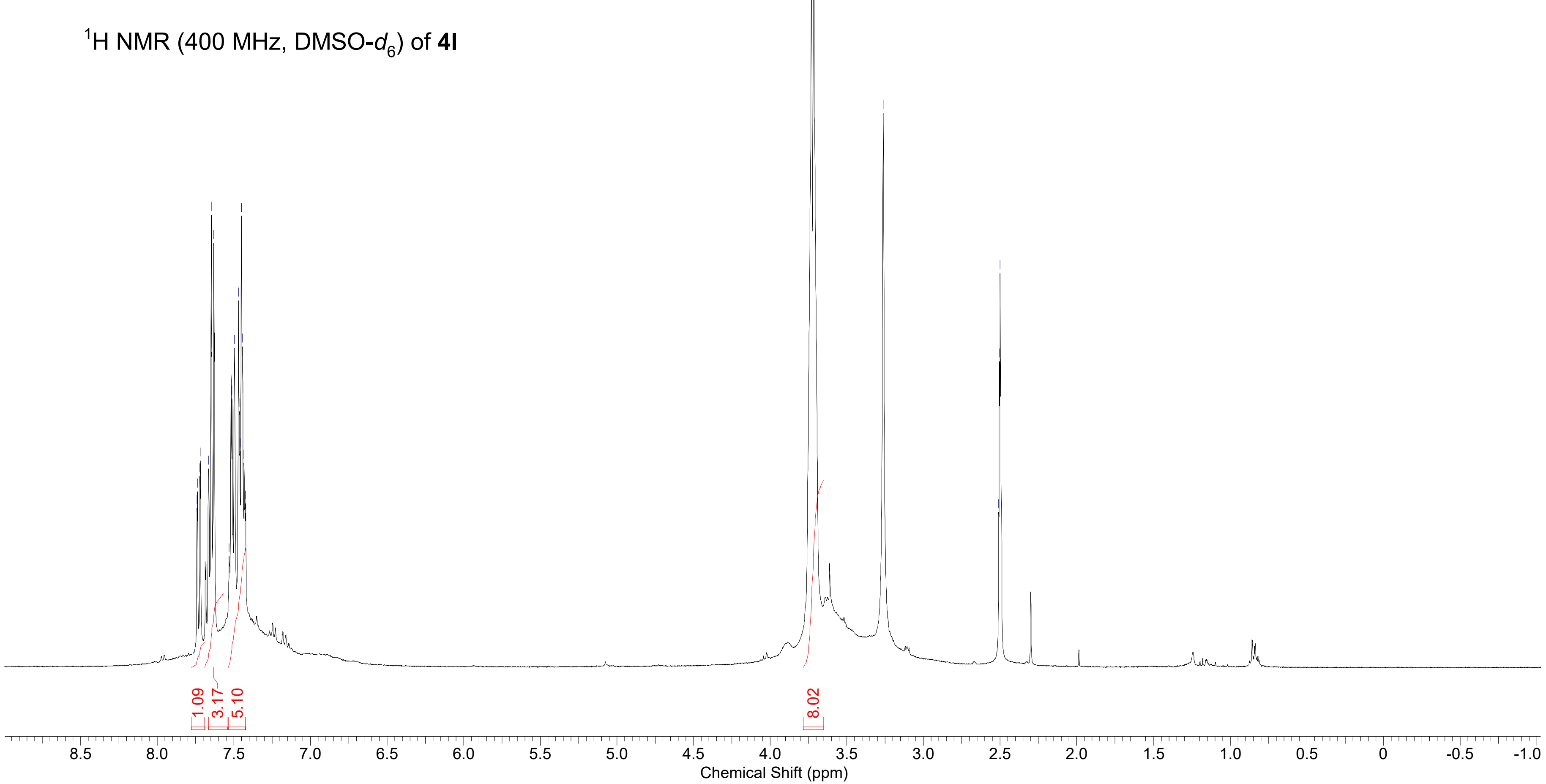

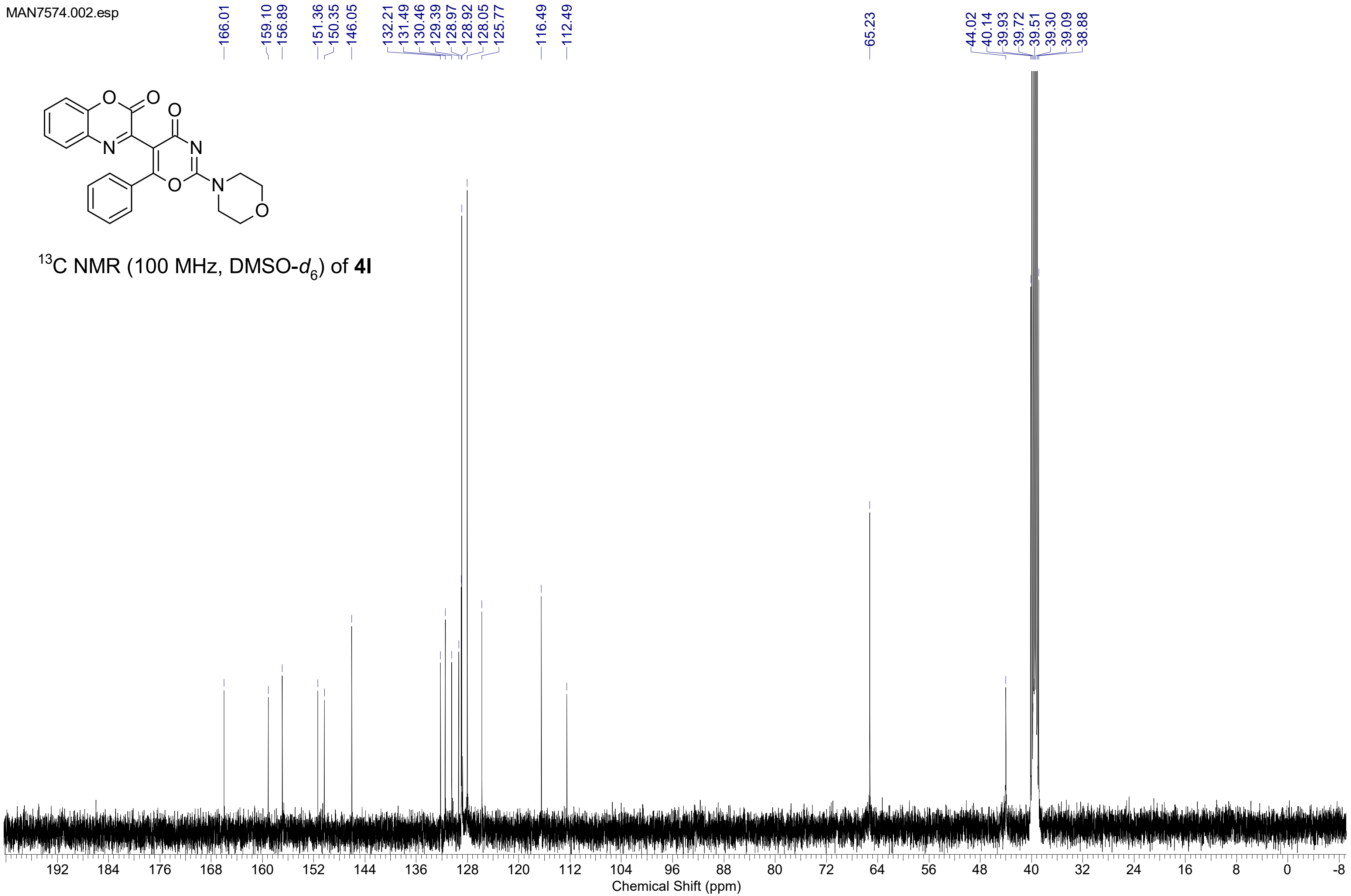

^{13}C NMR (100 MHz, DMSO-d_6) of **4l**

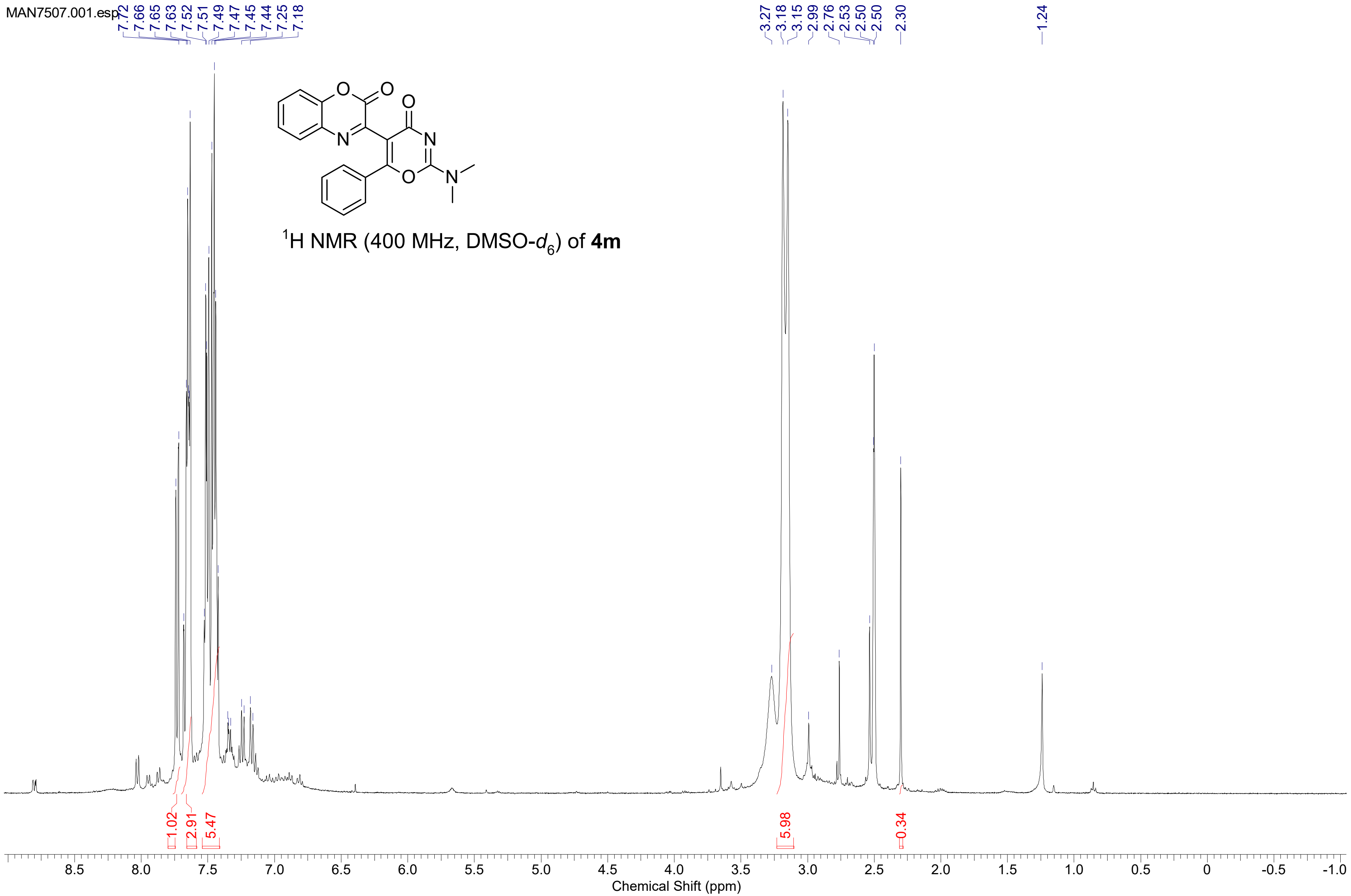

^{1}H NMR (400 MHz, DMSO-d_6) of **4m**

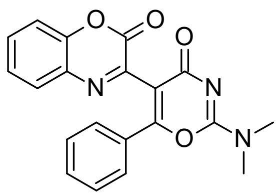

^{13}C NMR (100 MHz, DMSO-d_6) of **4m**

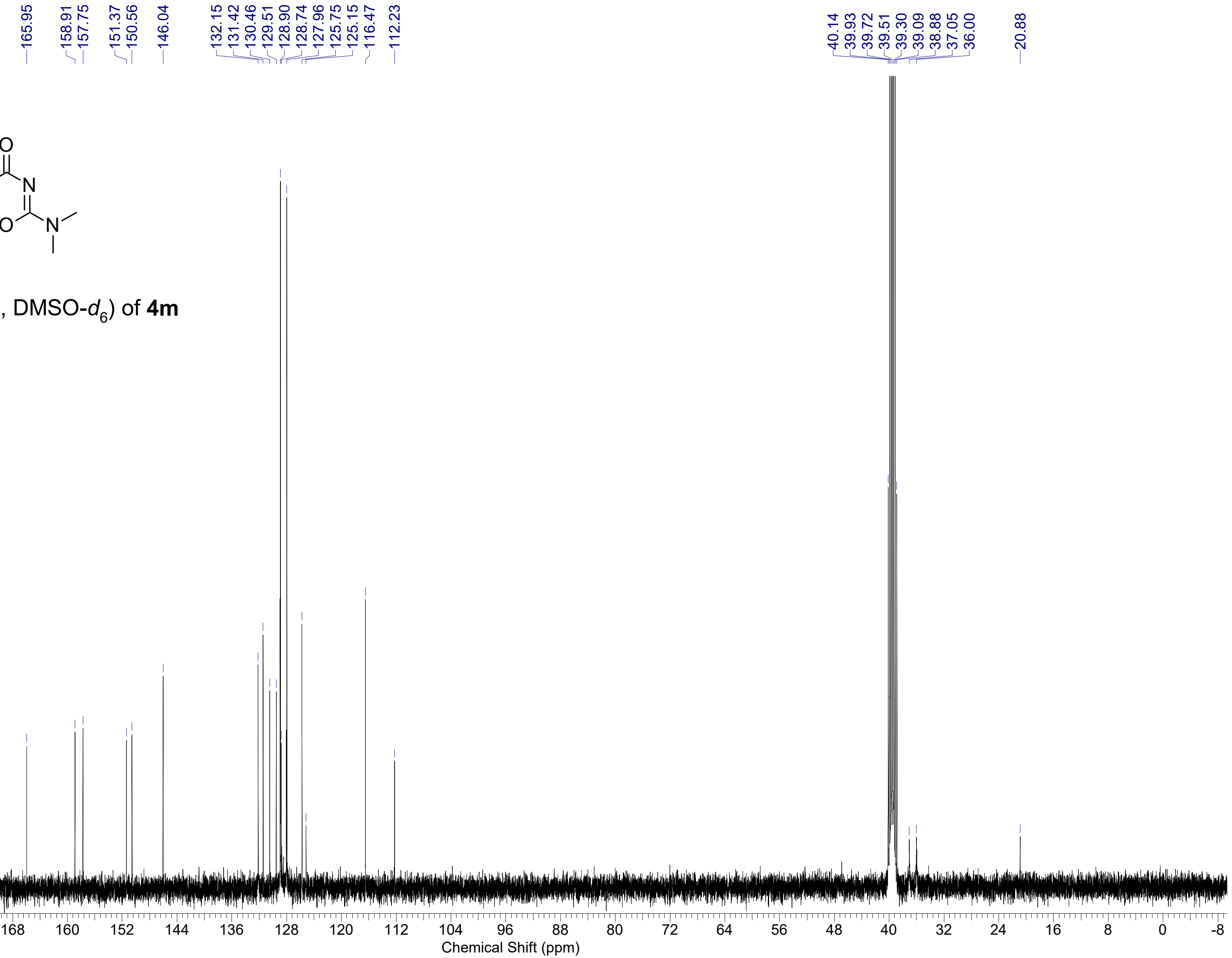

NMR charts of compound I

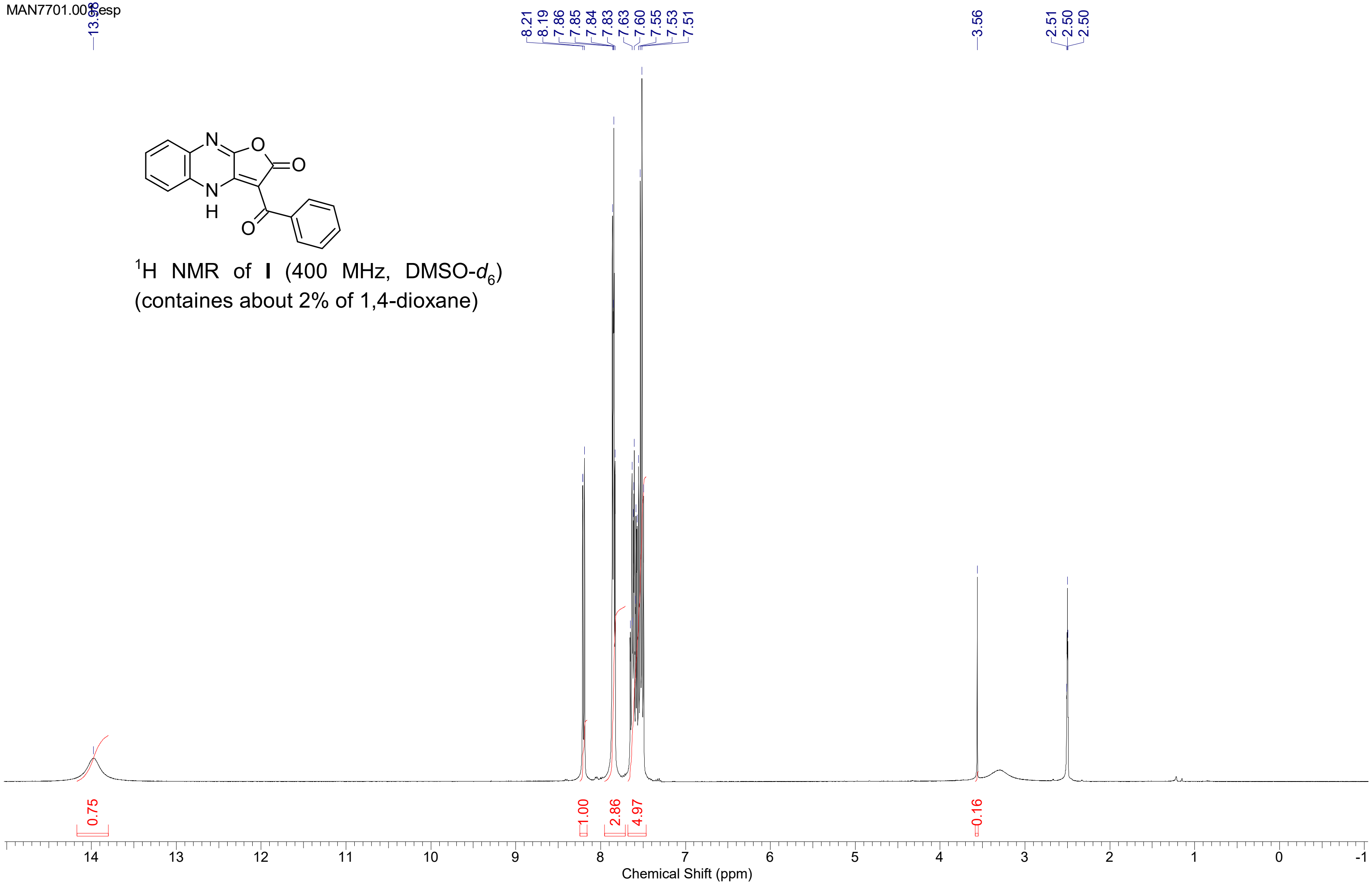

^{1}H NMR of **I** (400 MHz, DMSO-d_6)
(containes about 2% of 1,4-dioxane)

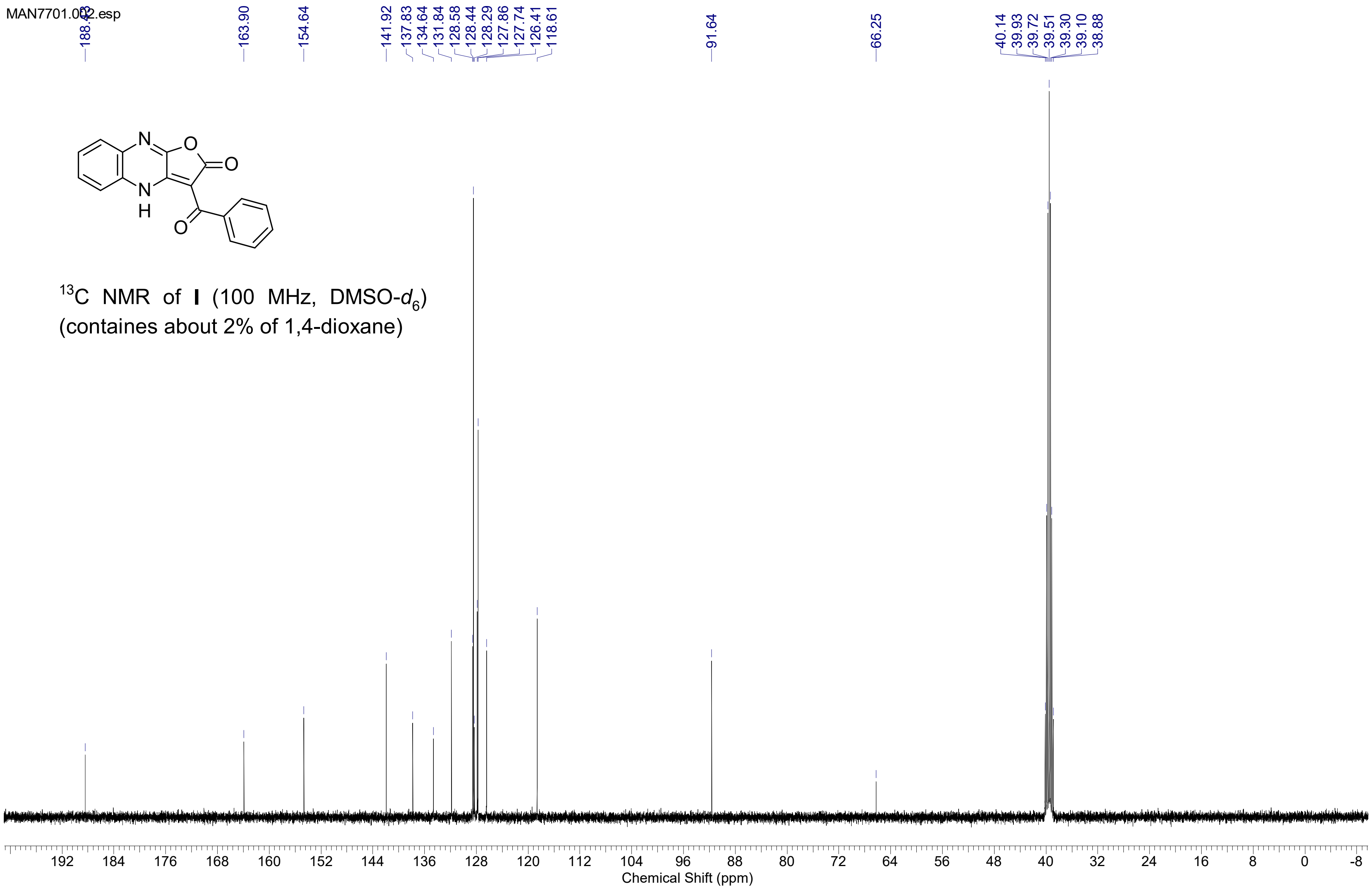
MAN7701.012.esp
188.48
163.90
154.64
141.92
137.83
134.64
131.84
128.58
128.44
128.29
127.86
127.74
126.41
118.61
91.64
66.25
40.14
39.93
39.72
39.51
39.30
39.10
38.88
N
O
O
N
H
O
^{13}C NMR of **I** (100 MHz, DMSO-d_6)
(containes about 2% of 1,4-dioxane)
192 184 176 168 160 152 144 136 128 120 112 104 96 88 80 72 64 56 48 40 32 24 16 8 0 -8
Chemical Shift (ppm)

ORTEP images of X-ray crystal structures

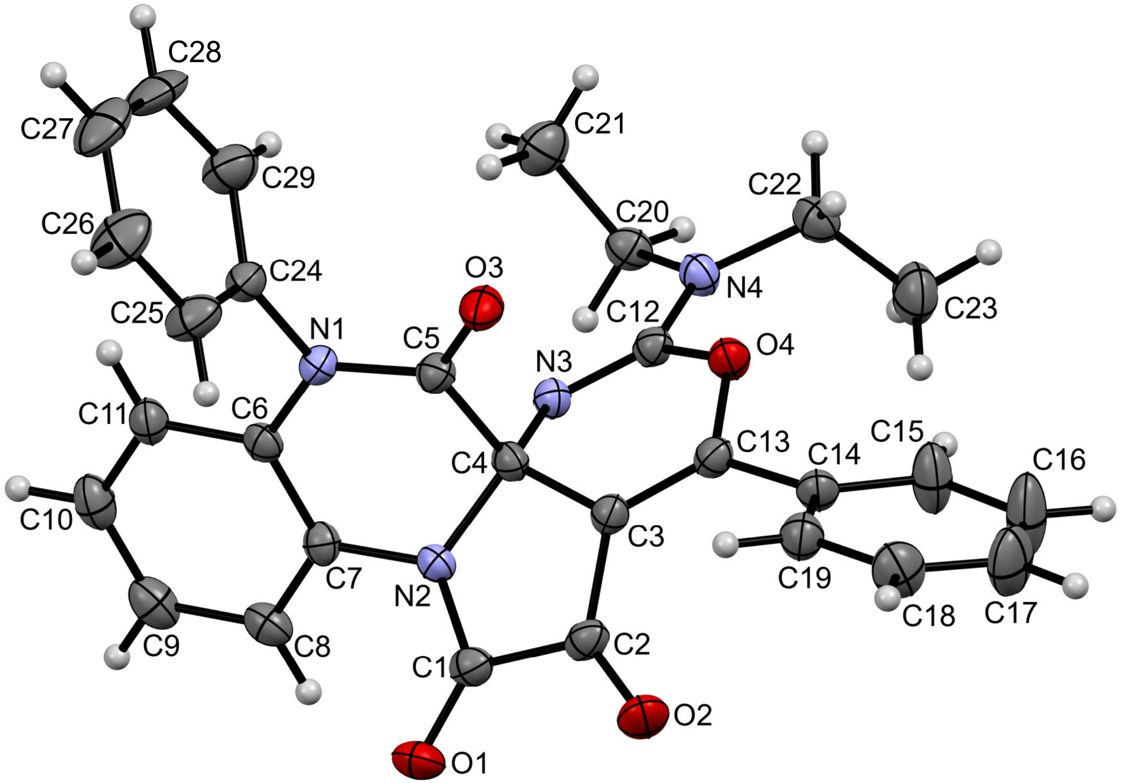

Figure S1. Molecular structure of compound **3a** showing 30% probability amplitude displacement ellipsoids (CCDC 2192396).

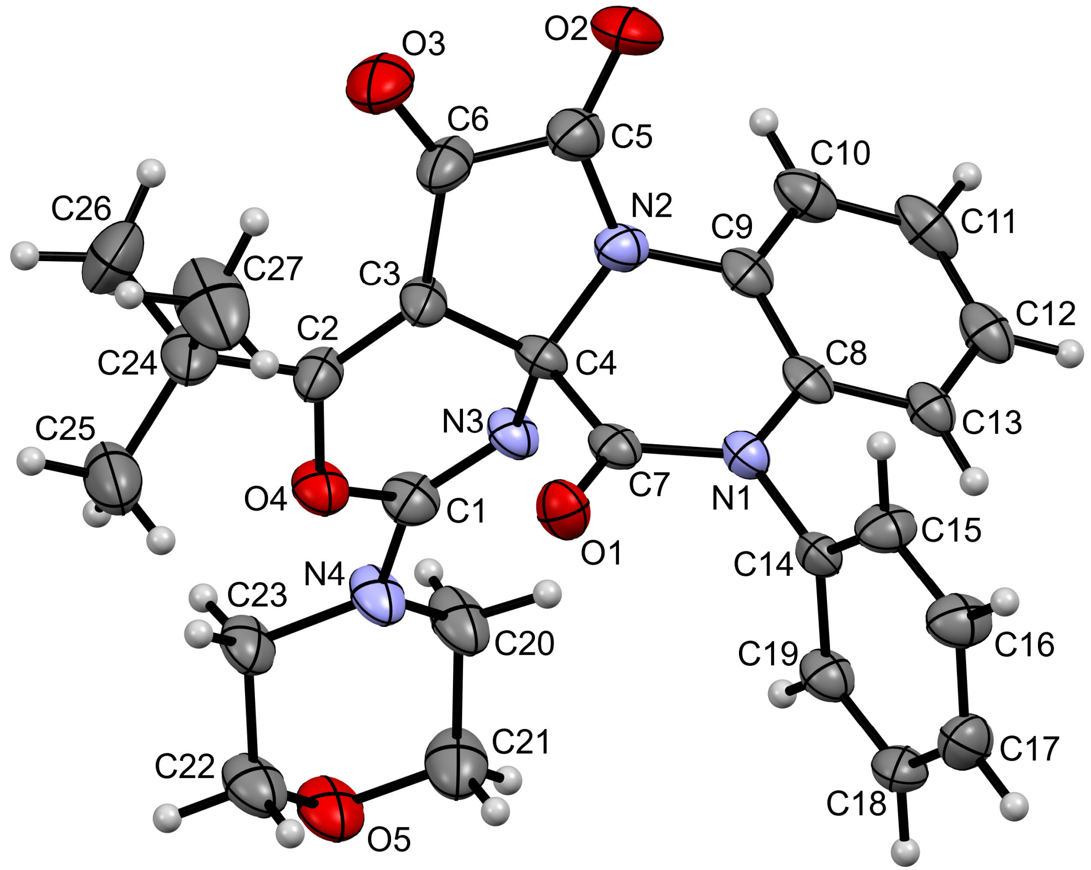

Figure S2. Molecular structure of compound **3i** showing 30% probability amplitude displacement ellipsoids (CCDC 2192397).

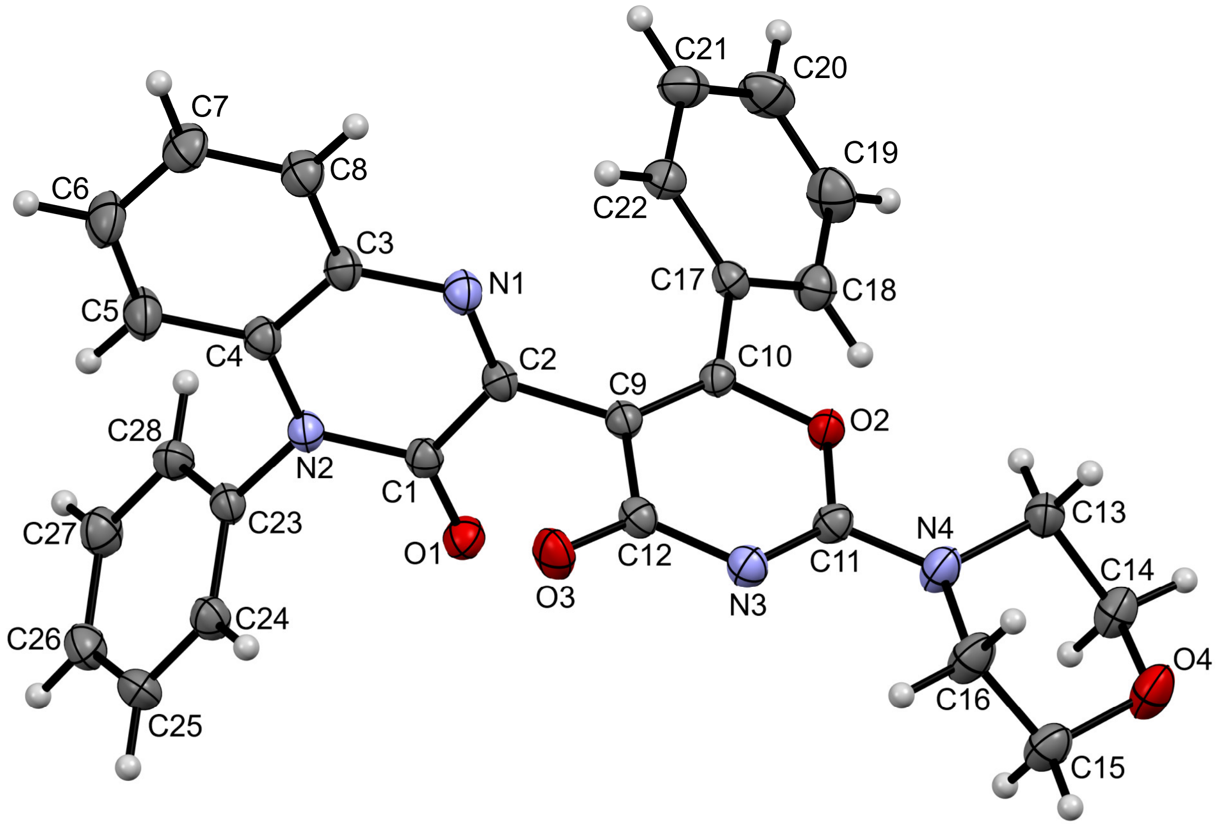

Figure S3. Molecular structure of compound **4b** showing 30% probability amplitude displacement ellipsoids (CCDC 2192400).

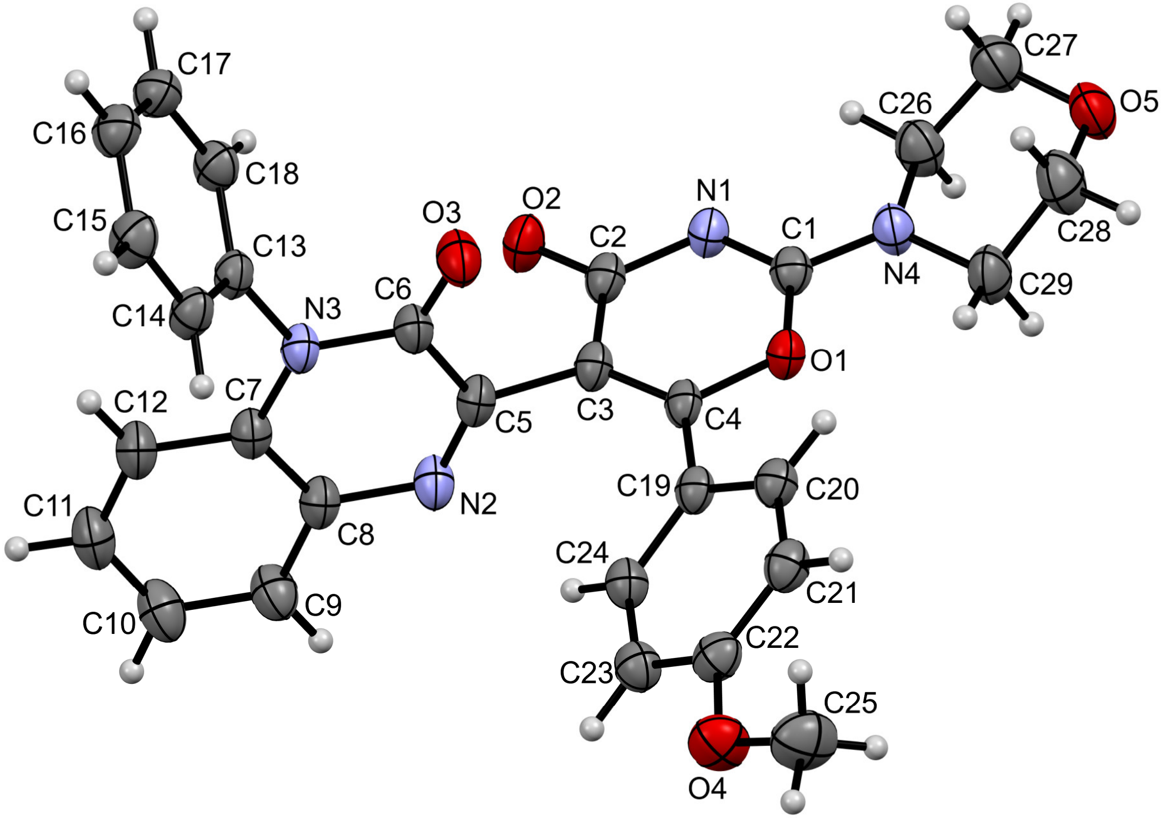

Figure S4. Molecular structure of compound **4f** showing 30% probability amplitude displacement ellipsoids (CCDC 2192399).

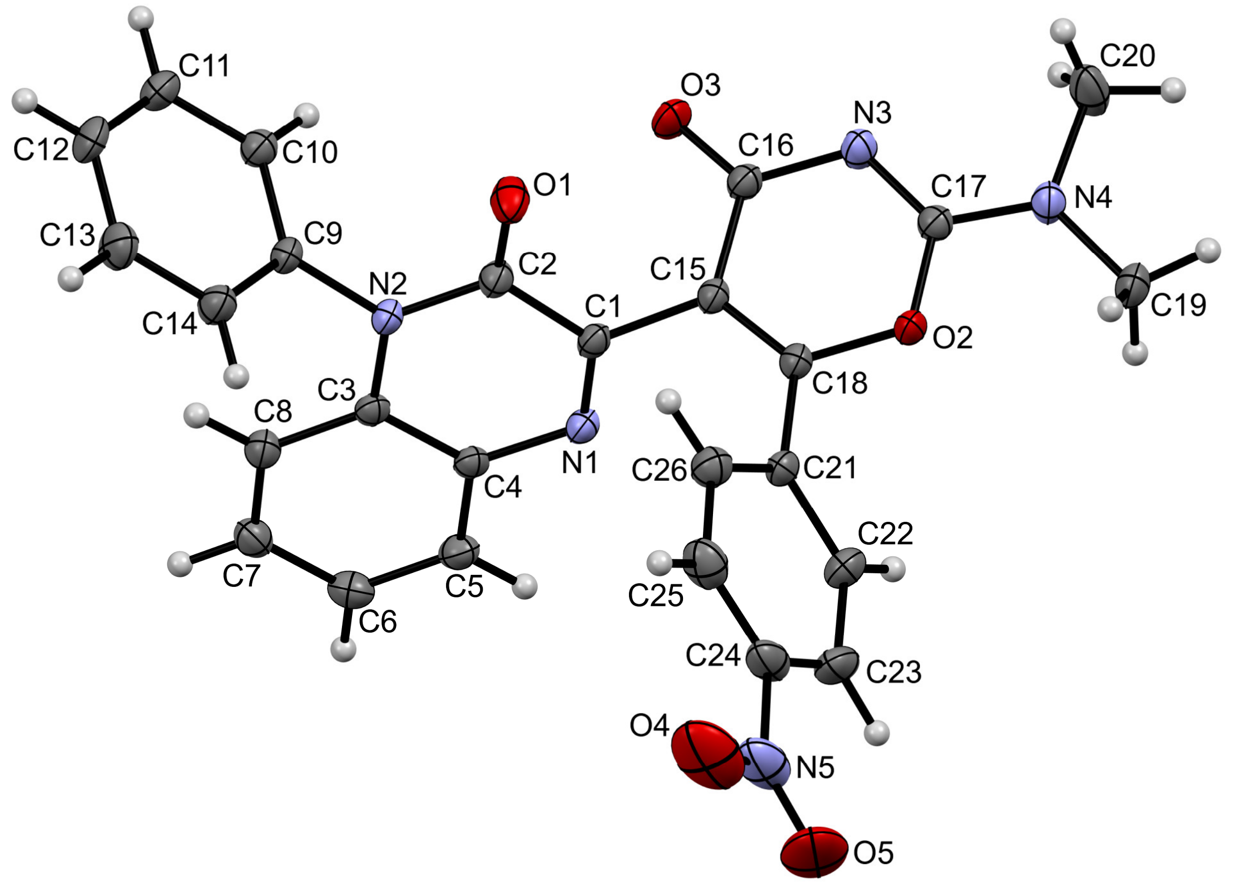

Figure S5. Molecular structure of compound **4g** showing 30% probability amplitude displacement ellipsoids (CCDC 2192398).

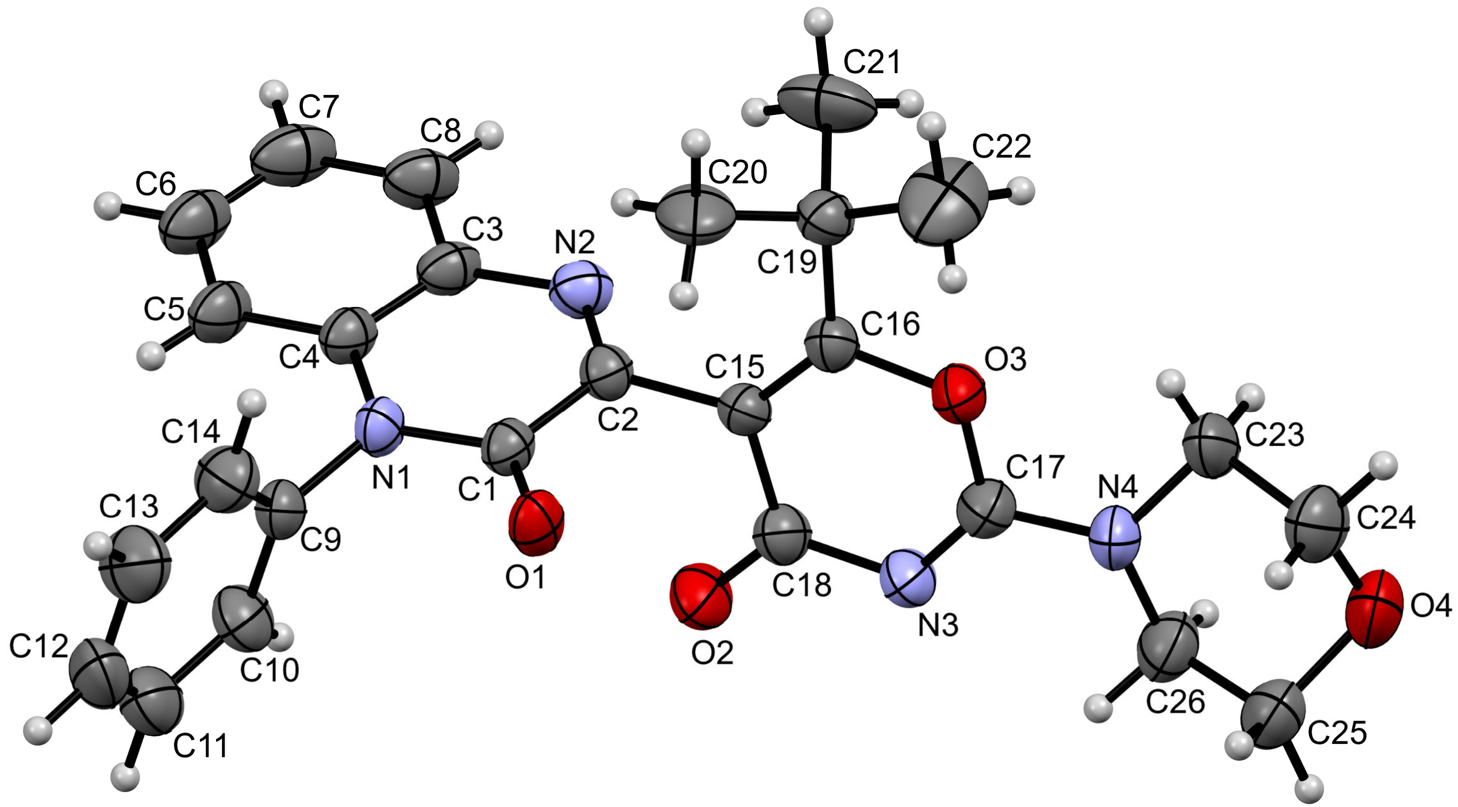

Figure S6. Molecular structure of compound **4i** showing 30% probability amplitude displacement ellipsoids (CCDC 2196232).
